# IL‐27 produced during acute malaria infection regulates *Plasmodium*‐specific memory CD4 ^+^ T cells

**DOI:** 10.15252/emmm.202317713

**Published:** 2023-10-19

**Authors:** Maria Lourdes Macalinao, Shin‐Ichi Inoue, Sanjaadorj Tsogtsaikhan, Hirotaka Matsumoto, Ganchimeg Bayarsaikhan, Jiun‐Yu Jian, Kazumi Kimura, Yoshiaki Yasumizu, Tsuyoshi Inoue, Hiroki Yoshida, Julius Hafalla, Daisuke Kimura, Katsuyuki Yui

**Affiliations:** ^1^ School of Tropical Medicine and Global Health Nagasaki University Nagasaki Japan; ^2^ Department of Infection Biology, Faculty of Infectious and Tropical Diseases London School of Hygiene and Tropical Medicine London UK; ^3^ Division of Immunology, Department of Molecular Microbiology and Immunology, Graduate School of Biomedical Sciences Nagasaki University Nagasaki Japan; ^4^ School of Information and Data Sciences Nagasaki University Nagasaki Japan; ^5^ Department of Experimental Immunology, Immunology Frontier Research Center Osaka University Suita Japan; ^6^ Integrated Frontier Research for Medical Science Division, Institute for Open and Transdisciplinary Research Initiatives (OTRI) Osaka University Osaka Japan; ^7^ Department of Physiology of Visceral Function and Body Fluid, Graduate School of Biomedical Sciences Nagasaki University Nagasaki Japan; ^8^ Division of Molecular and Cellular Immunoscience, Department of Biomolecular Sciences, Faculty of Medicine Saga University Saga Japan; ^9^ Shionogi Global Infectious Diseases Division, Institute of Tropical Medicine Nagasaki University Nagasaki Japan

**Keywords:** CD4^+^ T cells, IL‐27, immunological memory, malaria, Th1, Immunology, Microbiology, Virology & Host Pathogen Interaction

## Abstract

Malaria infection elicits both protective and pathogenic immune responses, and IL‐27 is a critical cytokine that regulate effector responses during infection. Here, we identified a critical window of CD4^+^ T cell responses that is targeted by IL‐27. Neutralization of IL‐27 during acute infection with *Plasmodium chabaudi* expanded specific CD4^+^ T cells, which were maintained at high levels thereafter. In the chronic phase, *Plasmodium‐*specific CD4^+^ T cells in IL‐27‐neutralized mice consisted mainly of CD127^+^KLRG1^−^ and CD127^−^KLRG1^+^ subpopulations that displayed distinct cytokine production, proliferative capacity, and are maintained in a manner independent of active infection. Single‐cell RNA‐seq analysis revealed that these CD4^+^ T cell subsets formed independent clusters that express unique Th1‐type genes. These IL‐27‐neutralized mice exhibited enhanced cellular and humoral immune responses and protection. These findings demonstrate that IL‐27, which is produced during the acute phase of malaria infection, inhibits the development of unique Th1 memory precursor CD4^+^ T cells, suggesting potential implications for the development of vaccines and other strategic interventions.

## Graphical abstract

IL‐27 inhibits the generation of parasite‐specific memory CD4^+^ T cells in a malaria infection model. Transient blockade of IL‐27 elicits high levels of unique CD127^+^ and KLRG1^+^ memory CD4^+^ T cells, elevates antibody production, and enhances protective immunity against secondary infection.

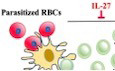

**The paper explained**

**Problem**

Immunity to malaria tends to decline along with the reduction in parasite exposure. It is not clear how the generation and maintenance of immunological memory is regulated during malaria infection.

**Results**

In this paper, we elucidated the role of the cytokine IL‐27 in modulating CD4^+^ T cell memory during malaria infection. Transient inhibition of IL‐27 during acute malaria expanded unique Th1‐type memory CD4^+^ T cells that are maintained during chronic infection independent of active infection. Moreover, IL‐27‐neutralized mice exhibited enhanced immune responses and protection.

**Impact**

Our results suggest that transient IL‐27 neutralization enhanced cellular and humoral immune responses against chronic malaria infection, which contributed to better protective immunity, thus demonstrating its potential application in vaccine development and other interventions.

## Introduction

Protective immunity to malaria develops after repeated infections over time in individuals living in endemic regions (Koch, 1900; Langhorne *et al*, 2008; Hafalla *et al*, 2011; Crompton *et al*, 2014; Doolan *et al*, 2009). In the initial phase of infection with *Plasmodium* parasites, specific T and B cells clonally expand upon recognition of the antigen and differentiate into effector lymphocytes playing crucial roles for the control of infection and disease development. In mouse models, *Plasmodium* infection results in the differentiation of specific CD4^+^ T cells into T helper 1 (Th1) and follicular T helper (Tfh) cells, which have important functions for the control of *Plasmodium* infection during blood‐stage infection (Perez‐Mazliah & Langhorne, 2014; Lonnberg *et al*, 2017). Th1 cells enhance the activation of phagocytic cells that participate in clearing parasites and infected RBCs (iRBCs), while Tfh cells support the differentiation of B cells to generate long‐lived high affinity antibody responses (Hansen *et al*, 2017; Kurup *et al*, 2019; Chan *et al*, 2020; Kumar *et al*, 2020). Immunity to malaria is, however, partial in reducing the parasites, and sterile immunity is hardly achieved by natural human infection with *P. falciparum* (Langhorne *et al*, 2008). Individuals acquire resistance to infection and disease after repeated infection over time through the development of host immune responses that can control parasitemia at a low density, and chronic infection status is established. Epidemiological studies in endemic areas suggest that chronic infection offer protection against newly inoculated malaria parasite infection (Smith *et al*, 1999). Experimental studies using *P. chabaudi chabaudi* AS (Pcc) infection, a murine model of chronic infection, demonstrated that persistent infection can maintain effector and memory CD4^+^ T cells, which effectively control re‐infection, and that the loss of protective immunity against blood‐stage infection correlate with the progressive decline in memory T cell responses (Achtman *et al*, 2007; Freitas do Rosario *et al*, 2008; Opata *et al*, 2018). Furthermore, continuous antigenic stimulation in chronic infection was suggested to enhance Th1 effector function and protective capacity of memory T cells in malaria in a model of TCR transgenic murine CD4^+^ T cells specific for MSP1 (Stephens & Langhorne, 2010). However, memory CD4^+^ T cells in chronically *Plasmodium*‐infected mice show altered function in which they produced cytokines but proliferated poorly upon re‐infection with homologous parasites (Opata & Stephens, 2017). It remains unclear how the generation and maintenance of immunological memory is regulated during chronic malaria infection as observed in endemic areas with stable transmission (Struik & Riley, 2004).

The induction and maintenance of memory T cells is regulated by various cytokines (Raeber *et al*, 2018). IL‐7 and IL‐15 are two major cytokines that are essential for survival and homeostatic proliferation of memory CD8^+^ and CD4^+^ T cells. IL‐2 signal during priming of CD8^+^ T cells is indispensable for their robust recall response upon secondary challenge (Williams *et al*, 2006). Type I interferons that are produced by inflammatory responses act on memory CD8^+^ T cells in the secondary response and enhance their ability to lyse target cells (Kohlmeier *et al*, 2010). IL‐10 and IL‐21 act together via STAT3 signaling pathway to promote memory CD8^+^ T cell differentiation and functional maturation during LCMV infection (Cui *et al*, 2011). IL‐27 is a heterodimeric cytokine of the IL‐12 family composed of p28 and EBI3 subunits and plays critical roles in the regulation of T cell responses (Pflanz *et al*, 2002; Hunter & Kastelein, 2012; Yoshida & Hunter, 2015). It is mainly secreted by dendritic cells and macrophages, while CD4^+^ T cells also produce IL‐27 during chronic infection such as malaria and tuberculosis (Xia *et al*, 2014; Yoshida & Hunter, 2015; Kimura *et al*, 2016). IL‐27 is inhibitory for IL‐2 production by CD4^+^ T cells, suppress development of Th17 cells, and promote effector CD4^+^ T cells to produce IL‐10 and is regulatory for the immune responses (Hunter & Kastelein, 2012). Along with this line, *IL‐27ra* ^−/−^ mice exhibited exacerbated Th1‐mediated immune response in an acute model of *P. berghei* infection and were susceptible to infection due to liver pathology despite efficient parasite clearance, indicating that IL‐27 regulates Th1 response during acute infection (Findlay *et al*, 2010; Villegas‐Mendez *et al*, 2013). Furthermore, *IL‐27ra* ^−/−^ mice that were infected with *P. berghei* NK65 and cured displayed improved parasite control during secondary infection with the homologous parasites, suggesting its role in the memory response (Gwyer Findlay *et al*, 2014). It is, however, unclear how IL‐27 regulates the generation and maintenance of memory immune responses.

We previously showed that *IL‐27ra* ^−/−^ mice that were infected with Pcc displayed reduced parasitemia during chronic phase and enhanced CD4^+^ T cell responses following rechallenge with heterologous *P. berghei* ANKA parasites (Sukhbaatar *et al*, 2020). In this study, we investigated the role of IL‐27 in the development of immunological memory to malaria during chronic *Plasmodium* infection using MHC‐II restricted TCR transgenic mouse, PbT‐II (Fernandez‐Ruiz *et al*, 2017; Enders *et al*, 2021). Our study suggests that IL‐27 produced during acute malaria infection inhibits the development of memory precursor CD4^+^ T cell subsets that are committed to Th1 memory CD4^+^ T cells. Inhibition of IL‐27 during acute infection allowed maintenance of higher levels of memory CD4^+^ T cells and antibody, contributing to better protection upon reinfection, which depended on persistence of the infection. Our findings indicate that IL‐27 is a key cytokine that regulates the generation of memory CD4^+^ T cells during chronic malaria infection.

## Results

### IL‐27 regulates the maintenance of memory PbT‐II cells

**Figure 1:**
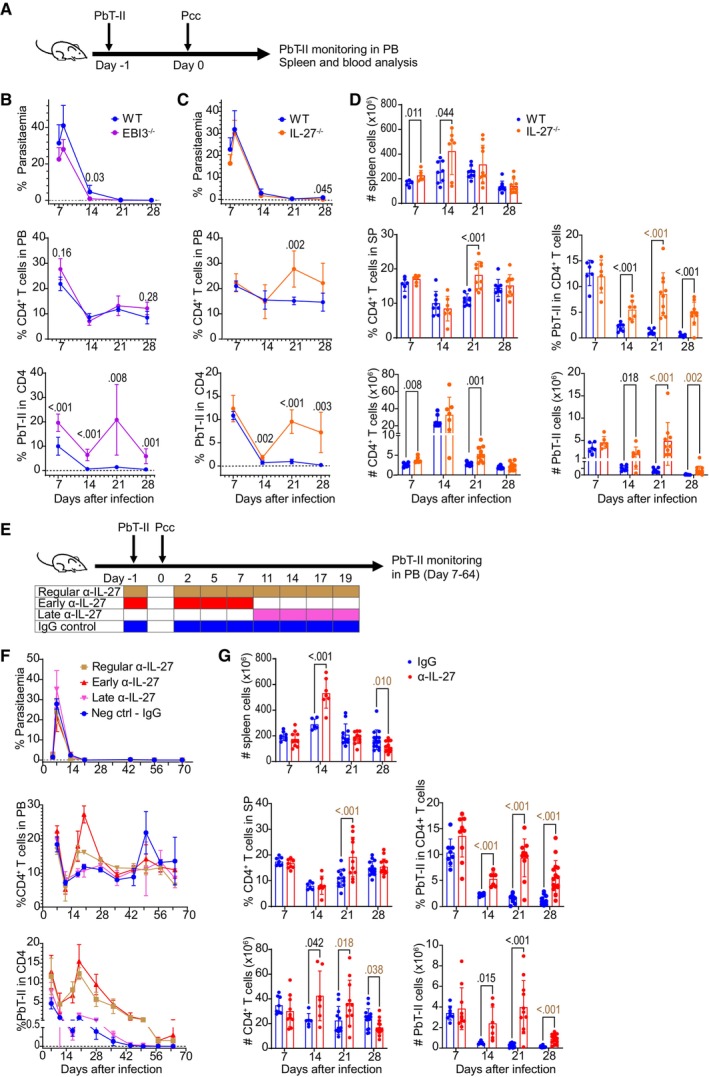
IL‐27 signaling regulates induction and maintenance of *Plasmodium*‐specific CD4^+^ T cells during chronic malaria infection A–DB6, *Ebi3* ^−/−^, and *Il27* ^−/−^ mice were transferred with PbT‐II cells a day before Pcc infection. Levels of parasitemia, proportion of PbT‐II cells in CD4^+^ T cells in peripheral blood (PB), as well as spleen (SP) profiles were monitored. Gating strategy for CD4^+^ T and PbT‐II cell analysis is described in Fig EV1A. (A) Experimental scheme for adoptive transfer of PbT‐II to B6, *Ebi3* ^−/−^, and *Il27* ^−/−^ mice. (B, C) Parasitemia levels and proportions of CD4^+^ T and PbT‐II cells in PB of B6 (WT) vs. *Ebi3* ^−/−^ mice (B; *n* = 4 mice/group) and B6 (WT) vs. *Il27* ^−/−^ mice (C; *n* = 4 mice/ group). Representative data of 2 independent experiments are shown. (D) CD4^+^ T and PbT‐II cell profile in the spleen of B6 (WT) vs. *Il27* ^−/−^ mice (*n* = 6, 8, 9, 9 for WT and *n* = 6, 7, 10, 10 for *Il27* ^−/−^ mice at day 7, 14, 21, 28 post infection (pi), respectively). Data are pooled from 2, 2, 3 and 3 independent experiments for day 7, 14, 21, and 28 timepoint, respectively.E, FAdoptive transfer of PbT‐II cells to B6 mice later infected with Pcc. Anti‐IL‐27 mAb was administered for regular, early, and late αIL‐27 groups at −1 to 19, −1 to 7, and 11 to 19 dpi, respectively, and IgG control group received IgG between −1 and 19 dpi. (E) Experimental scheme of IL‐27 neutralization with anti‐IL‐27 mAb. (F) Parasitemia levels and proportions of CD4^+^ T and PbT‐II cells in PB during weekly monitoring (*n* = 3 mice/group). Dose effect results were replicated in Fig EV1B–D.GAdoptive transfer of PbT‐II to Pcc‐infected B6 mice administered with either IgG or anti‐IL‐27 mAb during the early phase (between −1 and 7 days after infection). Total number of cells, proportion, and number of CD4^+^ T and PbT‐II cells in the spleen were monitored (*n* = 9, 6, 11, 15 for IgG‐treated and *n* = 9, 7, 11, 15 mice for anti‐IL‐27 mAb‐treated mice at day 7, 14, 21, 28 days pi, respectively). Data are pooled from 2, 2, 3 and 4 independent experiments for day 7, 14, 21, and 28 timepoint, respectively. Data information: Statistical significance in (B), (C), (D), and (G) was assessed by unpaired two‐tailed Student's *t*‐tests (*P* values (< 0.05) shown in black) or Mann–Whitney *U* test (*P* values (< 0.05) shown in brown) for comparing WT to EBI3^−/−^/Il27^−/−^ mice or IgG‐ to anti‐IL‐27 mAb‐treated mice per timepoint, depending on normality assessment. Error bars represent SD in all graphs. Source data are available online for this figure.

**Figure EV1:**
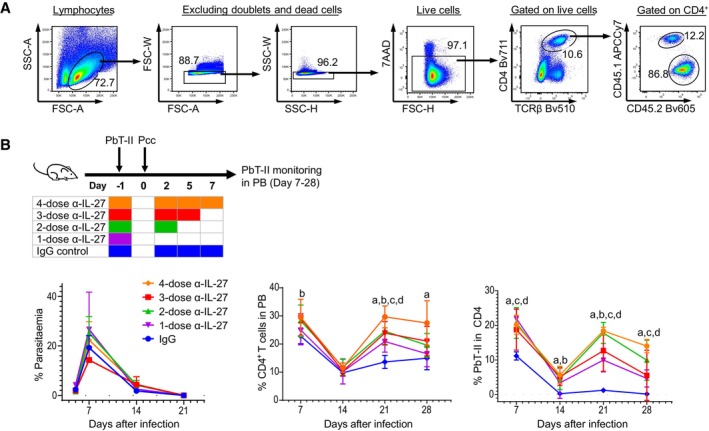
IL‐27 acts on PbT‐II cells during the early phase of infection. Related to Fig 1 Gating strategy for flow cytometry analysis of PbT‐II cells. Samples were stained for CD4, TCRβ, CD45.1, and CD45.2 to identify PbT‐II cells. APCCy7 anti‐CD45.1 and Bv605 anti‐CD45.2 mAbs for the congenic markers and Bv510 anti‐TCRβ mAb were maintained in all panels. For CD4 staining, Bv711 anti‐CD4 mAb was used in Figs 1, 2, and 6E, and EV2A and B; Pacific Blue‐anti‐CD4 mAb for Figs 2C and D, and 3, and 6E, and EV2C, and FITC anti‐CD4 mAb for Figs 2E and 6F and EV2D.B6 mice transferred with PbT‐II cells were administered with anti‐IL27 mAb in 4 different conditions or IgG control at timepoints indicated and were infected with Pcc (*n* = 4 mice/group). Parasitemia levels, proportion of CD4^+^ T cells, and proportions of PbT‐II cells in CD4^+^ T cells in PB were monitored. Representative data of two independent experiments are shown. Data information: Statistical significance was assessed by one‐way ANOVA followed by Tukey's multiple comparison test in (B) and by Student's *t* test in (C). *P* values (< 0.05) are shown. Small letters in (B) indicate significant differences compared to IgG control (a = 4‐dose, b = 3‐dose, c = 2‐dose, d = 1‐dose treatment), and *P*‐values are: day 7, b = 0.010; day 21, a = < 0.001, b = 0.015, c = 0.010, d = 0.022; day 28, a = 0.035 (middle graph); day 7, a = 0.031, c = 0.022, d = 0.009; day 14, a = 0.023, b = 0.029; day 21, a = < 0.001, b = 0.002, c = < 0.001, d = 0.018; day 28, a = 0.001, c = 0.044, d = 0.036 (right graph). Error bars represent SD.

We used CD4^+^ T cells from malaria antigen‐specific TCR transgenic mice, PbT‐II, to monitor antigen‐specific T cell response during infection with Pcc (Figs 1A and EV1A). Mice lacking IL‐27 due to a defect in p28 (*Il‐27* ^−/−^) or EBI3 (*Ebi3* ^−/−^) received PbT‐II cells and were infected with *P. chabaudi*. The proportions of CD4^+^ T cells in peripheral blood (PB) were significantly higher at day 7 and 28 compared to wild‐type (WT) for *Ebi3* ^−/−^ mice and at day 21 for *Il‐27* ^−/−^ mice, although parasitemia levels were comparable (Fig 1B and C). The difference between *Il‐27* ^−/−^ and *Ebi3* ^−/−^ may be due to the lack of IL‐35, heterodimer cytokine of EBI3 and p35, in *Ebi3* ^−/−^ mice (Yoshida & Hunter, 2015). The proportions of PbT‐II cells in PB CD4^+^ T cells peaked on day 7, declined by day 14 and were maintained at low levels in WT mice. A substantial increase in the number of PbT‐II cells was observed on day 21 in both *Il‐27* ^−/−^ and *Ebi3* ^−/−^ mice, which allowed for higher proportions to be maintained by day 28. In the spleen of *Il‐27* ^−/−^ mice, PbT‐II cells had consistently higher proportions and total counts on day 14–28 post infection (Fig 1D).

To establish whether neutralization of IL‐27 can lead to enhanced memory CD4^+^ T cell responses as observed in gene‐knock‐out mice, we administered anti‐IL‐27p28 neutralizing antibody in mice infected with Pcc. In initial experiments, we administered anti‐IL‐27 mAb from day −1 to day 19 of Pcc infection, covering the entire period in which the PbT‐II proportion increases, and found that PbT‐II cells increased comparably to those in gene knock‐out mice (Fig 1E and F). Since the proportion of PbT‐II cells increased in the absence of IL‐27 in two phases: early (day 0–7) and late (day 7–14) phases (Fig 1B and C), we administered anti‐IL‐27 mAb at different timepoints to determine the critical window of IL‐27 action on the kinetics of PbT‐II cells (Fig 1E). Mice treated with anti‐IL‐27 mAb in the early phase of the infection had higher proportions of PB PbT‐II cells comparable to continuously treated mice; those treated during late phase did not (Fig 1F). Further analysis showed that a 2‐dose treatment (days −1 and 2 post‐infection) was sufficient to observe the effect of IL‐27 neutralization, although the enhancing effect was reduced when compared with 4‐dose treatment (Fig EV1B). Therefore, the 4‐dose treatment (day −1, 2, 5, 7) was used for further experiments. Analysis of spleen cells in the treated mice showed consistently higher PbT‐II proportions and counts at day 14–28 post‐infection comparable to IL‐27‐deficient mice (Fig 1G). Altogether, these results indicate that transient IL‐27 neutralization during acute phase of Pcc infection enhances the levels of *Plasmodium*‐specific CD4^+^ T cells during chronic phase of Pcc infection, with comparable kinetics and magnitude to mice genetically deficient in IL‐27.

### Distinct subsets of memory CD4 ^+^ T cells were induced during chronic infection by the IL‐27 neutralization

**Figure 2:**
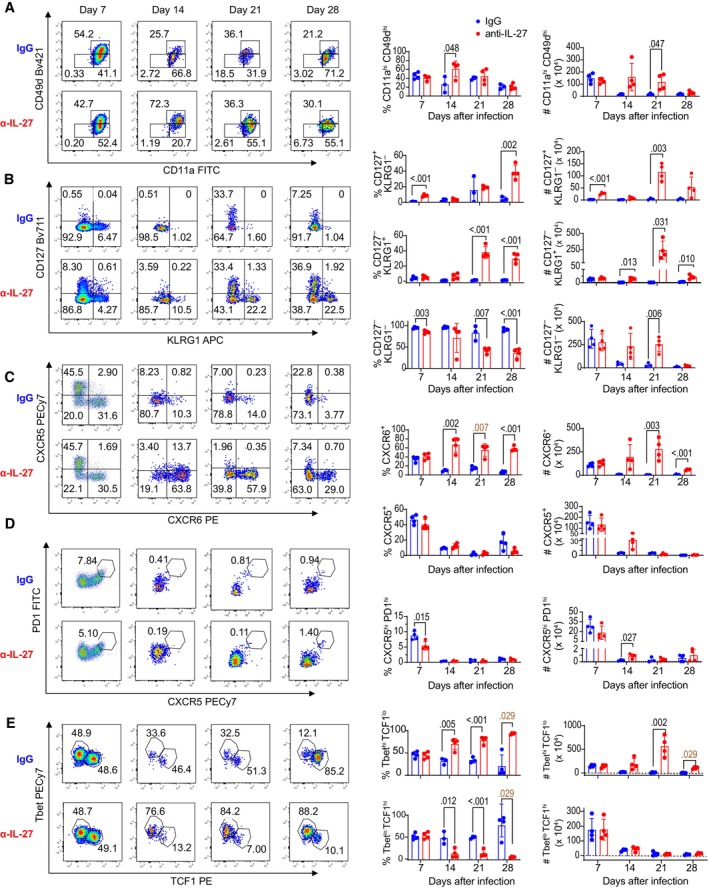
Inhibition of IL‐27 signaling affects the phenotype of *Plasmodium‐*specific CD4^+^ T cells during chronic infection B6 mice were transferred with PbT‐II cells and treated with either control (IgG, blue) or anti‐IL‐27 mAb (α‐IL‐27, red) between −1 and 7 days post‐Pcc infection. Splenic PbT‐II (CD45.1^+^CD45.2^−^TCRβ^+^CD4^+^) cells were analyzed by flow cytometry at 7, 14, 21, and 28 dpi.
A–DRepresentative flow cytometry profiles (left) of cell surface expression of CD11a/CD49d (A), CD127/KLRG1 (B), CXCR5/CXCR6 (C), and PD‐1/CXCR5 (D) on PbT‐II cells from control (blue) and α‐IL‐27 (red) mouse groups, and corresponding summary of the frequencies and total numbers of PbT‐II cell subpopulations for mouse groups (right).ERepresentative flow cytometry profiles (left) of expression of T‐bet and TCF1 in PbT‐II cells and corresponding summary of the frequencies and total numbers of PbT‐II cell subpopulations (right). Data information: Numbers in flow cytometry profiles indicate PbT‐II proportions (%) within each area. *n* = 4, 3, 3, 4 for IgG‐treated and *n* = 4, 4, 4, 4 mice for anti‐IL‐27 mAb‐treated mice at day 7, 14, 21, 28 days pi, respectively. Data for each timepoint are representative of 2, 2, 3, and 4 independent experiments for 7, 14, 21, 28 dpi, respectively. Statistical significance assessed by Student's *t* test (*P* values (< 0.05) shown in black) or Mann–Whitney *U* test (*P* values (< 0.05) shown in brown) per time point, depending on normality assessment. Error bars represent SD. Source data are available online for this figure.

**Figure EV2:**
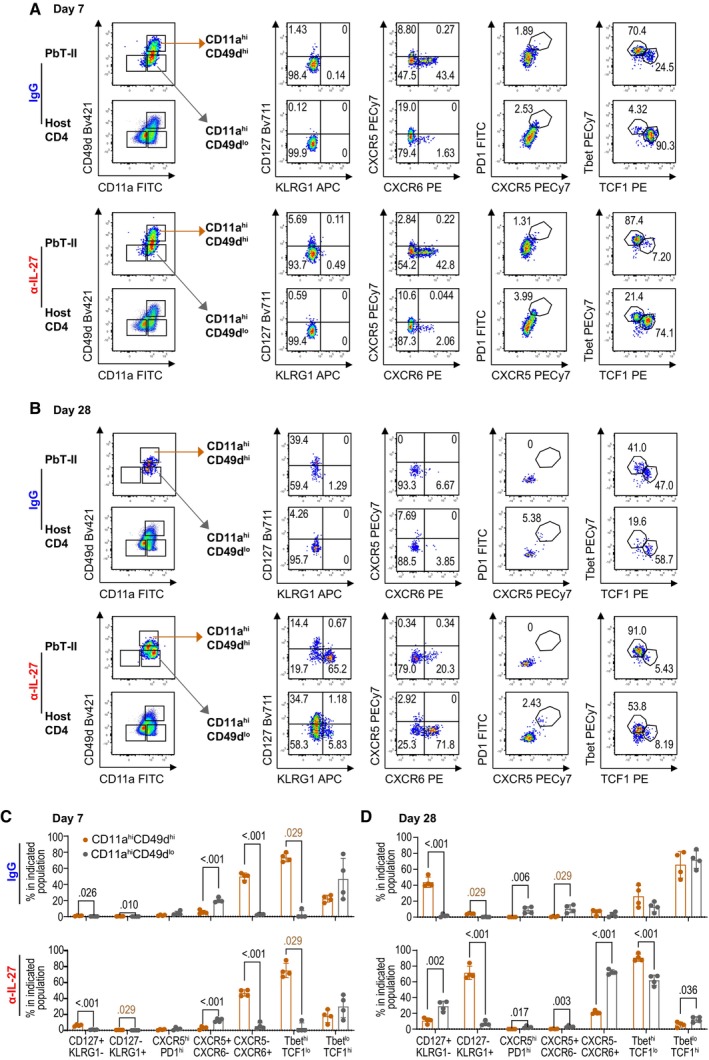
IL‐27 affects development of PbT‐II cells during the transition from acute to chronic malaria infection Spleen cells were prepared from mice on day 7 (A, C) and 28 (B, D) pi and were stained with CD11a and CD49d, along with other markers described in Fig 2 (*n* = 4 mice/group).
A, BRepresentative CD11a/CD49d profiles of CD45.1^+^CD45.2^−^ (PbT‐II) and CD45.1^−^CD45.2^+^ (host CD4^+^ T) cells (left) and flow cytometry profiles of PbT‐II subpopulations of CD11a^hi^CD49d^hi^ and CD11a^hi^CD49d^lo^ PbT‐II subpopulations are shown (right).C, DCorresponding summary frequencies of CD11a^hi^CD49d^hi^ and CD11a^hi^CD49d^lo^ PbT‐II subpopulations. Data information: Numbers in flow cytometry profiles indicate PbT‐II proportions (%) within each area. Statistical significance was assessed by Student's *t* test (*P* values (< 0.05) shown in black) or Mann–Whitney *U* test (*P* values (< 0.05) shown in brown) per time point, depending on normality assessment. Error bars represent SD.

To evaluate how IL‐27 signaling affects the development of PbT‐II cells during Pcc infection, we characterized the phenotype of these cells (Figs 2 and EV2). The proportions of antigen‐experienced CD11a^hi^CD49d^hi^ PbT‐II cells in the spleen were higher in anti‐IL‐27 mAb‐treated mice on days 14 and 21, and the numbers were higher on days 21 and 28 (Fig 2A). The CD127^+^KLRG1^−^ and CD127^−^KLRG1^+^ phenotypes are markers of long‐lived memory precursor and short‐lived effector CD8^+^ T cells, respectively (Kaech & Cui, 2012). Most PbT‐II cells in the control mice were CD127^−^KLRG1^−^ from day 7 to day 28. In anti‐IL‐27 mAb‐treated mice, the proportion of CD127^+^KLRG1^−^ PbT‐II cells was higher when compared with those in control on day 7. On day 28 of infection, CD127^+^KLRG1^−^ and CD127^−^KLRG1^+^ PbT‐II cells were dominant and reached 39.0 ± 1.4% and 39.9 ± 6.5%, respectively (Fig 2B). The proportions of CXCR5^−^CXCR6^+^ and CXCR5^+^CXCR6^−^ PbT‐II cells, putative Th1 and Tfh cells, respectively (Kim *et al*, 2001; Crotty, 2011), were comparable on day 7 in anti‐IL‐27 mAb‐treated and control mice (Fig 2C). The CXCR5^−^CXCR6^+^ PbT‐II cells increased and were maintained at higher levels through day 28 in anti‐IL‐27 mAb‐treated mice, while they decreased after day 14 in control mice. The proportions of CXCR5^+^ PbT‐II cells were maintained at low levels after day 14 in both groups as were the proportion of CXCR5^hi^PD1^hi^ germinal center (GC) Tfh PbT‐II cells. The difference in expression of these surface markers was also observed in PB PbT‐II subsets (Appendix Fig S1). PbT‐II cells expressing transcription factors associated with Th1 and Tfh/memory, T‐bet and TCF‐1, respectively, were not significantly different on day 7, while the proportion of T‐bet^hi^TCF‐1^lo^ cells increased in anti‐IL‐27 mAb‐treated mice from day 14 onward reaching 93.4 ± 1.9% on day 28; however, total numbers of Tbet^lo^TCF‐1^hi^ PbT‐II cells in the spleen were comparable (Fig 2E). The expression of Tfh‐associated transcription factor Bcl6 was also comparable at days 7 and 28, further suggesting that the Tfh subset was not affected by the IL‐27 neutralization (Appendix Fig S1).

Additionally, we also analyzed phenotypes of CD11a^hi^CD49d^hi^ and CD11a^hi^CD49d^lo^ PbT‐II subpopulations 7 and 28 days after infection to determine whether they represent Th1 and Tfh cells as we previously reported (Jian *et al*, 2021; Fig EV2). On day 7 after infection, majority of CD11a^hi^CD49d^hi^ PbT‐II cells were Th1 type, while Tfh type cells were dominant in CD11a^hi^CD49d^lo^ cells in both anti‐IL‐27 mAb‐ and IgG‐treated mice (Fig EV2A and C). On day 28, however, PbT‐II cells in anti‐IL‐27 mAb‐treated mice showed strong Th1 dominance in which the majority of CD49d^hi^CD49^hi^ and CD49d^hi^CD49^lo^ cells were KLRG1^+^T‐bet^hi^ and CXCR6^+^T‐bet^hi^, respectively, while both CD49d^hi^CD49^hi^ and CD49d^hi^CD49^lo^ PbT‐II cells were dominated by T‐bet^lo^TCF1^hi^ phenotype in IgG‐treated mice (Fig EV2B and D).

**Figure 3:**
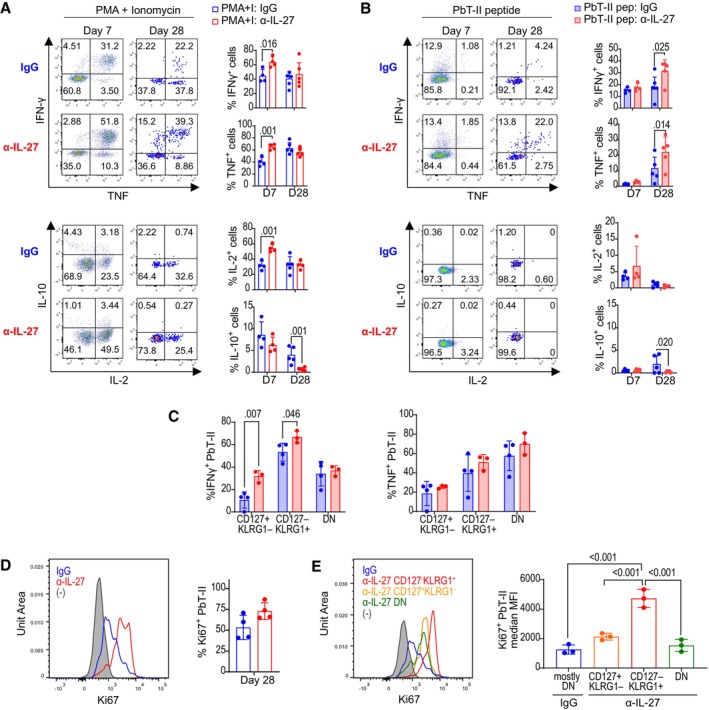
*Plasmodium*‐specific memory CD4^+^ T cells induced under IL‐27 neutralization exhibit enhanced cytokine production Cytokine production was analyzed for PbT‐II cells transferred to B6 mice infected with Pcc and were treated with control (IgG, blue) or anti‐IL‐27 mAb (α‐IL‐27, red) between −1 and 7 days after infection.
A, BSplenocytes from IgG and α‐IL‐27‐treated mice collected 7 and 28 days after infection were stimulated with PMA and ionomycin for 4 h (A), and splenic CD4^+^ T cells were cultured with PbT‐II peptide‐pulsed dendritic cells for 4 h (B), stained for surface markers, fixed, permeabilized, and stained for IFN‐γ, TNF, IL‐10, and IL‐2. Representative flow cytometry profiles (left) of cytokine expression of PbT‐II cells (CD45.1^+^CD45.2^−^TCRβ^+^CD4^+^) and frequencies of cytokine‐producing PbT‐II cells (right) are shown for control (blue) and anti‐IL‐27 mAb (red)‐treated mice at day 7 (*n* = 4 mice/group) and day 28 (*n* = 5 mice/group) days pi. Representative data of two independent experiments are shown.CProportions of IFN‐γ‐ or TNF‐producing cells in response to PbT‐II peptide within indicated subpopulations of control (*n* = 4) and anti‐IL‐27 mAb‐treated (*n* = 3) mice.D, ESplenic cells were prepared 28 days after Pcc infection and stained for TCRβ, CD4, CD45.1, CD127, and KLRG1, fixed, permeabilized, and stained for Ki67 to analyze expression in PbT‐II cell subpopulations.DRepresentative histogram plots (left) and summary graphs (right) of Ki67 expression in PbT‐II cells, and isotype control of IgG group (gray) at 28 days pi (*n* = 4 mice/each group). Data are representative of three independent experiments.ERepresentative histograms (left) and summary graph (right) of median fluorescence intensity (MFI) levels of Ki67 expression of total PbT‐II cells from IgG, and CD127^+^KLRG1^−^ (orange), CD127^−^KLRG1^+^ (red), CD127^−^KLRG1^−^ (green) PbT‐II subpopulations from anti‐IL‐27 mAb‐treated mice (*n* = 3 mice/each group). Data are representative of three independent experiments. Data information: Statistical significance was assessed by Student's *t* test for comparing IgG and anti‐IL‐27 mAb‐treated mouse groups in (A), (B), and (D), and per subpopulation in (C), and one‐way ANOVA followed by Tukey's multiple comparison test in (E). *P* values (< 0.05) are shown. Error bars represent SD in all graphs. Source data are available online for this figure.

To examine the functional activity of PbT‐II cells, we analyzed the cytokines produced as well as their proliferative phenotype (Fig 3). The proportions of PbT‐II cells producing IFN‐γ and TNF in response to PMA and ionomycin were higher in anti‐IL‐27 mAb‐treated mice on day 7, while those in response to antigenic peptide was not significantly different (Fig 3A and B). On day 28, however, PbT‐II cells exhibited significantly higher production of IFN‐γ and TNF in response to the peptide in anti‐IL‐27 mAb‐treated mice, consistent with the higher proportion of Th1 type PbT‐II cells on day 28 (Fig 3B). Production of IL‐2 and IL‐10 was mostly comparable, except for the higher IL‐2 production in response to PMA and ionomycin on day 7 and reduced IL‐10 production in response to peptide in anti‐IL‐27 mAb‐treated mice on day 28. Within PbT‐II subpopulations on day 28, the proportion of IFN‐γ‐producing cells was highest in CD127^−^KLRG1^+^ cells, while production of TNF was the highest in CD127^−^KLRG1^−^ PbT‐II cells (Fig 3C). When compared with IgG‐treated mice, CD127^+^KLRG1^−^ and CD127^−^KLRG1^+^ subsets of PbT‐II cells in anti‐IL‐27 mAb‐treated mice produced more IFN‐γ. Proliferation was enhanced in anti‐IL‐27 mAb‐treated mice compared to control on day 28 as evaluated by Ki67 expression (Fig 3D). Among PbT‐II subpopulations, CD127^−^KLRG1^+^ cells showed highest Ki67 expression followed by CD127^+^KLRG1^−^ and CD127^−^KLRG1^−^ cells in anti‐IL‐27 mAb‐treated mice (Fig 3E). Altogether, these results show that neutralization of IL‐27 in the acute phase of the infection profoundly affected PbT‐II cell phenotype and function during chronic phase, although PbT‐II cells from anti‐IL‐27 mAb‐treated mice were phenotypically indistinguishable from control group during acute phase except for a slight but significant increase in CD127^+^ cells in anti‐IL‐27 mAb‐treated mice.

### Transcriptome analysis revealed distinct subsets of PbT‐II cells induced by IL‐27 neutralization during malaria

**Figure 4:**
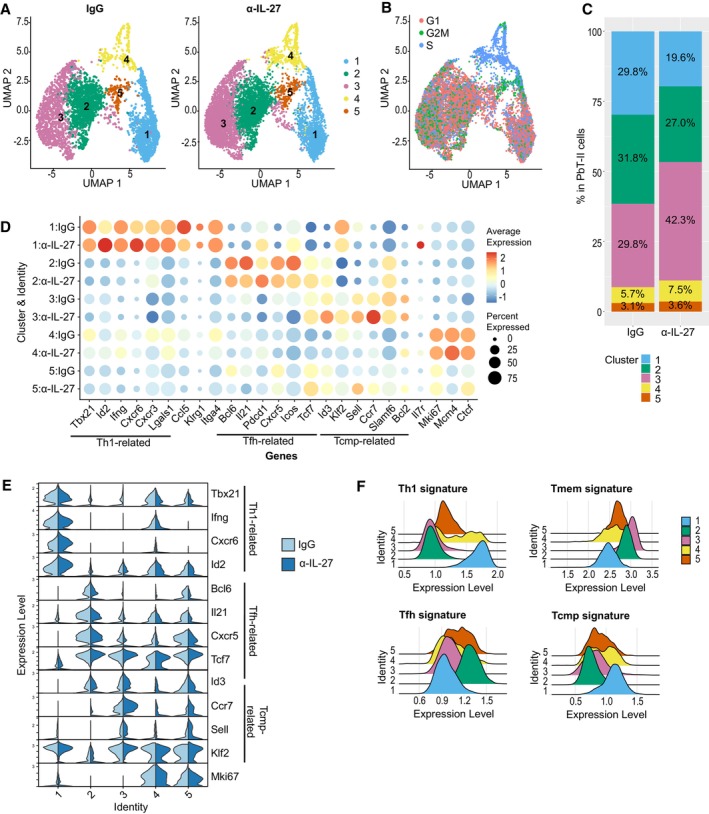
Early IL‐27 neutralization affects memory signature during acute phase of Pcc infection B6 mice were transferred with PbT‐II cells, infected with Pcc, and treated with control (IgG; *n* = 1 biological replicate) or anti‐IL‐27 mAb (α‐IL‐27; *n* = 1 biological replicate) between −1 and 5 days of infection. PbT‐II cells were purified from these mice 7 days after infection and single‐cell RNA sequencing (scRNA‐seq) analysis was performed. Details of the experiments are shown in Fig EV3A–C.
UMAP plots of PbT‐II cells from IgG control (*n* = 4,030) and anti‐IL‐27 mAb‐treated mice (*n* = 7,476) after unsupervised clustering of pooled single‐cell data from the two groups, with clusters colored by gene expression profiles.UMAP clustering of PbT‐II cells colored by cell cycle profiles.Summary graph of proportions of PbT‐II cells in each cluster for IgG and anti‐IL‐27 mAb‐treated mice in (A).Dot plots showing the expression of Th1‐, Tfh‐, Tcmp‐related genes (Ciucci *et al*, 2019), and other genes of interest in each UMAP cluster of PbT‐II cells from IgG and anti‐IL‐27 mAb‐treated mice. Dot colors represent the intensity of expression, while dot size represents the proportion of cells with the corresponding expression.Violin plots showing the expression of Th1‐, Tfh‐, Tcmp‐, and proliferation‐associated genes in PbT‐II cells from IgG (light blue) and anti‐IL‐27 mAb (blue) treated mice.Ridge plots showing the expression of published Th1, Tfh, Tmem, and Tcmp CD4^+^ T cell signatures in each of the UMAP clusters in (A) based on (Ciucci *et al*, 2019).
Source data are available online for this figure.

**Figure 5:**
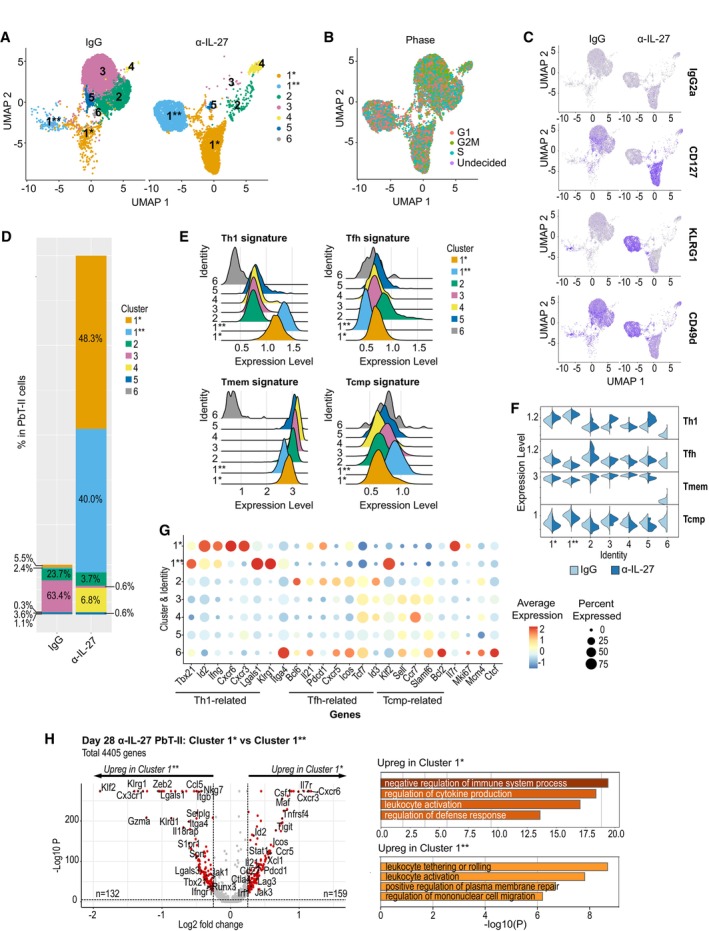
Transcriptome analysis reveals distinct T cell subsets during chronic infection induced by IL‐27 neutralization B6 mice were transferred with PbT‐II cells, infected with Pcc, and were treated with either IgG or anti‐IL‐27 mAb between −1 and 7 days after infection (*n* = 1 biological replicate per timepoint). PbT‐II cells were prepared from spleen at day 28 pi, stained for CD4/TCR/CD45.1 and for CD127, KLRG1, and CD49d with TotalSeq antibodies, sort purified, and processed for scRNA‐seq and CITE‐Seq analysis. Details of the experiment are found in Fig EV3A–C.
A–GComparative analysis of scRNA‐seq data from IgG and anti‐IL‐27 mAb‐treated PbT‐II cells. (A) UMAP plot colored of day 28 PbT‐II cells from IgG control (*n* = 7,491) and anti‐IL‐27 mAb‐treated mice (*n* = 4,944) after unsupervised clustering of pooled single cell data from the two groups, with clusters colored by gene expression profiles. Cluster labels were harmonized to reflect similar gene expression patterns in the clusters at day 7 pi (Fig 4) and anti‐IL27 mAb day 7–28 PbT‐II analysis (Fig EV4). (B) UMAP clustering of PbT‐II cells colored by cell cycle profiles. (C) CITE‐seq analysis of PbT‐II cells for IgG2a (isotype control), CD127, KLRG1, and CD49d, shown in the same UMAP clustering as (A). (D) Proportions (%) of each cluster within PbT‐II cells, with bar graph sizes shown relative to the total number of PbT‐II cells in IgG (36.8 × 10^4^) and anti‐IL‐27 mAb treated (265.7 × 10^4^) mice. (E) Ridge plots of PbT‐II cells showing the expression of published CD4^+^ T cell signature genes (Ciucci *et al*, 2019). (F) Violin plots comparing the expression of the CD4^+^ T cell signature genes. (G) Dot plots showing the expression of Th1‐, Tfh‐, Tcmp‐, and proliferation‐associated genes in each cluster. Dot colors represent the intensity of expression, while dot size represents the proportion of cells with the corresponding expression.HVolcano plot of differentially expressed genes between major clusters 1* and 1** within PbT‐II cells from anti‐IL‐27‐treated mice and corresponding Gene Ontology enrichment analysis for the upregulated genes in each group using Metascape.
Source data are available online for this figure.

**Figure EV3:**
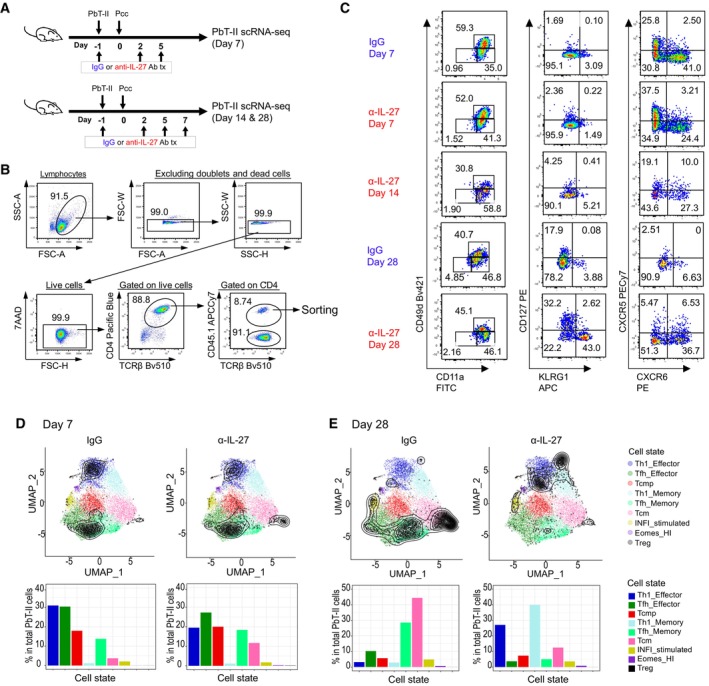
scRNAseq analysis shows Th1‐biased CD4^+^ T cell development during malaria chronic infection. Related to Figs 4 and 5 B6 mice were transferred with PbT‐II cells, treated with IgG or anti‐IL‐27 mAb on day −1, 2 and 5 for day 7 analysis, while mice were treated with anti‐IL‐27 mAb on day −1, 2, 5, and 7 for day 14 and 28 analysis (*n* = 1 biological replicate per timepoint). PbT‐II cells were purified and subjected to single‐cell RNA sequencing (scRNA‐seq) and CITE‐seq analysis. The ProjecTILs algorithm (Andreatta *et al*, 2021) was used to analyze CD4^+^ T cell states of PbT‐II cells based on a published reference atlas (Andreatta *et al*, 2022).
AExperimental scheme.BGating strategy for the sorting of PbT‐II cells for the scRNA‐seq experiments: Spleen cells were stained for CD4, TCRβ, and CD45.1 to distinguish PbT‐II cells and for TotalSeq IgG2a, CD127, KLRG1, and CD49d for CITE‐seq analysis.CFlow cytometry profiles for each PbT‐II sample analyzed for single‐cell transcriptomics.D, EPredicted distribution of the projected PbT‐II cells in IgG and anti‐IL‐27 mAb‐treated mice on day 7 (D) and day 28 (E) after Pcc infection as density contours in a UMAP plot of a CD4^+^ T cell reference map (Andreatta *et al*, 2022). The bar graphs represent the proportions of the PbT‐II cells projected in the indicated reference subtype.

The effect of IL‐27 neutralization suggested that the development of memory precursor subpopulations could be inhibited by IL‐27. To uncover the responding CD4^+^ T cell subsets at the transcriptional level, we performed single‐cell RNA‐seq (scRNA‐seq) and compared PbT‐II cells from anti‐IL‐27 mAb‐ and control IgG‐treated mice on day 7 and 28 after infection (Figs 4, 5 and EV3). Dimensional reduction and clustering of the cells on day 7 of infection based on their gene expression profiles identified 5 clusters: *Tbx21* ^+^ *Id2* ^+^ *Ifng* ^+^ *Cxcr6* ^+^ *Cxcr3* ^+^ cells (cluster 1) with high Th1 as well as T central memory precursor (Tcmp) signature scores, *Bcl6* ^+^ *Id3* ^+^ *Il21* ^+^ *Cxcr5* ^+^ cells (cluster 2) with high Tfh signature scores, *Tcf7* ^+^ *Klf2* ^+^ *Sell* ^+^ *Ccr7* ^+^ cells (Cluster 3) with high memory T cell (Tmem) signature scores, *Mki67* ^+^ *Mcm4* ^+^ *Ctcf* ^+^ cells (Cluster 4) in the S phase of the cell cycle, and cells with low expression in most of the analyzed genes, including those in G2/M phase (Cluster 5; Fig 4A, B and D–F). While CD127^+^ cells were slightly increased in anti‐IL‐27 mAb‐treated mouse, this population (*Il7r* ^+^) belongs to cluster 1 (Th1; Figs 2B and 4D). There were variations in the expression among individual genes in each cluster by anti‐IL‐27‐mAb treatment such as increase in *Id2* and *Il7r* in cluster 1 and *Id3* and *Ccr7* in cluster 3, as well as increase in *Bcl2* in minor clusters 4 and 5 (Fig 4D and E). In addition, we observed a shift in the proportions of cells in clusters 1 and 3 between 2 groups (Fig 4C) and higher Tcm population in anti‐IL‐27 mAb‐treated mouse in reference mapping approach (Fig EV3D), suggesting an effect of anti‐IL‐27 mAb‐treatment at day 7 after Pcc infection.

**Figure EV4:**
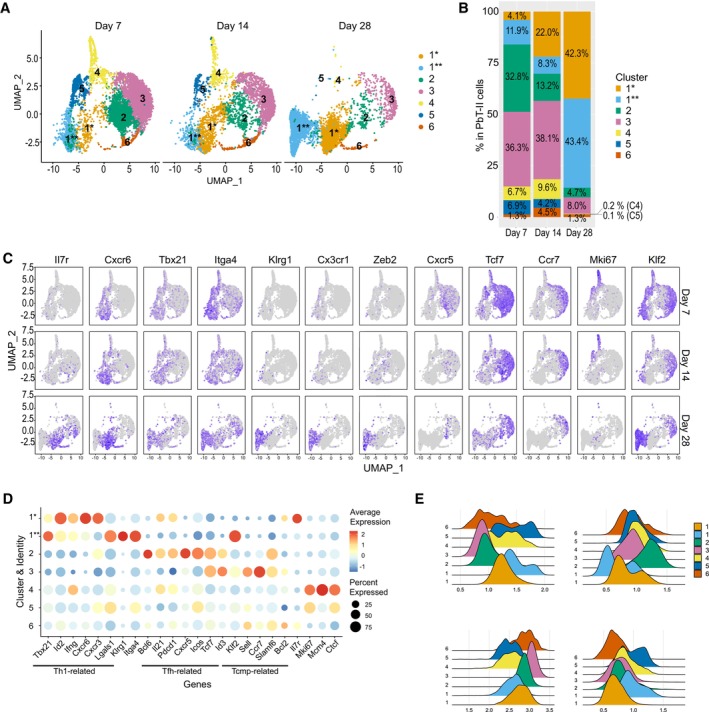
scRNA‐seq analysis suggests transition of CD4^+^ T cell subsets under IL‐27‐neutralization condition. Related to Fig 5 scRNA‐seq data of PbT‐II cells from Pcc‐infected anti‐IL‐27 mAb‐treated mice (day7, 14, and 28) were pooled, and unsupervised clustering was performed.
UMAP plot colored by gene expression clustering.Proportions (%) of each cluster for each time point.Feature plots of indicated genes across cell clusters as distributed in UMAP plots.Dot plots showing the expression of Th1‐, Tfh‐, Tmem‐, and proliferation‐associated genes in each cluster. Dot colors represent the intensity of expression, while dot size represents the proportion of cells with the corresponding expression.Ridge plots of PbT‐II cell clusters showing the expression of published CD4^+^ T cell signature genes (Ciucci *et al*, 2019).

We next analyzed the scRNA‐seq data of PbT‐II cells anti‐IL‐27 mAb‐ and IgG‐treated mice on day 28 after infection (Figs 5 and EV3A–C and E). Dimensional reduction and clustering of the cells based on their gene expression profiles identified 7 clusters and their surface markers were visualized by CITE‐seq analysis (Fig 5A–G). For consistency, we numbered these clusters to correspond with the same‐numbered clusters on day 7 that exhibit similar gene signature (Fig 5A, C and E). Two major clusters exhibiting high Th1 signature scores in anti‐IL‐27‐treated mice were named cluster 1* and cluster 1** based on their relation to day 7's cluster 1, although these cells were rare in IgG control on day 28. Cluster 1* cells were CD127^+^KLRG1^−^ based on CITE‐Seq and showed high *Id2*, *Ifng*, *Cxcr6*, and *Cxcr3*; Cluster 1** cells were CD127^−^KLRG1^+^ and expressed *Tbx21*, *Lgals1*, *Itga4*, and *Klf2* at high levels and exhibited high Tcmp signature in addition to high Th1 score, suggesting their memory potential (Fig 5E–G). Comparison of the 2 clusters showed that cluster 1* cells are distinguished by their expression of co‐inhibitory genes (*Tigit*, *Lag3*, and *Pdcd1*) and chemokine receptors (*Cxcr6*, *Cxcr3*, and Ccr5), while cluster 1** express genes related to leukocyte migration (*Cx3cr1*, *Ccl5*, *and S1pr1*), killer cell lectin‐like receptors (*Klrg1* and *Klrd1*), and transcription factors (*Klf2*, *Zeb2*, and *Tbx2*) suggesting their differential function (Fig 5H). Clusters 2–6 were CD127^lo^KLRG1^lo^ (Fig 5A and C). Cluster 2 cells, although a minority in anti‐IL‐27 mAb‐treated mice, were present in similar absolute numbers in anti‐IL‐27 mAb‐ and IgG‐treated mice, exhibited a high Tfh signature score, and expressed genes including *Bcl6*, *IL21*, and *Cxcr5* (Fig 5A and D–G). Cluster 3 cells comprised the largest cluster in IgG‐treated mice, exhibited high Tmem score and expressed memory phenotype genes including *Tcf7*, *Sell*, *Ccr7*, and *Bcl2* (Fig 5A and D–G). Cluster 4 was the third largest population in anti‐IL‐27 mAb‐treated mice exhibiting highest Tmem score (Fig 5A, D and E). Interestingly, the expression levels of Th1 signature genes in cluster 3 and 5 cells in anti‐IL‐27‐treated mice were higher than those in IgG‐treated mice (Fig 5F). Cluster 6 was detected in IgG control alone. Mapping to the CD4^+^ T cell reference atlas (Andreatta *et al*, 2022) showed that the major populations in PbT‐II cells in the anti‐IL‐27 mAb‐treated mouse had Th1 effector and Th1 memory subsets, while those in the IgG‐treated mouse were Tcm and Tfh memory subsets (Fig EV4E). These features indicate the expansion of unique Th1‐type PbT‐II cells in Pcc‐infected mice when IL‐27 is neutralized during acute infection. Taken together, PbT‐II cells in anti‐IL‐27 mAb‐treated and IgG‐treated mice were comprised of clearly distinct subpopulations on day 28 of infection. The increase in the maintained PbT‐II populations in anti‐IL‐27 mAb‐treated mice at day 28 was due to two major populations of Th1‐like clusters 1* and 1**, and Tmem‐like cluster 4 as well as a minor Tfh cluster.

We next compared scRNA‐seq data of PbT‐II cells in anti‐IL‐27 mAb‐treated mice in transition from acute to chronic infection (Fig EV4). The scRNA‐seq data of PbT‐II cells from day 7, 14, and 28 after infection were dimensionally reduced and their clustering analysis revealed a total of 7 clusters, which were labeled with the same numbering applied to previous cluster analyses (Fig EV4A). Cluster 1* and 1**, both corresponding to Th1 cluster on day 7, increase with time and each occupied > 40% on day 28 (Fig EV4B). Cluster 2, corresponding to the Tfh cluster on day 7, was reduced on day 14 and became a minor cluster (< 5%) by day 28. Cluster 3, which corresponds to the Tmem‐like cells on day 7, were maintained through day 14 but became a minor population on day 28. These features suggested a gradual shift in the phenotypes of PbT‐II cells during transition from acute to chronic infection.

### Active infection is not essential for the maintenance of Th1‐type memory PbT‐II cells

**Figure 6:**
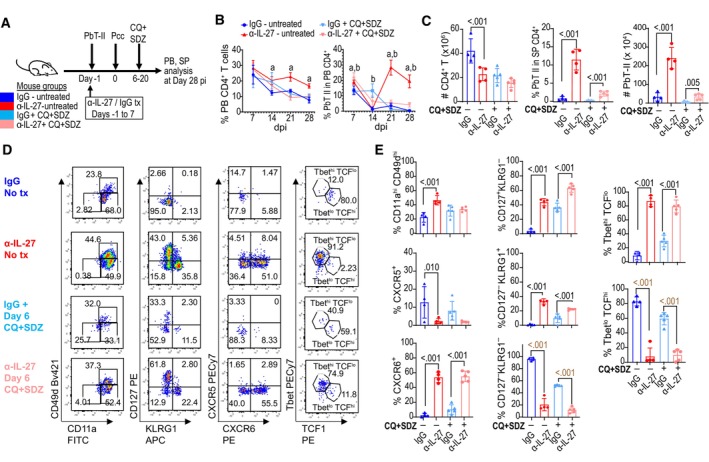
Enhanced memory PbT‐II cells induced by transient IL‐27 neutralization are maintained without live parasites during chronic phase B6 mice were transferred with PbT‐II cells, treated with IgG (blue, light blue) or anti‐IL‐27 mAb (red, pink), and treated (light, blue pink) or not treated (blue, red) with antimalarial drugs starting 6 days after Pcc infection.
Experimental scheme for antimalarial treatment experiment (*n* = 4 for antimalarial untreated groups and *n* = 5 mice for antimalarial‐treated groups).Kinetics of the proportion of CD4^+^ T cells in PB and of PbT‐II cells in PB CD4^+^ T cells during the course of Pcc infection. Small letters indicate significant differences (*P* < 0.05) between IgG vs. anti‐IL‐27 in antimalarial‐untreated (a), and ‐treated (b) mice.Total number of CD4^+^ T cells in the spleen and proportions of PbT‐II cells in CD4^+^ T cells and their total numbers in spleen on day 28 pi.Representative flow cytometry profiles of splenic PbT‐II cells on day 28 pi.Proportions of PbT‐II cells with the indicated phenotype on day 28 pi. Data information: Data are representative of 4 independent experiments. Statistical significance was assessed by Student's t test per time point for (B), and one‐way ANOVA followed by Tukey's multiple comparison test (*P* values (< 0.05) shown in black) or Kruskal–Wallis test with Dunn's *post hoc* tests (*P* values (< 0.05) shown in brown) for (C, E) depending on normality assessment. In (B), “a” corresponds to the *P*‐values of 0.002, < 0.001, and < 0.001 in the left graph and 0.012, < 0.001, and < 0.001 in the right graph for days 14, 21, and 28, respectively, while “b” corresponds to *P*‐values 0.001, 0.024, 0.002, and < 0.001 for days 7, 14, 21, and 28, respectively. Error bars represent SD. Source data are available online for this figure.

To determine whether live parasites are required for the induction and maintenance of memory CD4^+^ T cells, Pcc‐infected mice with and without anti‐IL‐27 mAb were treated with antimalarial drugs (Fig 6). Antimalarial drug treatment starting 6 days post‐infection did not have significant impact on the proportions of PbT‐II cells in control IgG group on day 28. In anti‐IL‐27 mAb‐treated mice, however, the increase in PbT‐II cells on day 21 after infection was not observed in mice also treated with antimalarial drugs, suggesting its dependence on active infection. Despite this lack of increase, the total number of PbT‐II cells remained higher in antimalarial‐treated anti‐IL‐27 mAb‐treated mice when compared with its IgG‐treated counterpart in both PB and spleen after 28 days of infection (Fig 6B and C). Phenotypically, PbT‐II cells in anti‐IL‐27 mAb‐treated mice exhibited higher proportions of CD127^+^KLRG1^−^, CD127^−^KLRG1^+^, CXCR6^+^, and T‐bet^hi^TCF‐1^lo^ cells than those in control IgG‐treated mice in both antimalarial drug‐treated and untreated mice (Fig 6D and E). These results suggest that the increase of PbT‐II cells between 14 and 21 days of infection is dependent on active infection, while phenotypical differentiation of PbT‐II cells during memory phase is independent of the presence of live parasites during chronic infection.

**Figure EV5:**
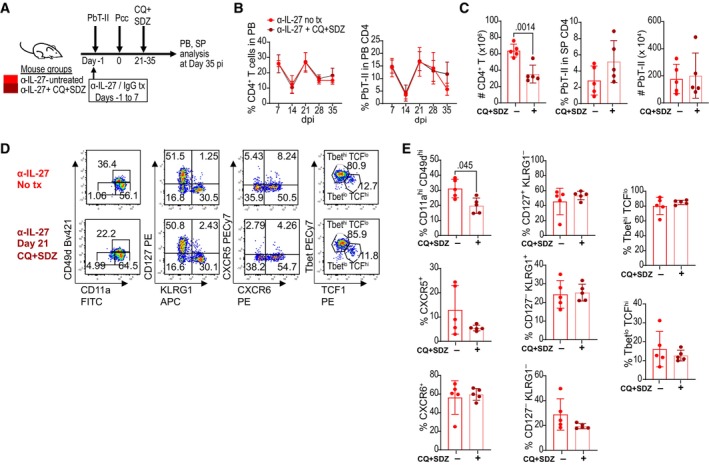
Antimalarial treatment during chronic phase of Pcc infection does not affect the persistence and phenotype of memory PbT‐II cells. Related to Fig 6 B6 mice were transferred with PbT‐II cells, treated with anti‐IL‐27 mAb, and treated (dark red) or not treated (red) with antimalarial drugs starting 21 days after Pcc infection (*n* = 5 biological replicates per treatment group). PB was monitored weekly and PbT‐II cells in the spleen were analyzed 35 days post‐Pcc infection.
Experimental scheme.Kinetics of proportions of CD4^+^ T cells in PB and of PbT‐II cells in PB CD4^+^ T cells.Total number of CD4^+^ T cells in spleen and proportions within CD4^+^ T cells and total number of PbT‐II cells in spleen (*n* = 5 mice/group).Representative flow cytometry profiles of splenic PbT‐II cells on day 35 of Pcc infection.Proportions of PbT‐II cells with the indicated phenotype and those expressing the indicated transcription factors 35 days after Pcc infection (*n* = 5 mice/group). Data information: Representative data of two independent experiments are shown. Statistical significance was assessed by Student's *t* test (*P* values (< 0.05) shown in black) or Mann–Whitney *U* test, depending on normality assessment. Error bars represent SD.

Next, we treated mice with antimalarial drug starting at day 21 post‐infection (Fig EV5). The proportion of PbT‐II cells in PB and the number of PbT‐II cells in spleen did not decrease, although the total number of CD4^+^ T cells decreased 14 days after infection (Fig EV5B and C). The proportions of PbT‐II cells expressing CD127, KLRG1, CXCR5, CXCR6, T‐bet, and TCF‐1 were not significantly different between antimalarial treated and untreated mice (Fig EV5D and E). In sum, these results imply that once memory CD4^+^ T cells are induced, their maintenance does not require live parasite infection for more than 14 days.

### Memory CD4 T cells induced by IL‐27 neutralization contribute to protective immunity

**Figure 7:**
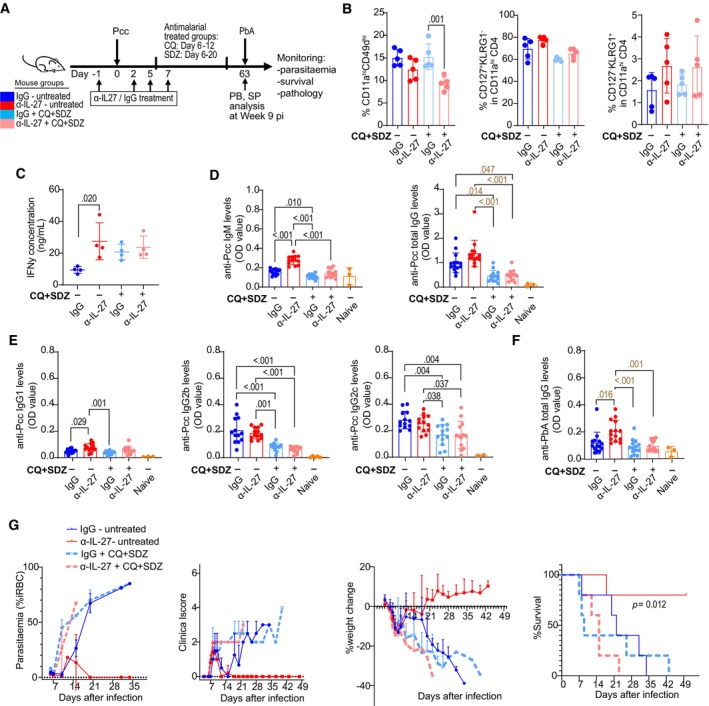
Transient IL‐27 neutralization induces long‐lasting protective immunity against challenge infection with heterologous parasites B6 mice were treated with IgG (blue, light blue) or anti‐IL‐27 mAb (red, pink), infected with Pcc, and treated (light blue, pink) or not (blue, red) with antimalarial drugs. PB profiles were monitored weekly. Splenocytes were examined at 63 dpi (B‐F), or mice were challenged with PbA at 63 days post‐Pcc infection and monitored post‐PbA infection (G).
AExperimental scheme.B–FSplenocytes and serum were examined 63 days after infection. (B) Summary of the proportions of CD11a^hi^CD49d^hi^ cells in splenic CD4^+^ T cells, and the proportions of CD127^+^KLRG1^−^ and CD127^−^KLRG1^+^ cells in CD11a^hi^CD4^+^ T cells (*n* = 4 mice/group). (C) IFN‐γ production as determined by ELISA of splenic CD4^+^ T cells purified and cultured in the presence of dendritic cells and crude Pcc antigens for 48 h (*n* = 4 mice/group). (D‐F) Levels of anti‐Pcc IgM and IgG (D), IgG subclasses (E), and anti‐PbA cross‐reactive IgG (F) in the serum (*n* = 13 mice/group). Pooled data from 3 experiments (4–5 mice/ group) are shown. Serum from uninfected mice (naïve, *n* = 3 mice) was used as negative control.GParasitemia, clinical scores, body weight, and survival were monitored after challenge infection with PbA at day 63 post‐Pcc pi (4 mice/group). Survival was assessed using log‐rank test. Data information: Representative data of 2 independent experiments are shown before (B, C) and after challenge infection (G). Statistical significance assessed by one‐way ANOVA followed by Tukey's multiple comparison test (*P* values (< 0.05) shown in black) or Kruskal–Wallis test with Dunn's *post hoc* tests (*P* values (< 0.05) shown in bown), depending on normality assessment. Error bars represent SD. Source data are available online for this figure.

Finally, we examined whether the immunity induced under IL‐27‐neutralizaion exhibit enhanced recall response against challenge infection. Mice treated with anti‐IL‐27 mAb or control IgG were infected with *P. chabaudi* and treated and not treated with antimalarial drugs without transfer of PbT‐II cells (Fig 7, Appendix Fig S2). Mice were sacrificed 63 days after infection, and splenocytes were examined. Phenotypical analysis did not show significant skewing in lymphocyte composition and CD4^+^ T cell subpopulations between anti‐IL‐27 mAb‐treated and control mice except the reduction of CD11a^hi^CD49d^hi^ CD4^+^ T cells in antimalarial‐treated mice (Figs 7B and EV5B and C). However, production of IFN‐γ by CD4^+^ T cells in response to crude Pcc antigens in antimalarial untreated mice was higher than IgG control, suggesting that memory CD4^+^ T cells were maintained at higher levels in anti‐IL‐27 mAb‐treated mice (Fig 7C). In the nonlymphoid compartment, there was a reduction in the proportion of CD11b^+^Ly6C^hi^ inflammatory monocytes observed in anti‐IL‐27 mAb‐treated antimalarial untreated mice, suggesting that IL‐27 neutralization also has long‐term effect on the non‐lymphoid compartment (Appendix Fig S2C). Levels of anti‐*Plasmodium* IgM and IgG were highest in anti‐IL‐27 mAb‐treated mice without antimalarial therapy (Fig 7D). Among IgG isotypes we observed a significant difference between IgG and anti‐IL‐27 mAb‐treated mice only in IgG1 levels (Fig 7E). IgG cross reactive to *P. berghei* ANKA (PbA) antigens was also higher in anti‐IL‐27 mAb‐treated mice (Fig 7F). We used a previously established re‐challenge infection model with heterologous virulent parasites, PbA (Nakamae *et al*, 2019). Mice were challenged with PbA 63 days after Pcc infection, and the protective ability was evaluated. In anti‐IL‐27 mAb‐treated antimalarial‐untreated mice, the levels of parasitemia reduced and clinical score recovered after 10 days of infection, and mice survived the infection, while parasitemia levels continued to increase, clinical scores continued to increase, and body weights reduced in all other three groups, with mice eventually succumbing to death (Fig 7G). We also evaluated whether the protective ability of Pcc‐primed mice after rechallenge with *Plasmodium* parasites was improved in anti‐IL‐27 mAb‐treated mice. However, these mice barely exhibited parasitemia after rechallenge with homologous Pcc due to strong protective immunity, as reported previously (Achtman *et al*, 2007). Taken together, these results denote that neutralization of IL‐27 during acute infection induced and maintained cellular and humoral immune responses that are protective against recall challenge infection, although chronic infection is required for the maintenance of the protective ability.

## Discussion

This study shows that IL‐27 produced during the acute phase of malaria infection quantitatively and qualitatively modulates the induction and maintenance of memory CD4^+^ T cells. Monitoring of the *Plasmodium* specific‐CD4^+^ T cells, PbT‐II, showed that they exhibit biphasic expansion in the absence of IL‐27. The first phase derived from clonal expansion of activated CD4^+^ T cells resulting in the generation of Th1 and Tfh effector cells as well as memory precursor‐like cells, followed by their contraction. The second expansion phase came between 2 and 3 weeks after infection, dominated by the expansion of Th1‐type memory cells, which remained for a long time thereafter during chronic infection. In a classical model of *P. chabaudi* infection, it was proposed that CD4^+^ T cells undergo biphasic activation; IFN‐γ producing Th1‐type CD4^+^ T cells, followed by antibody‐helper‐type response which were earlier proposed to be Th2 and later described as predominantly Tfh cells (Perez‐Mazliah & Langhorne, 2014; Soon & Haque, 2018). We found that IL‐27 is critical regulator that switches Th1 dominance to Tfh type response during *P. chabaudi* infection. Our study also shows that this second expansion phase of malaria‐specific CD4^+^ T cells is not a direct effect of IL‐27 inhibition, since inhibition with anti‐IL‐27 Ab starting 11 days of infection had no such effect. Rather, inhibition of IL‐27 during the initial 7 days of infection was critical, suggesting that IL‐27 inhibits the generation of memory precursor CD4^+^ T cells that were destined toward differentiation to Th1‐type. Several studies suggest that memory CD4^+^ T cell fate is determined early during infection with Pcc. Memory precursor CD4^+^ T cells are detected among those with effector differentiation during early activation of lymphocytic choriomeningites virus (LCMV) infection and these cells exhibited a gene signature that distinguishes memory precursors (Tcmp) from effectors supporting for an early decision of memory precursors (Harrington *et al*, 2008; Marshall *et al*, 2011; Pepper & Jenkins, 2011; Ciucci *et al*, 2019). In *Plasmodium* infection model, the decision of Th1‐Tfh fate is suggested to be made early after activation by studies on the endogenous TCRαβ sequences in PbT‐II cells and by cell transfer experiments (Soon *et al*, 2020). Our study indicates that the critical time span of the effective IL‐27 neutralization is limited to a short period during early activation of specific CD4^+^ T cells, revealing the critical window of T cell activation for fate determination towards memory generation. IL‐27 is produced mainly by myeloid cells including macrophages and dendritic cells in response to Toll‐like receptor‐dependent signaling as well as immune stimuli such as type I and type II IFNs (Yoshida & Hunter, 2015). IL‐27 may directly modulate precursor memory CD4^+^ T cells as observed in the gene expression patterns during acute infection. Alternatively, early IL‐27 neutralization might have long‐term effects on the environment of T cell response in a way that predominantly induce high levels of Th1‐type memory cells. Further studies will reveal details of molecular mechanisms that underlie the modulation of memory CD4^+^ T cell generation during early activation and their maintenance.

Previous studies of scRNA‐seq analysis on PbT‐II cells suggested that CD4^+^ T cells bifurcate to Th1 and Tfh differentiation at a single‐cell level during acute Pcc infection and these cells transit to memory cells while partially retaining effector phenotypes (Lonnberg *et al*, 2017; Soon *et al*, 2020). However, our study identified PbT‐II cells that do not belong to either Th1 or Tfh on day 7 of Pcc infection and exhibit high Tmem scores. These may include memory precursor cells that differentiate in parallel to effector cells. Th1‐type cells on day 7 also exhibited high Tcmp scores, suggesting their potential as memory precursors (Ciucci *et al*, 2019). While adoptive transfer experiments may resolve the relationship between memory precursor and their progenies, the limited ability of CD4^+^ T‐cell expansion *in vivo* after adoptive transfer hampered these studies. Nonetheless, during chronic infection, majority of the PbT‐II cells exhibited the gene expression signatures of Th1 effector and memory in mice, in which IL‐27 was neutralized during acute infection, suggesting the generation of Th1 memory‐like characteristics is inhibited by IL‐27 produced during acute phase of malaria infection.

Circulating memory CD4^+^ T cells are categorized into CCR7^+^CD62L^hi^CD45RA^−^ central memory (T_CM_) and CCR7^−^CD45RA^−^ effector memory (T_EM_) subsets based on their trafficking potential (Sallusto *et al*, 1999). Studies using Pcc infection model demonstrated that T_EM_ cells are the major memory cells during chronic infection, which produce cytokines and participate in the control of parasitemia, while T_CM_ were induced after short‐term exposure to parasites with infection followed by the drug treatment (Stephens & Langhorne, 2010; Opata *et al*, 2018). We demonstrated that memory type PbT‐II cells induced without IL‐27 were phenotypically and functionally distinct from those induced in IL‐27 sufficient mice. Furthermore, scRNAseq analysis clearly demonstrated that largely nonoverlapping populations of PbT‐II cells are induced in IL‐27‐mAb‐treated and IgG‐treated mice. We identified three populations of PbT‐II cells in IL‐27 neutralized mice: CD127^+^KLRG1^−^, CD127^−^KLRG1^+^, and CD127^−^KLRG1^−^ PbT‐II cells. While both CD127^+^KLRG1^−^ (cluster 1*) and CD127^−^KLRG1^+^ (cluster 1**) populations expressed Th1‐like gene signatures, they are distinct in their gene expression and function. CD127^+^KLRG1^−^ cells expressed higher levels of *Id2*, *Ifng*, Cxcr6, and *Cxcr3*, genes, produced less IFN‐γ and TNF in response to antigenic peptide, and proliferated less *in vivo* when compared with CD127^−^KLRG1^+^ cells. These cells also express *Bcl2* at high level suggesting their memory potential. CD127^−^KLRG1^+^ cells expressed higher levels of *Tbx21*, *Lgals1*, *Klf2*, *Cx3cr1*, *Ccl5* and produce proinflammatory cytokines and proliferate more than CD127^+^KLRG1^−^ cells. Recent studies reported KLRG1^hi^CD127^lo^ effector‐like long‐lived memory CD8^+^ T cells in mice with chronic viral infection that can survive long‐term, have robust effector function, and are effective in clearance of pathogens against rechallenge infection, and also sensitive to checkpoint blockade therapy (Olson *et al*, 2013; Hudson *et al*, 2019; Renkema *et al*, 2020). Not only KLRG1 protein expression but the transcriptional profile of CD127^−^KLRG1^+^ cells is comparable to those CD8^+^ T cells; both express high levels of transcription factors (*Tbx21*, *Klf2*, *Zeb2*), killer cell lectin‐like receptors (*Klrg1*, *Klrd*), and genes related to leukocyte migration (*Cx3cr1*, *Ccl5*, *S1pr1*). These features suggest that a common genetic program may exist among unique CD4^+^ and CD8^+^ T cell subsets, which allow their long‐term survival and effector function during chronic infection, that is critical for the maintenance of protective immunity in the face of ongoing low‐level infection.

Treatment of Pcc‐infected mice with antimalarial drug starting 6 days after infection prevented second wave of PbT‐II expansion induced under IL‐27 neutralizing condition. However, the development of CD127^+^KLRG1^−^ and CD127^−^KLRG1^+^ cells was unaffected by the antimalarial drug treatment implying that their development is independent of their second expansion. Furthermore, both cell types were stably maintained after clearance of *Plasmodium* infection with antimalarial drug treatment starting 21 days after infection, suggesting that they are not short lived. Therefore, IL‐27‐sensitive fate decision of PbT‐II cell differentiation occurs early after Pcc infection and that, once the decision is made, active infection is not required for their maintenance. Among PbT‐II subpopulations on day 28 after infection in anti‐IL‐27 mAb‐treated mice, KLRG1^−^CD127^−^ cells were a minor population but exhibited the highest Tmem signature. Further studies will be required to determine whether these cells can replenish CD127^+^KLRG1^−^ and CD127^−^KLRG1^+^ populations during Pcc chronic infection.

Mice infected with Pcc under IL‐27 neutralization condition were resistant against challenge infection with heterologous parasite, PbA. CD4^+^ T cells from these mice exhibited higher IFN‐γ production in response to *Plasmodium* antigen and higher anti‐*Plasmodium* antibodies in the serum 63 days after infection. However, this protective immune memory was dependent on active infection with Pcc since antimalarial treatment abrogated the maintenance of the protective immunity. This is consistent with the previous study showing that T cells from chronically infected mice protected better than those from mice that were treated with antimalarial drug, implying a critical role for persistent infection in the maintenance of protective memory CD4^+^ T cells (Stephens & Langhorne, 2010). Our study showed that not only cellular immunity but also humoral immunity was improved in chronically infected mice after early IL‐27 neutralization. The antibody response in malaria appears to be dominated by long‐lasting somatically hypermutated high affinity IgM memory B cells, and early secondary response is dominated by IgM responses in both T‐dependent and T‐independent manner as shown in human study and Pcc infection model (Krishnamurty *et al*, 2016). Among Ig isotypes, we showed that anti‐*Plasmodium* IgM levels were affected more than IgG responses by IL‐27 blockade. We speculate that IL‐27 neutralization might have direct effect on B cell compartments in addition to augmenting the maintenance of memory helper T cells.

The immune response in malaria has biphasic roles: prevention of the infection and host damage. The regulatory role of IL‐27 is critical in preventing host pathology by exacerbated Th1‐type immune responses as shown in studies of malaria models using IL‐27 receptor‐deficient mice (Findlay *et al*, 2010; Villegas‐Mendez *et al*, 2013; Yui & Inoue, 2020). Our study identified the critical window of CD4^+^ T cell activation and differentiation during *Plasmodium* infection that is targeted by IL‐27, in which its transient neutralization enhances memory CD4^+^ T cells and improves protection against challenge infection without exacerbated immune responses. IL‐27 levels are increased in individuals infected with *P. falciparum* (Otterdal *et al*, 2020), and this study opens a possibility to improve the host protective immune response while preventing tissue damage due to exacerbated immune responses. Finally, these findings show how the absence of IL‐27 enhances memory during malaria, suggesting potential applications in the development of vaccines and other strategic interventions.

## Materials and Methods

### Reagents and Tools table

**Table:** 

Reagent/Resource	Reference or source	Identifier or catalog number
**Experimental models**		
C57BL/6 mice	SLC	N/A
Mouse: *p28* ^−/−^	Hiroki Yoshida (Saga University)	Kimura *et al* (2016)
Mouse: *EBI3* ^−/−^	Hiroki Yoshida (Saga University)	Igawa *et al* (2009)
Mouse: B6.SJL (CD45.1+)	Available from Jackson Laboratory	
PbT‐II mice	William R Heath (University of Melborne)	Fernandez‐Ruiz *et al* (2017)
*Plasmodium chabaudi chabaudi* AS	Richard Culleton (Ehime University)	
*Plasmodium berghei* ANKA	Masao Yuda (Mie University)	
**Antibodies**		
Anti‐mouse CD4 BV711 (1:500 dilution for surface marker staining panel)	Biolegend	Clone: GK1.5; 100447; RRID: AB_2564586
Anti‐mouse CD4 FITC (1:250 in intracellular cytokine staining panel)	Biolegend	Clone: GK1.5; 100406; RRID: AB_312691
Anti‐mouse CD4 APC/Cy7 (1:500 dilution for PbT‐II cell sorting)	Biolegend	Clone: GK1.5; 100414; RRID: AB_312699
Anti‐mouse CD4 Pacific blue (1:500 dilution for surface marker staining panel, 1:250 in intracellular cytokine staining panel)	Biolegend	Clone: GK1.5; 100428; RRID: AB_493647
Anti‐mouse CD11a FITC (1:500 dilution for surface marker staining panel)	Biolegend	Clone: M17/4; 101106; RRID: AB_312779
Anti‐mouse CD49d BV421 (1:500 dilution for surface marker staining panel, 1:250 in intracellular cytokine staining panel)	BD OptiBuild™	Clone: 9C10 (MFR4.B); 740016; RRID: AB_2739788
Anti‐mouse CD8a APC/Cy7 (1:500 dilution for surface marker staining panel, 1:250 in intracellular cytokine staining panel)	Biolegend	Clone: 53‐6.7; 100714; RRID: AB_312753
Anti‐mouse CD45.1 APC (1:500 dilution for PbT‐II cell sorting)	Biolegend	Clone: A20; 110714; RRID: AB_313503
Anti‐mouse CD45.1 APC/Cy7 (1:500 dilution for surface marker staining panel, 1:250 in intracellular cytokine staining panel)	Biolegend	Clone: A20; 110716; RRID: AB_313505
Anti‐mouse CD45.2 BV605 (1:500 dilution for surface marker staining panel, 1:250 in intracellular cytokine staining panel)	Biolegend	Clone: 104; 109841; RRID: AB_2563485
Anti‐mouse CD3ε BV510 (1:500 dilution for surface marker staining panel)	Biolegend	Clone: 145‐2C11; 100353; RRID: AB_2565879
Anti‐mouse TCR β chain FITC (1:500 dilution for PbT‐II cell sorting)	Biolegend	Clone: H57‐597; 109205; RRID: AB_313428
Anti‐mouse TCR β chain BV510 (1:500 dilution for surface marker staining panel, 1:250 in intracellular cytokine staining panel)	BD Horizon™	Clone: H57‐597; 563221; RRID: AB_2738078
Anti‐mouse CD279 (PD‐1) PE/Cy7 (1:500 dilution for PbT‐II cell sorting)	Biolegend	Clone: 29F.1A12; 135215; RRID: AB_10696422
Anti‐mouse/human KLRG1 (MAFA) APC (1:500 dilution for surface marker staining panel, 1:250 in intracellular cytokine staining panel)	Biolegend	Clone: 2F1/KLRG1; 138412; RRID: AB_10641560
Anti‐mouse CD185 (CXCR5) PE/Cy7 (1:100 dilution for surface marker staining panel, 1:50 in intracellular cytokine staining panel)	Biolegend	Clone: L138D7; 145515; RRID: AB_2562209
Anti‐mouse CD127 (IL‐7Rα) FITC (1:100 dilution for surface marker staining panel, 1:50 in intracellular cytokine staining panel)	Biolegend	Clone: A7R34; 135008; RRID: AB_1937232
Anti‐mouse CD127 (IL‐7Rα) PE (1:500 dilution for surface marker staining panel, 1:250 in intracellular cytokine staining panel)	Biolegend	Clone: A7R34; 135010; RRID: AB_1937251
Anti‐mouse CD127 (IL‐7Rα) BV711 (1:200 dilution for surface marker staining panel, 1:100 in intracellular cytokine staining panel)	Biolegend	Clone: A7R34; 135035; RRID: AB_2564577
Anti‐mouse/human GL7 Antigen PE (1:500 dilution for surface marker staining panel)	Biolegend	Clone: GL7; 144607; RRID: AB_2562925
Anti‐mouse CD186 (CXCR6) PE (1:500 dilution for surface marker staining panel)	Biolegend	Clone: SA051D1; 151103; RRID: AB_2566545
Anti‐mouse CD186 (CXCR6) FITC (1:500 dilution for surface marker staining panel, 1:250 in intracellular cytokine staining panel)	Biolegend	Clone: SA051D1; 151107; RRID: AB_2572144
Anti‐mouse Ki‐67 PE/Cy7 (1:200 dilution)	Biolegend	Clone: 16A8; 652425; RRID: AB_2632693
Anti‐T‐bet PE/Cy7 (1:200 dilution)	Biolegend	Clone: 4B10; 644824; RRID: AB_2561761
Anti‐TCF‐7/TCF‐1 PE (1:200 dilution)	BD Pharmingen™	Clone: S33‐966; 564217; RRID: AB_2687845
Anti‐human/mouse Bcl‐6 PE (1:20 dilution)	Biolegend	Clone: 7D1; 358504; RRID: AB_2562152
Anti‐mouse IFN‐γ Alexa Fluor® (1:200 dilution)	Biolegend	Clone: XMG1.2; 505813; RRID: AB_493312
Anti‐mouse TNF‐α PerCP/Cy5.5 (1:200 dilution)	Biolegend	Clone: MP6‐XT22; 506322; RRID: AB_961434
Anti‐mouse IL‐10 PE (1:200 dilution)	Biolegend	Clone: JES5‐16E3; 505008; RRID: AB_315362
Anti‐mouse IL‐2 PE/Cy7 (1:200 dilution)	Biolegend	Clone: JES6‐5H4; 503832; RRID: AB_2561750
Rat IgG2a, κ Isotype Ctrl FITC (1:500 dilution for surface marker staining, 1:200 in intracellular cytokine staining)	Biolegend	Clone: RTK2758; 400506; RRID: AB_2736919
Armenian Hamster IgG Isotype PE (1:500 dilution for surface marker staining, 1:200 in intracellular cytokine staining)	Biolegend	Clone: HTK888; 400908; RRID: AB_326593
Armenian Hamster IgG Isotype APC (1:500 dilution for surface marker staining, 1:250 in intracellular cytokine staining)	Biolegend	Clone: HTK888; 400912
Mouse IgG1, κ Isotype PE/Cy7 (1:500 dilution for surface marker staining, 1:200 in intracellular cytokine staining)	Biolegend	Clone: MOPC‐21; 400126; RRID: AB_326448
Rat IgG2a, κ Isotype BV421 (1:500 dilution)	BD Horizon	Clone: R35‐95; 562602; RRID: AB_11153860
Rat IgG1, κ Isotype PerCP‐Cy5.5 (1:500 dilution for surface marker staining, 1:250 in intracellular cytokine staining)	Biolegend	Clone: RTK2071; 400425
Purified anti‐mouse CD16/32 (1:500 dilution)	Biolegend	Clone: 93; 101302; RRID: AB_312801
InVivoMAb anti‐mouse IL‐27 p28 (250 ug per mouse)	Bio X Cell	Clone: MM27.7B1; BE0326; RRID: AB_2819053
IgG from rat serum (250 ug per mouse)	Sigma‐Aldrich	I4131‐50MG; RRID: AB_1163627
TotalSeq™‐C0250 anti‐mouse/human KLRG1 (MAFA; 1:500 dilution)	Biolegend	Clone: 2F1/KLRG1; 138433; RRID: AB_2800649
TotalSeq™‐C0198 anti‐mouse CD127 (IL‐7Rα; 1:500 dilution)	Biolegend	Clone: A7R34; 135047; RRID: AB_2819874
TotalSeq™‐C0078 anti‐mouse CD49d (1:500 dilution)	Biolegend	Clone: R1‐2; 103633; RRID: AB_2860605
TotalSeq™‐C0238 Rat IgG2a (1:500 dilution)	Biolegend	Clone: RTK2758; 400577
**Chemicals, enzymes and other reagents**		
PbT‐II peptide	SIGMA Genosys	Custom order
7‐amino‐actinomycin D (7AAD)	Cayan Chemical Company	Cat # 11397
CD11c MicroBeads UltraPure, mouse	Miltenyi Biotech	Cat # 130‐125‐835
Chloroquine diphosphate	Sigma	Cat# C6628
Sulfadiazine	Sigma	Cat# S6387
Phorbol 12‐myristate 13‐acetate (PMA)	Cayan Chemical Company	Cat # 10008014
Ionomycin	Cayan Chemical Company	Cat # 10004974
Brefeldin A	Cayan Chemical Company	Cat # 11861
Crude Pcc antigen	Prepared in Lab	N/A
Crude PbA antigen	Prepared in Lab	N/A
**Software**		
*Include version where applicable*		
FlowJo 10	TreeStar	RRID:SCR_008520
Prism 9	GraphPad https://www.graphpad.com	RRID:SCR_002798
R version 4.2.1	R Project https://www.r‐project.org	
Cell Ranger	10X Genomics	
Seurat 4.2.0	https://satijalab.org/seurat/	
Metascape 3.5	http://metascape.org Zhou *et al* (2019)	
**Other**		
CD4^+^ T cell Isolation Kit, mouse	Miltenyi Biotech	Cat # 130‐104‐454
Foxp3/Transcription Factor Staining Buffer Set	eBioscience	Cat # 00‐5523‐00
Fixation/Permeablization Kit	BD Cytofix/Cytoperm™	Cat # 554714; RRID: AB_2869008
Chromium Next GEM Single Cell 5' v2 Reagent kits (Dual Index)	10X Genomics	1000265
Chromium 5′ Feature Barcode Kit, 16 rxns	10X Genomics	1000256
Chromium NEX GEM Chip K Single Cell Kit, 16 rxns	10X Genomics	1000287
Chromium Dual Index Kit TT Set A, 96 rxns	10X Genomics	1000215
Chromium Dual Index Kit TN Set A, 96 rxns	10X Genomics	1000250
SPRIselect	Beckman Coulter Genomics	B23317
High Sensitivity DNA Analysis Kits	Agilent Technologies	Cat # 5067‐4626

### Methods and Protocols

#### Mice

*Il‐27* ^−/−^ and *Ebi3* ^−/−^ mice were described previously (Igawa *et al*, 2009; Kimura *et al*, 2016). PbT‐II mice (Fernandez‐Ruiz *et al*, 2017) were generously provided by Dr. W.R. Heath and crossed with B6.SJL‐Ptprc mice (CD45.1^+^) to generate CD45.1^+^ PbT‐II mice. C57BL/6 (B6) mice were purchased from SLC (Shizuoka, Japan), and B6.SJL mice were bred in‐house and maintained at controlled pathogen‐free conditions in the Laboratory Animal Center for Animal Research at Nagasaki University. Mice were used at 8–12 weeks of age and were age and gender matched for each experiment. All mice were maintained in the Laboratory Animal Center for Animal Research at Nagasaki University. Animal experiments were performed with approval from the Institutional Animal Care and Use Committee of Nagasaki University (#2003091602) and were conducted according to the guidelines for Animal Experimentation at Nagasaki University.

#### *Plasmodium* parasites and antimalarial drug treatment

*Plasmodium chabaudi chabaudi* AS (Pcc) is a cloned parasite, which is originally from Dr. Richard Carter and David Walliker's rodent malaria parasite collection at the University of Edinburgh (Stephens *et al*, 2012), and was obtained from Dr. R Culleton (Ehime University, Ehime, Japan). *P. berghei* ANKA (PbA) was originally obtained from Dr. RE Sinden (Imperial College London, UK) and kindly provided by Dr. M Yuda (Mie University, Mie, Japan). Cryopreserved Pcc and PbA parasites were passaged through B6 mice before use in infecting experimental mice. Mice were infected with infected red blood cells (iRBC; 5 × 10^4^) intraperitoneally (i.p.), and parasitemia was monitored from 4 days post‐infection (dpi) by observing thin blood smears stained with a diff‐quick procedure (Sysmex, Kobe, Japan).

For antimalarial drug treatment, chloroquine (10 μg/g body weight; Sigma) was administered i.p. from 6 or 21 dpi for 7 days, and sulfadiazine in drinking water (30 mg/l; Sigma) starting on the same day for 14 days unless otherwise specified in the experimental schemes.

#### Neutralization of IL‐27 *in vivo*

For IL‐27 neutralization, anti‐IL‐27p28 mAb (250 ug/mouse; Clone: MM27.7B1; Bio X Cell) was injected i.p. 1 day before and 2, 5, 7 days after Pcc infection, unless otherwise specified in the experimental schemes. Rat IgG (250 μg/mouse; Sigma‐Aldrich) was administered to control groups following the same dose intervals as experimental groups.

#### Flow cytometry

Peripheral blood (PB) was collected from the tail vein of mice during monitoring, and by cardiac puncture when mice were sacrificed. Single‐cell suspensions were prepared from spleen, and red blood cells in PB and spleen were lysed using Gey's solution. After incubation with anti‐Fcγ receptor mAb (purified antimouse CD16/32; Clone: 93; Biolegend), cells (3 × 10^6^) were stained for surface markers for 30 min at 4°C. Isotype control antibodies were used to assess staining of specific markers. Antibodies used for multiple panels of flow cytometry analysis are listed in the Reagents and Tools table. 7‐amino‐actinomycin D (7AAD; Cayan Chemical Company) was used to excluded dead cells from analysis.

For intracellular staining of transcription factors (TF), splenocytes were treated with anti‐Fcγ receptor mAb (Clone: 93; Biolegend), stained for surface markers, fixed, and permeabilized using a Foxp3/Transcription Factor Staining Buffer set (eBioscience) following the manufacturer's instructions. The TF antibodies used were antimouse Ki‐67 PE/Cy7 (Clone: 16A8; Biolegend), anti‐T‐bet PE/Cy7 (Clone: 4B10; Biolegend), anti‐TCF‐7/TCF‐1 PE (Clone: S33‐966; BD Pharmingen™), and antihuman/mouse Bcl‐6 PE (Clone: 7D1; Biolegend). Fixed/permeabilized cells were incubated with antibodies specific for T‐bet and TCF1 in permeabilization buffer for 1 h at 4°C and for Bcl6 and Ki67 for 1 h at room temperature.

For analysis of cytokine production, cells were cultured in R10 medium (RPMI 1640 with 10% fetal calf serum (FCS), 2 mM glutamine, penicillin/streptomycin, 2‐mercaptoethanol (5 × 10^−5^ M), 0.1 mM nonessential amino acids, and 1 mM sodium pyruvate) in the presence of brefeldin A (10 μg/ml; Cayan Chemical Company). Splenocytes (2 × 10^6^) were stimulated with PMA (25 ng/ml; Cayan Chemical Company) and ionomycin (1 μg/ml; Cayan Chemical Company) for 4 h at 37°C in a humidified atmosphere of 5% CO_2_. For stimulation with antigenic peptide, DCs were prepared from splenocytes of naïve B6 using anti‐CD11c magnetic‐activated cell sorting (MACS) microbeads (Miltenyi Biotech) and AutoMACS cell separator (Miltenyi Biotech) and were pulsed with PbT‐II peptide (2 μM; SIGMA Genosys; Enders *et al*, 2021) for 1 h. The peptide was custom synthesized by SIGMA Genosys (Sigma‐Aldrich Japan, Tokyo, Japan). CD4^+^ T cells were purified from splenocytes by magnetic separation using anti‐CD4 IMag beads (BD Biosciences) and were cultured with peptide‐pulsed DCs for 4 h at 37°C in a humidified atmosphere of 5% CO_2._ After culture, cells were surface stained, fixed for 20 min using Cytofix/Cytoperm™ (BD Biosciences), and stained with anti‐cytokine mAbs in permeabilization buffer. Analysis of the stained cells was performed using a LSRFortessa X‐20 cell analyzer (BD Biosciences) and FlowJo software.

#### scRNA‐seq sample and library generation

B6 mice were transferred with PbT‐II cells, infected with Pcc, and were treated with control IgG or anti‐IL‐27 mAb between −1 and 7 days after infection with 1 mouse for each time point. Spleen cells were prepared 7 and 28 days after infection for IgG control and 7, 14, and 28 days after infection for anti‐IL‐27 mAb‐treated groups. CD4^+^ T cells were prepared from spleen using mouse CD4^+^ T cell Isolation Kit (Miltenyi Biotech) and AutoMACS cell separator (Miltenyi Biotech) according to manufacturer's instructions. Enriched CD4^+^ T cells, which included PbT‐II cells, were incubated with a panel of TotalSeq™ ‐C antibodies (BioLegend) and surface antibody markers (BioLegend) in PBS + 1% BSA: TotalSeq™‐C0250 anti‐mouse/human KLRG1 (MAFA; Clone: 2F1/KLRG1), TotalSeq™‐C0198 anti‐mouse CD127 (IL‐7Rα; Clone: A7R34), TotalSeq™‐C0078 anti‐mouse CD49d (Clone: R1‐2), and TotalSeq™‐C0238 Rat IgG2a (Clone: RTK2758), as well as antimouse CD4 APC/Cy7 (Clone: GK1.5), anti‐mouse TCR β chain FITC (Clone: H57‐597), anti‐mouse CD45.1 APC (Clone: A20), and 7AAD (Cayan Chemical Company). Stained CD4^+^ T cells were washed using the recommended Cell Wash Protocol 1 in preparation for 10X Single Cell RNA sequencing (Chromium Next GEM Single Cell 5′ v2 Reagent kits (Dual Index); 10X Genomics). PbT‐II cells (CD4^+^CD45.1^+^TCR^+^) were sorted using FACSAria II cell sorter (BD Biosciences). FACS‐sorted cells were collected in PBS + 1% BSA after washing, and resulted in > 99% purity and > 98% viability in all samples.

Single‐cell RNA‐seq libraries were prepared using the Chromium Next GEM Single Cell 5′ Reagent Kits v2 (Dual Index; 10X Genomics), SPRI reagent (Beckman Coulter Genomics), and High Sensitivity DNA Analysis Kits (Agilent Technologies), according to the manufacturer's instructions. Briefly, sorted PbT‐II cells suspended in PBS + 1% BSA (900–1,200 cells/μl). For GEM generation and barcoding, cells were mixed with master mix and loaded with the gel beads and run on the Chromium Controller (10X). After reverse transcription and barcoding in droplets, cDNA was purified from GEMs, amplified, and were used for generating the gene expression libraries and cell surface protein libraries. Libraries were quantified using Bioanalyzer High Sensitivity Chip (Agilent). Gene expression libraries and cell surface protein libraries were pooled for library construction, and sequencing was performed using MGISeq‐2000RS at Research Institute for Microbial Disease, Osaka University (Osaka, Japan).

#### Cell culture

CD4^+^ T cells were purified (> 95%) from the spleen using anti‐CD4 IMag (Miltenyi Biotech) according to the manufacturer's instructions. Dendritic cells were prepared from spleen of uninfected B6 mice using anti‐CD11c magnetic activated cell sorting (MACS) microbeads (Miltenyi Biotech) and autoMACS (Miltenyi Biotech) following manufacturer's instructions. CD4^+^ T cells (2 × 10^5^) were suspended in culture medium and cultured in a 96‐well round‐bottomed plate in the presence of dendritic cells (1 × 10^4^) with or without Pcc crude antigen (freeze–thaw lysate lysate of 5 × 10^6^ infected RBCs) for 48 h at 37°C in a humidified atmosphere of 5% CO_2_.

#### ELISA

Serum antibody levels against crude Pcc or PbA antigen (prepared in the lab) were determined using ELISA as previously described (Nakamae *et al*, 2019). Briefly, ELISA plates were coated with crude Pcc or PbA freeze–thaw lysate (5 × 10^6^ infected RBC per well) in PBS overnight and washed thrice with PBS‐T washing buffer (PBS with 0.2% Tween 20) before blocking with PBS with 10% FCS and 0.2% Tween 20 for 1 h. After washing, serum samples (1:1,000 dilution, 50 μl) were added, incubated at 4°C overnight, washed, and antimouse IgG HRP antibody (Southern Biotech; 1:1,000 dilution) was added. After another washing, TMB substrate solution (100 μl; Thermo Fisher Scientific) was added in each well, developed for at most 10 min, before adding stop solution (10% phosphoric acid, 50uL), and read at 595/450 nm dual wavelength using an iMark Microplate Absorbance Reader (Bio‐Rad, Hercules, CA, USA). For the detection of IgM and IgG isotypes, biotin‐conjugated rabbit antimouse IgG2c (Bethyl Laboratories, Montgomery, TX, USA), IgM, IgG1 or IgG2b (ZyMED, San Francisco, CA, USA) antibodies were added, incubated for 1 h, washed, incubated with alkaline phosphatase‐conjugated streptavidin (Jackson ImmunoResearch, West Grove, PA, USA), washed, and NPP (4‐Nitrophenyl phosphate disodium salt hexahydrate) solution was added. Plates were read at 405 nm absorbance.

The levels of IFN‐γ in culture supernatant were determined with sandwich ELISA using anti‐IFN‐γ (R4‐6A2) and biotin‐anti‐IFN‐γ (XMG1.2) mAbs as described previously (Nakamae *et al*, 2019).

#### Statistical analysis

All statistical analyses were performed using Graphpad Prism 9 (https://www.graphpad.com; GraphPad). Flow cytometry data were analyzed using Flowjo software (TreeStar). Data were assessed for normality using the Shapiro–Wilk test. For determining statistical significance of results, two‐tailed unpaired Student's *t*‐tests were used for pairwise comparisons for normally distributed data (parametric), while Mann–Whitney *U* test (nonparametric) was used for non‐normally distributed data. For comparing more than two groups, one‐way analysis of variance (ANOVA) with Tukey HSD *post hoc* tests (parametric) or Kruskal–Wallis test with Dunn's *post hoc* tests (non‐parametric) were performed where applicable. Survival curves were assessed using Log‐rank (Mantel‐Cox) test. Comparisons with *P* values < 0.05 were considered statistically significant.

#### scRNA‐seq and CITE‐seq data analysis

Sequence of the pooled libraries was mapped to the reference mouse genome using Cell Ranger 6.0.0. at Next‐Generation Sequencing Core Facility, Research Institute for Microbial Disease, Osaka University (Osaka, Japan). The fastq files of five PbT‐II samples (IgG‐treated Days 7 and 28, and anti‐IL‐27‐treated Days 7, 14, and 28) were processed using the Cell Ranger v.6.0.0 (10× Genomics) count pipeline against the mm10‐2020‐A mouse reference sequence. We used Seurat v.4.2.0 (Stuart *et al*, 2019) for detailed analysis. Cells with < 1,500, < 1,000, < 1,500, < 1,000, and < 1,000 or > 5,000, > 4,000, > 5,000, > 4,000, and > 3,000 detected genes were also excluded for each dataset (IgG‐treated Days 7 and 28, and anti‐IL‐27‐treated Days 7, 14, and 28), respectively. The RNA data was subsequently normalized using the “NormalizeData” function, searched for 5,000 highly variable genes using the “FindVariableFeatures” function, and the Protein data was subsequently normalized using the “NormalizeData” and “ScaleData” function. For each analysis, we integrated groups using the “FindIntegrationAnchors” and “IntegrateData” functions and visualized the integrated data using UMAP (Becht *et al*, 2018) with adjustments for resolution parameters to limit cluster numbers depending on the analysis (i.e., 0.15 resolution for the comparison of day 7 IgG and anti‐IL‐27 mAb‐treated groups in Fig 4A, 0.25 resolution for the comparison of day 28 IgG and anti‐IL‐27 mAb‐treated groups in Fig 5A and 0.3 resolution for the analysis of PbT‐II in anti‐IL‐27 mAb‐treated mice shown in Fig 5F). The cell cycle phase of each cell in the datasets was predicted using the CellCycleScoring function with genes converted from “cc.genes$s.gens” and “cc.genes$g2m.genes”. The Th1, Tfh, Tmem, and Tcmp signatures are defined by the average expression of Th1, Tfh, Tmem, and Tcmp genes based on Ciucci *et al* (2019) for each cell. To identify genes that define each cluster, differential expression analysis was performed using the FindConservedMarkers function to identify genes that are conserved across independent samples (different treatments or timepoints) in each cluster. Cluster gene expression patterns were compared for the different cluster analyses done (Figs 4A and 5A and F), and cluster labels were modified to reflect the observed similarities in gene expression patterns accordingly. Moreover, FindMarkers function was also performed to compare 2 cell subsets of interest. Gene ontology analysis of the differentially expressed genes between cell subsets was performed using Metascape (http://metascape.org; Zhou *et al*, 2019).

We analyzed our PbT‐II data on days 7 and 28 in anti‐IL‐27 mAb‐treated mouse together with IgG‐treated counterparts, which were integrated as described above, and the cluster distributions were compared. A reference‐based approach of analysis was also carried out by mapping the scRNAseq data to a reference CD4^+^ T cell atlas (Andreatta *et al*, 2022) using the ProjecTILs R package (version 3.0.0) for classifying and comparing the distribution of T cell states. We compared our PbT‐II from anti‐IL‐27 mAb‐treated and IgG control on days 7 and 28, by first downsizing each dataset to 1,000 single‐cell data points per sample before projecting onto the reference atlas using default parameters.

## Author contributions

**Maria Lourdes Macalinao:** Conceptualization; data curation; software; formal analysis; validation; investigation; visualization; methodology; writing – original draft; writing – review and editing. **Shin‐Ichi Inoue:** Supervision; investigation; methodology; writing – original draft. **Sanjaadorj Tsogtsaikhan:** Investigation. **Hirotaka Matsumoto:** Software; formal analysis. **Ganchimeg Bayarsaikhan:** Investigation. **Jiun‐Yu Jian:** Investigation. **Kazumi Kimura:** Resources. **Yoshiaki Yasumizu:** Formal analysis. **Tsuyoshi Inoue:** Resources. **Hiroki Yoshida:** Resources. **Julius Hafalla:** Supervision; writing – original draft; writing – review and editing. **Daisuke Kimura:** Conceptualization; investigation; methodology. **Katsuyuki Yui:** Conceptualization; data curation; formal analysis; supervision; funding acquisition; validation; methodology; writing – original draft; project administration; writing – review and editing.

## Disclosure and competing interests statement

The authors declare that they have no conflict of interest.

## Supporting information



Appendix S1

Expanded View Figures PDF

PDF+

Source Data for Figure 1

Source Data for Figure 2

Source Data for Figure 3

Source Data for Figure 4

Source Data for Figure 5

Source Data for Figure 6

Source Data for Figure 7

## Data Availability

The datasets produced in this study are available in the following database: Single‐cell RNA‐seq data: Deposited at the Gene Expression Omnibus (GEO) database under the accession number GSE225556 (http://www.ncbi.nlm.nih.gov/geo/query/acc.cgi?acc=GSE225556).
