# Supplementary material for: IL‐27 produced during acute malaria infection regulates Plasmodium‐specific memory CD4 + T cells
Source: EMBO Mol Med. 2023 Oct 19;15(12):e17713. doi: 10.15252/emmm.202317713 (PMC10701605; doi:10.15252/emmm.202317713)
Supplement: Supplementary file 1 — Appendix S1 [file EMMM-15-e17713-s002.pdf]

Appendix

IL-27 produced during acute malaria infection regulates *Plasmodium*-specific memory $CD4^+$ T cells

<u>Content</u>

Appendix Figure S1. IL-27 neutralization affects the differentiation of PbT-II cells in peripheral blood during chronic infection ……………………..…………………….……..**1**

Appendix Figure S2. IL-27 neutralization affects the distribution of inflammatory monocytes during the memory phase after Pcc infection…………..………………….……..**1**

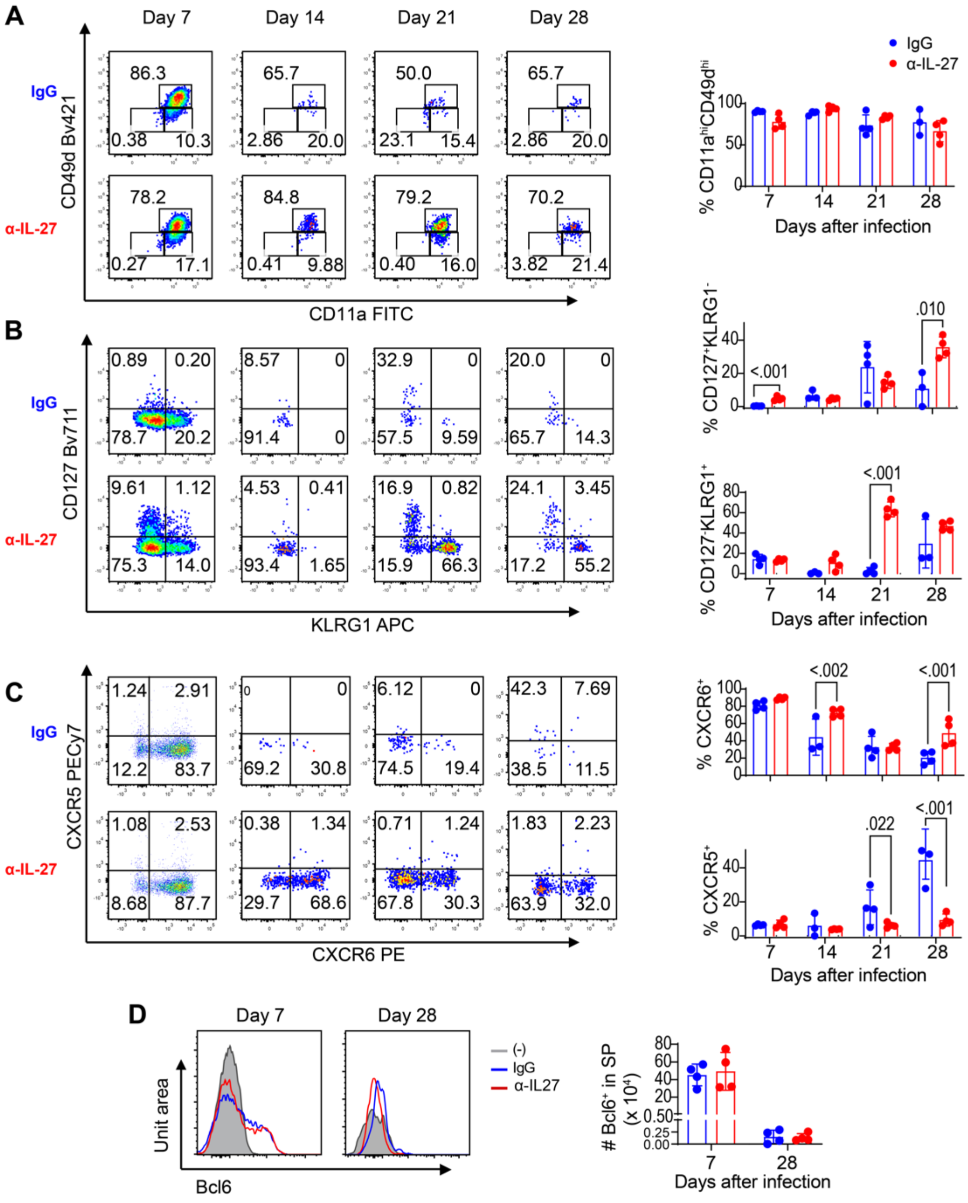

Appendix Figure S1. IL-27 neutralization affects the differentiation of PbT-II profile in peripheral blood. Related to Figure 2.

B6 mice were transferred with PbT-II cells and treated with either control (IgG, blue) or anti-IL-27 mAb (α-IL-27, red) between -1 and 7 days post-Pcc infection. PbT-II cells in PB were analyzed by flow cytometry at 7, 14, 21, and 28 dpi.

(A-C) Representative flow cytometry plots (left) depicting cell surface marker expression of CD11a/CD49d (A), CD127/KLRG1 (B), and CXCR5/CXCR6 (C) in PbT-II cells (IgG-treated mice, n=4, 3, 4, 3, and anti-IL-27 mAb-treated mice, n=4, 4, 4, 4 mice for days 7, 14, 21, 28 days pi, respectively), and summary of the frequencies of PbT-II subpopulations indicated (right). Representative data of 2, 2, 3 and 3 independent experiments for day 7, 14, 21, and 28 pi, respectively.

(D) Representative histograms (left) of Bcl6 expression gated on PbT-II cells in control (blue) and α-IL-27 (red) mouse groups at days 7 and 28 pi (n = 4 mice/group) and their isotype control (gray), with corresponding summary graphs of total numbers of Bcl6^{+} PbT-II cells (right).

Data information: Numbers in flow cytometry profiles indicate the proportions (%) of PbT-II cells within each area. Statistical significance was assessed by Student's *t* test or Mann-Whitney *U* test, depending on normality assessment. *p* values (< 0.05) are shown. Error bars represent SD.

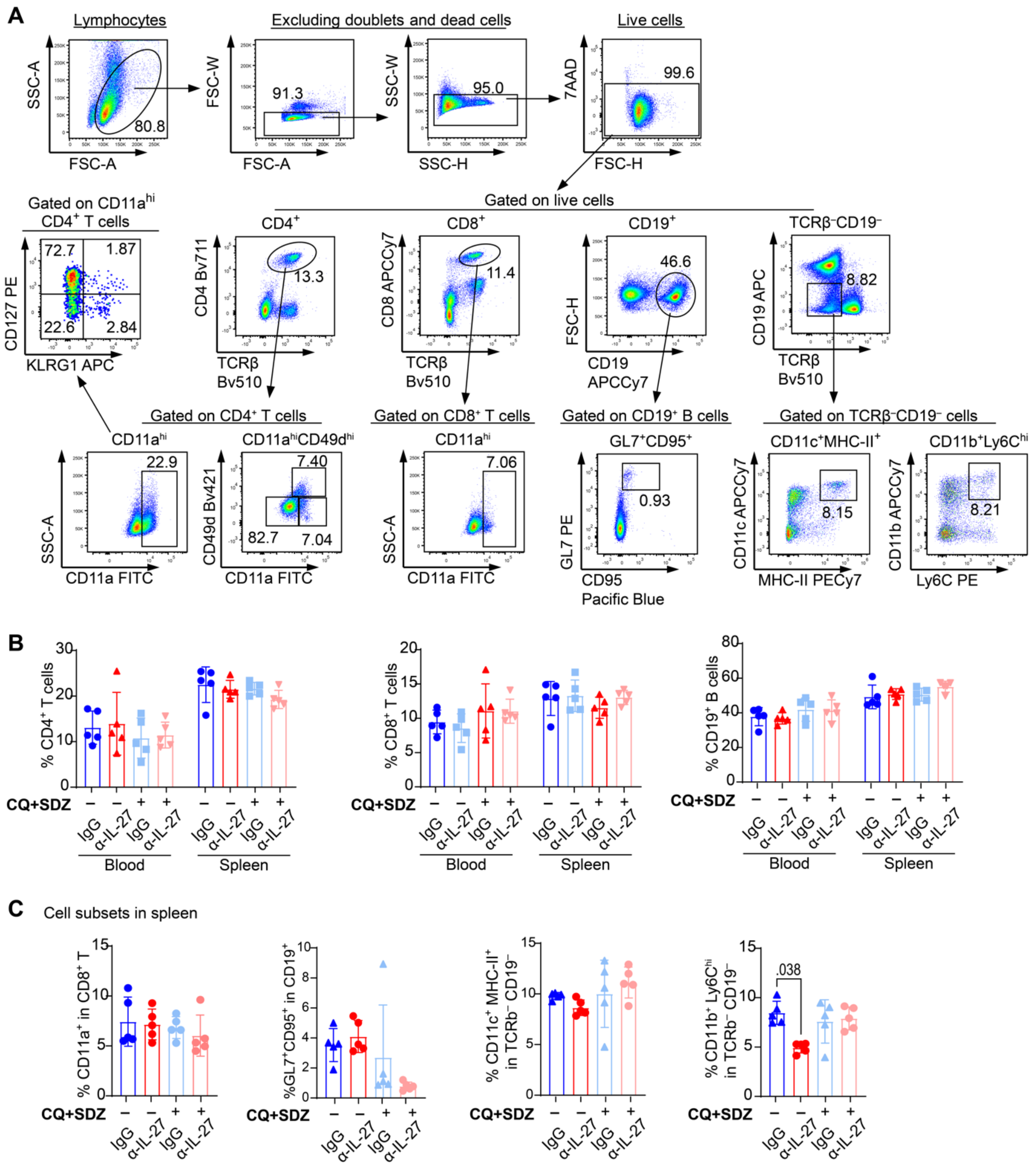

Appendix Figure S2. IL-27 neutralization affects the distribution of inflammatory monocytes during the memory phase after Pcc infection. Related to Figure 7.

B6 mice were prepared in 4 groups: IgG and no drug (blue), anti-IL-27 mAb and no drug (red), IgG and antimalarial drugs (light blue), and anti-IL-27 mAb and antimalarial drugs (pink) as shown in Fig 7 (n = 5 biological replicates per treatment group). Cells in PB and splenocytes were stained and analyzed using flow cytometry 63 days after infection with Pcc.

(A) Gating strategy for determining the distribution of $CD4^+$ T cells, $CD8^+$ T cells, B cells, activated lymphocytes, dendritic cells, and inflammatory monocytes.

(B) Proportions of $CD4^+$ T cells, $CD8^+$ T cells and $CD19^+$ B cells in PB and spleen.

(C) Proportions of activated $CD8^+$ T cells ($CD11a^+$), and populations within $TCRb^-CD19^-$ (non-T and B cell), including germinal center B cells ($GL7^+CD95^+$), conventional dendritic cells ($CD11c^+MHC^+$), and inflammatory monocytes ($CD11c^+Ly6C^{hi}$) in spleen.

Data information: Numbers in flow cytometry profiles indicate the proportions (%) of gated cells within each area. Statistical significance assessed by one-way ANOVA followed by Tukey's multiple comparison test or Kruskal-Wallis test with Dunn's *post.*
