# Supplementary material for: IL‐27 produced during acute malaria infection regulates Plasmodium‐specific memory CD4 + T cells
Source: EMBO Mol Med. 2023 Oct 19;15(12):e17713. doi: 10.15252/emmm.202317713 (PMC10701605; doi:10.15252/emmm.202317713)
Supplement: Supplementary file 2 — Expanded View Figures PDF [file EMMM-15-e17713-s011.pdf]

Expanded View Figures

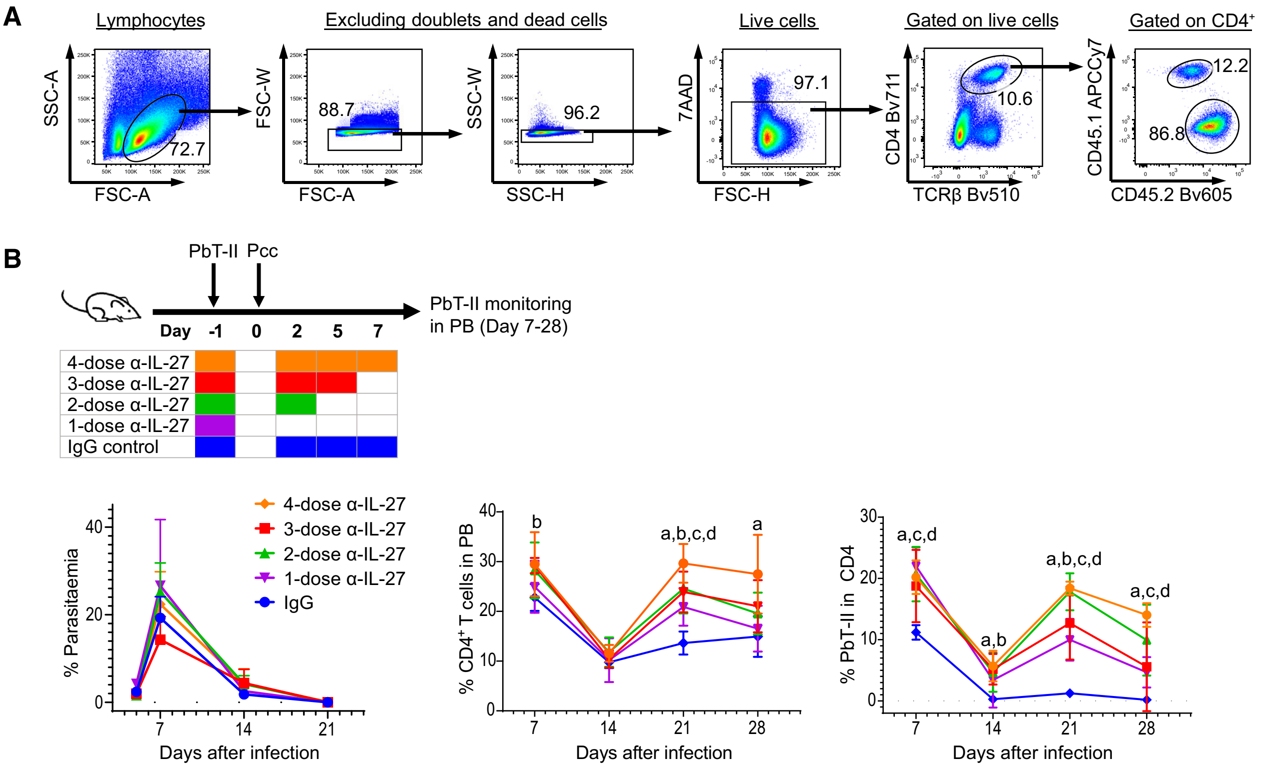

Figure EV1. IL-27 acts on PbT-II cells during the early phase of infection. Related to Fig 1.

A Gating strategy for flow cytometry analysis of PbT-II cells. Samples were stained for CD4, TCRβ, CD45.1, and CD45.2 to identify PbT-II cells. APCCy7 anti-CD45.1 and Bv605 anti-CD45.2 mAbs for the congenic markers and Bv510 anti-TCRβ mAb were maintained in all panels. For CD4 staining, Bv711 anti-CD4 mAb was used in Figs 1, 2A and B, and 6E, and EV2A and B; Pacific Blue-anti-CD4 mAb for Figs 2C and D, and 3, and 6E, and EV2C, and FITC anti-CD4 mAb for Figs 2E and 6F and EV2D.

B B6 mice transferred with PbT-II cells were administered with anti-IL27 mAb in 4 different conditions or IgG control at timepoints indicated and were infected with Pcc (n = 4 mice/group). Parasitemia levels, proportion of $CD4^+$ T cells, and proportions of PbT-II cells in $CD4^+$ T cells in PB were monitored. Representative data of two independent experiments are shown.

Data information: Statistical significance was assessed by one-way ANOVA followed by Tukey's multiple comparison test in (B) and by Student's t test in (C). P values (< 0.05) are shown. Small letters in (B) indicate significant differences compared to IgG control (a = 4-dose, b = 3-dose, c = 2-dose, d = 1-dose treatment), and P-values are: day 7, b = 0.010; day 21, a = < 0.001, b = 0.015, c = 0.010, d = 0.022; day 28, a = 0.035 (middle graph); day 7, a = 0.031, c = 0.022, d = 0.009; day 14, a = 0.023, b = 0.029; day 21, a = < 0.001, b = 0.002, c = < 0.001, d = 0.018; day 28, a = 0.001, c = 0.044, d = 0.036 (right graph). Error bars represent SD.

Figure EV2. IL-27 affects development of PbT-II cells during the transition from acute to chronic malaria infection.

Spleen cells were prepared from mice on day 7 (A, C) and 28 (B, D) pi and were stained with CD11a and CD49d, along with other markers described in Fig 2 (n = 4 mice/group).

A, B Representative CD11a/CD49d profiles of $CD45.1^+CD45.2^-$ (PbT-II) and $CD45.1^-CD45.2^+$ (host $CD4^+$ T) cells (left) and flow cytometry profiles of PbT-II subpopulations of $CD11a^{hi}CD49d^{hi}$ and $CD11a^{hi}CD49d^{lo}$ PbT-II subpopulations are shown (right).

C, D Corresponding summary frequencies of $CD11a^{hi}CD49d^{hi}$ and $CD11a^{hi}CD49d^{lo}$ PbT-II subpopulations.

Data information: Numbers in flow cytometry profiles indicate PbT-II proportions (%) within each area. Statistical significance was assessed by Student's t test (P values (< 0.05) shown in black) or Mann–Whitney U test (P values (< 0.05) shown in brown) per time point, depending on normality assessment. Error bars represent SD.

 © 2023 The Authors

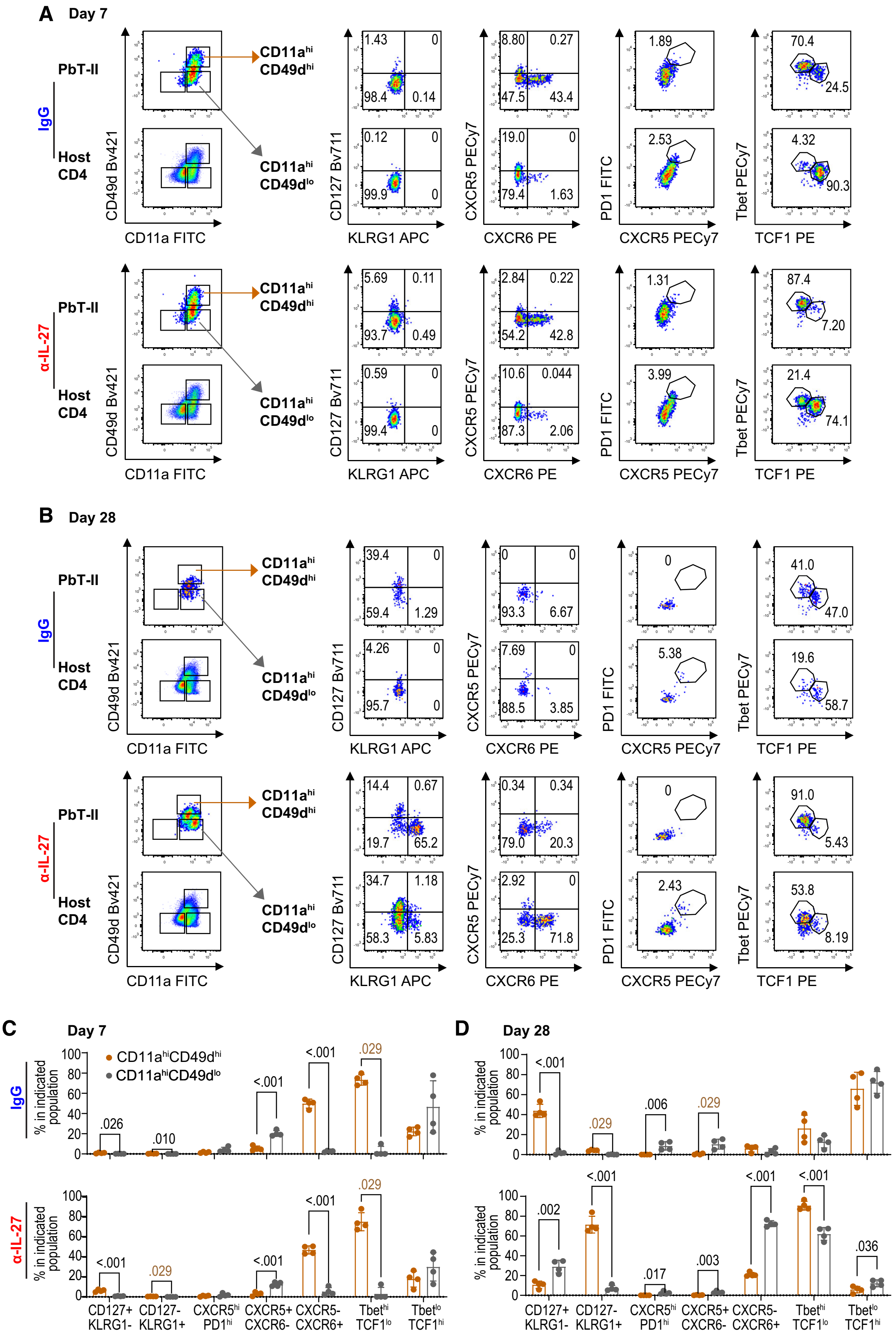
A
Day 7
IgG
α-IL-27
PbT-II
Host CD4
CD49d Bv421
CD11a FITC
CD127 Bv711
KLRG1 APC
CXCR5 PECy7
CXCR6 PE
PD1 FITC
CXCR5 PECy7
Tbet PECy7
TCF1 PE
B
Day 28
C
Day 7
D
Day 28
% in indicated population
CD127+ KLRG1-
CD127- KLRG1+
CXCR5hi PD1hi
CXCR5+ CXCR6-
CXCR5- CXCR6+
Tbethi TCF1lo
Tbetlo TCF1hi

Figure EV2.

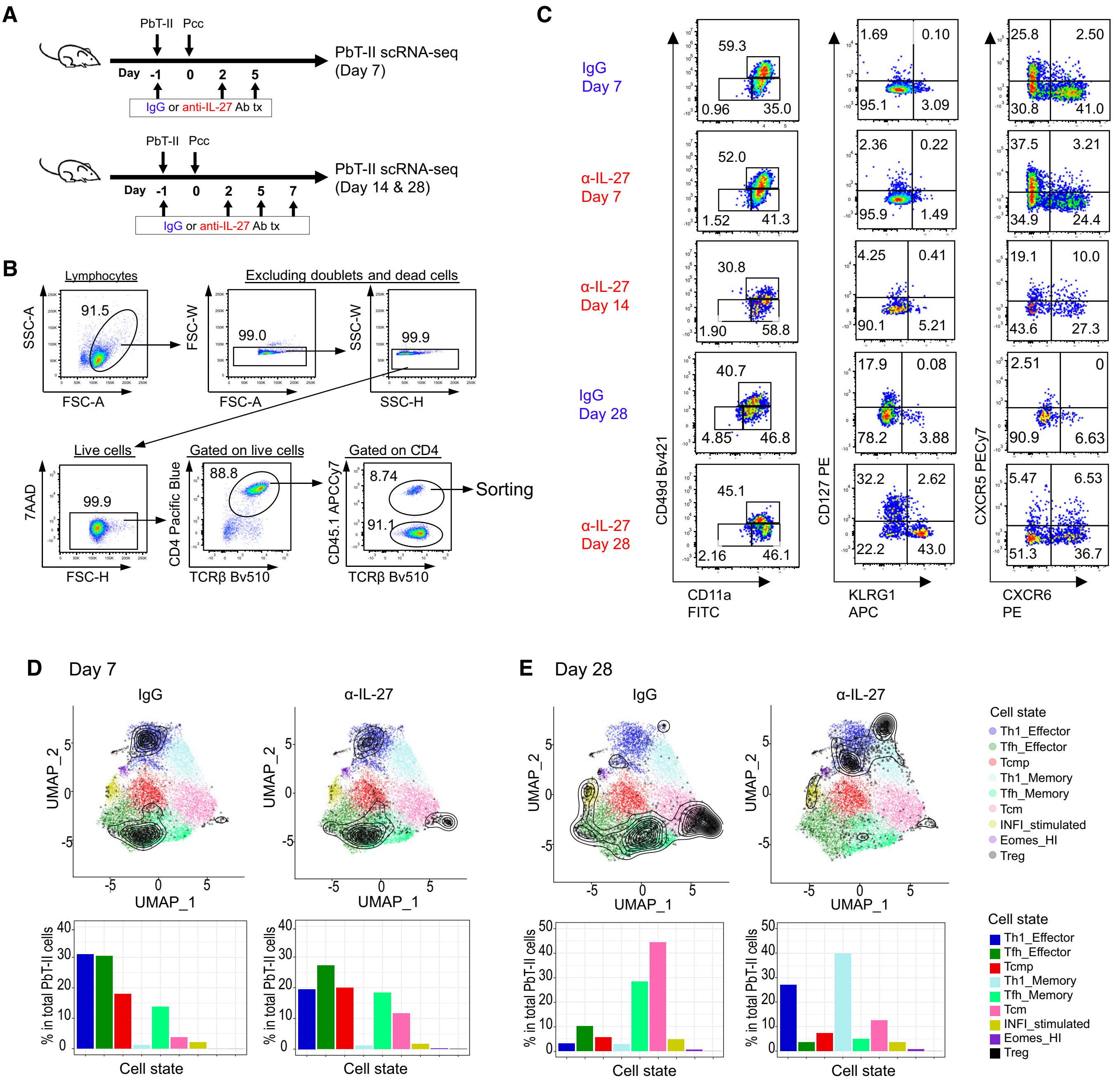

Figure EV3. scRNAseq analysis shows Th1-biased CD4$^+$ T cell development during malaria chronic infection. Related to Figs 4 and 5.

B6 mice were transferred with PbT-II cells, treated with IgG or anti-IL-27 mAb on day −1, 2 and 5 for day 7 analysis, while mice were treated with anti-IL-27 mAb on day −1, 2, 5, and 7 for day 14 and 28 analysis (n = 1 biological replicate per timepoint). PbT-II cells were purified and subjected to single-cell RNA sequencing (scRNA-seq) and CITE-seq analysis. The ProjecTILs algorithm (Andreatta et al, 2021) was used to analyze CD4$^+$ T cell states of PbT-II cells based on a published reference atlas (Andreatta et al, 2022).

A Experimental scheme.

B Gating strategy for the sorting of PbT-II cells for the scRNA-seq experiments: Spleen cells were stained for CD4, TCRβ, and CD45.1 to distinguish PbT-II cells and for TotalSeq IgG2a, CD127, KLRG1, and CD49d for CITE-seq analysis.

C Flow cytometry profiles for each PbT-II sample analyzed for single-cell transcriptomics.

D, E Predicted distribution of the projected PbT-II cells in IgG and anti-IL-27 mAb-treated mice on day 7 (D) and day 28 (E) after Pcc infection as density contours in a UMAP plot of a CD4$^+$ T cell reference map (Andreatta *et al*, 2022). The bar graphs represent the proportions of the PbT-II cells projected in the indicated reference subtype.

© 2023 The Authors

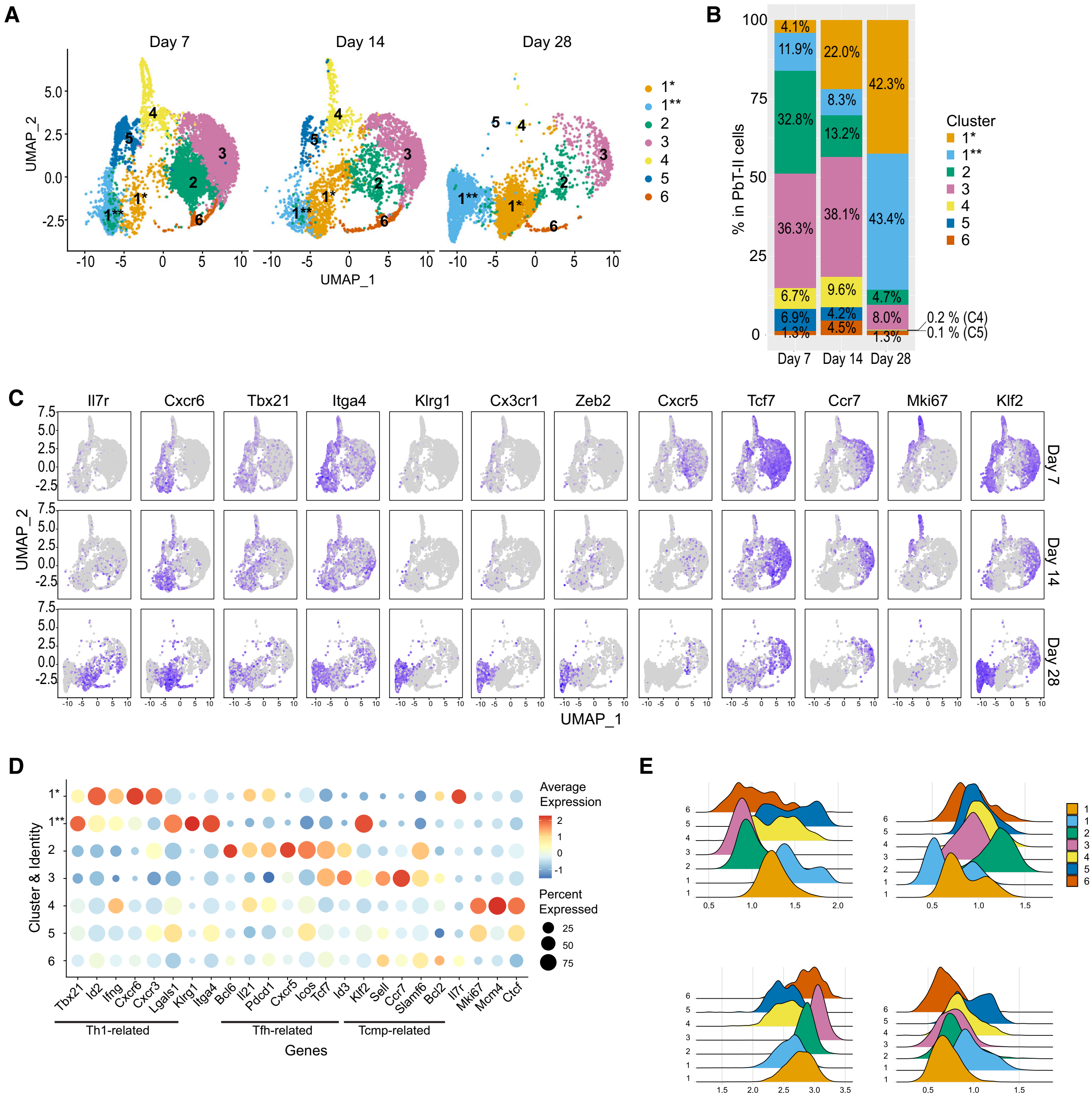

Figure EV4. scRNA-seq analysis suggests transition of CD4$^+$ T cell subsets under IL-27-neutralization condition. Related to Fig 5.

scRNA-seq data of PbT-II cells from Pcc-infected anti-IL-27 mAb-treated mice (day7, 14, and 28) were pooled, and unsupervised clustering was performed.

A UMAP plot colored by gene expression clustering.

B Proportions (%) of each cluster for each time point.

C Feature plots of indicated genes across cell clusters as distributed in UMAP plots.

D Dot plots showing the expression of Th1-, Tfh-, Tmem-, and proliferation-associated genes in each cluster. Dot colors represent the intensity of expression, while dot size represents the proportion of cells with the corresponding expression.

E Ridge plots of PbT-II cell clusters showing the expression of published CD4$^+$ T cell signature genes (Ciucci *et al*, 2019).

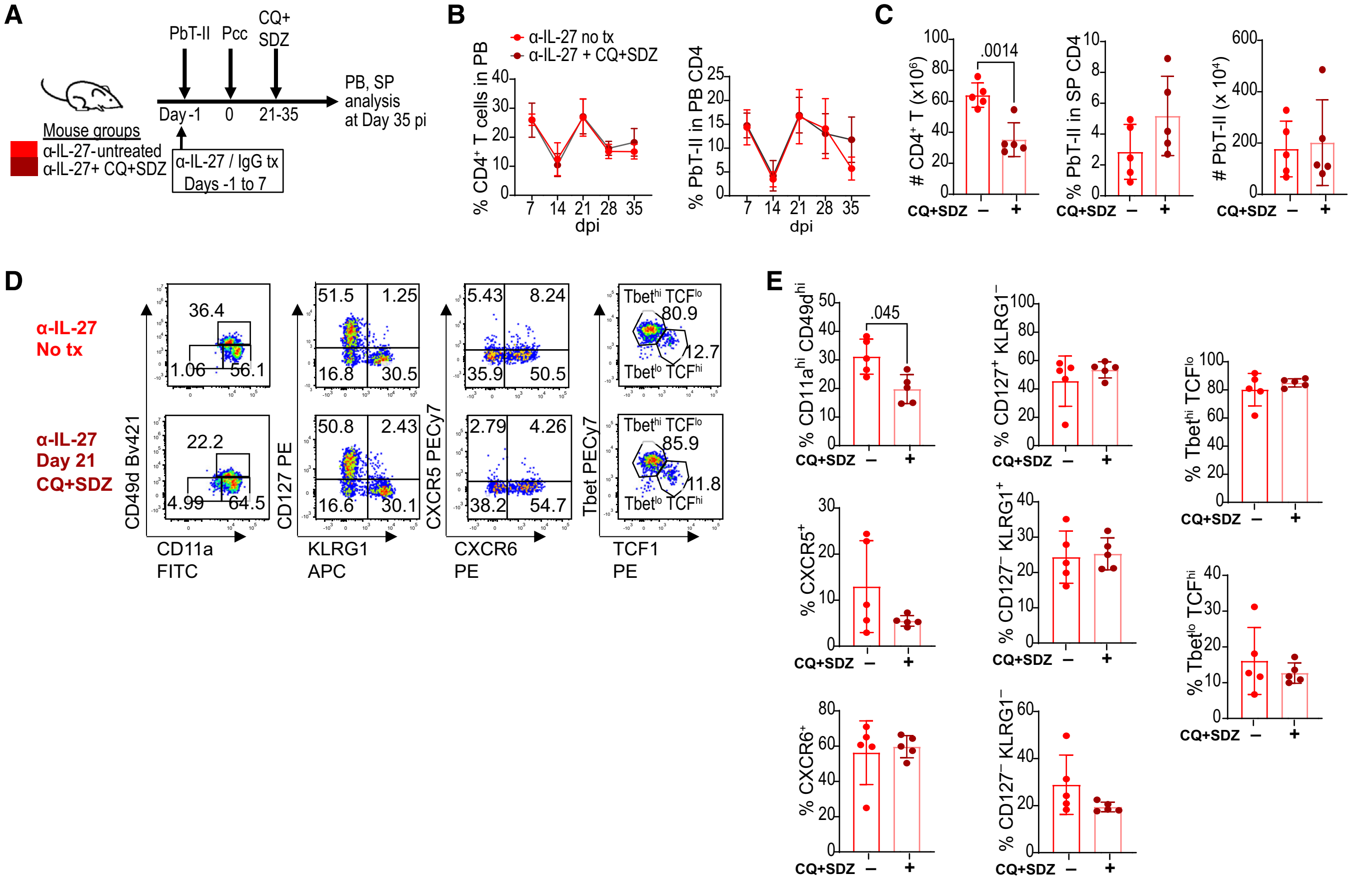

Figure EV5. Antimalarial treatment during chronic phase of Pcc infection does not affect the persistence and phenotype of memory PbT-II cells. Related to Fig 6.

B6 mice were transferred with PbT-II cells, treated with anti-IL-27 mAb, and treated (dark red) or not treated (red) with antimalarial drugs starting 21 days after Pcc infection (n = 5 biological replicates per treatment group). PB was monitored weekly and PbT-II cells in the spleen were analyzed 35 days post-Pcc infection.

- A Experimental scheme.
- B Kinetics of proportions of $CD4^+$ T cells in PB and of PbT-II cells in PB $CD4^+$ T cells.
- C Total number of $CD4^+$ T cells in spleen and proportions within $CD4^+$ T cells and total number of PbT-II cells in spleen (n = 5 mice/group).
- D Representative flow cytometry profiles of splenic PbT-II cells on day 35 of Pcc infection.
- E Proportions of PbT-II cells with the indicated phenotype and those expressing the indicated transcription factors 35 days after Pcc infection (n = 5 mice/group).

Data information: Representative data of two independent experiments are shown. Statistical significance was assessed by Student's t test (P values (< 0.05) shown in black) or Mann–Whitney U test, depending on normality assessment. Error bars represent SD.

© 2023 The Authors
