# Supplementary material for: IL‐27 produced during acute malaria infection regulates Plasmodium‐specific memory CD4 + T cells
Source: EMBO Mol Med. 2023 Oct 19;15(12):e17713. doi: 10.15252/emmm.202317713 (PMC10701605; doi:10.15252/emmm.202317713)
Supplement: Supplementary file 11 [file EMMM-15-e17713-s009.pdf]

EMBO Molecular Medicine

IL-27 produced during acute malaria infection regulates Plasmodium-specific memory CD4+ T cells

Maria Lourdes Macalinao, Shin-Ichi Inoue, Sanjaadorj Tsogtsaikhan, Hirotaka Matsumoto, Ganchimeg Bayarsaikhan, Jiun-Yu Jian, Kazumi Kimura, Yoshiaki Yasumizu, Tsuyoshi Inoue, Hiroki Yoshida, Julius Hafalla, Daisuke Kimura, and Katsuyuki Yui
DOI: 10.15252/emmm.202317713

Corresponding authors: Katsuyuki Yui (katsu@nagasaki-u.ac.jp)

Review Timeline:

Submission Date:	14th Mar 23
Editorial Decision:	31st Mar 23
Revision Received:	21st May 23
Editorial Decision:	6th Jun 23
Revision Received:	5th Sep 23
Editorial Decision:	18th Sep 23
Revision Received:	27th Sep 23
Accepted:	28th Sep 23

Editor: Zeljko Durdevic

Transaction Report:

(Note: With the exception of the correction of typographical or spelling errors that could be a source of ambiguity, letters and reports are not edited. Depending on transfer agreements, referee reports obtained elsewhere may or may not be included in this compilation. Referee reports are anonymous unless the Referee chooses to sign their reports.)

31st Mar 2023

Dear Prof. Yui,

Thank you for the submission of your manuscript to EMBO Molecular Medicine. We have now received feedback from the two reviewers who agreed to evaluate your manuscript. As you will see from the reports below, while referee #2 supports publication of the manuscript raising important but minor criticism, referees #1 recognizes potential interest of the study but also raise serious concerns that should be addressed in a major revision of the current manuscript. If you would like to discuss further the points raised by the referees, I am available to do so via email or video. Let me know if you are interested in this option.

Further consideration of a revision that addresses reviewers' concerns in full will entail a second round of review. EMBO Molecular Medicine encourages a single round of revision only and therefore, acceptance or rejection of the manuscript will depend on the completeness of your responses included in the next, final version of the manuscript. For this reason, and to save you from any frustrations in the end, I would strongly advise against returning an incomplete revision.

We would welcome the submission of a revised version within three months for further consideration. Please let us know if you require longer to complete the revision.

I look forward to receiving your revised manuscript.

Yours sincerely,

Zeljko Durdevic

Zeljko Durdevic
Editor
EMBO Molecular Medicine

*****

When submitting your revised manuscript, please carefully review the instructions that follow below. We perform an initial quality control of all revised manuscripts before re-review; failure to include requested items will delay the evaluation of your revision.

We require:

1) A .docx formatted version of the manuscript text (including legends for main figures, EV figures and tables). Please make sure that the changes are highlighted to be clearly visible.

2) Individual production quality figure files as .eps, .tif, .jpg (one file per figure). For guidance, download the 'Figure Guide PDF': (https://www.embopress.org/page/journal/17574684/authorguide#figureformat).

3) A .docx formatted letter INCLUDING the reviewers' reports and your detailed point-by-point responses to their comments. As part of the EMBO Press transparent editorial process, the point-by-point response is part of the Review Process File (RPF), which will be published alongside your paper.

4) A complete author checklist, which you can download from our author guidelines (https://www.embopress.org/page/journal/17574684/authorguide#submissionofrevisions). Please insert information in the checklist that is also reflected in the manuscript. The completed author checklist will also be part of the RPF.

5) Please note that all corresponding authors are required to supply an ORCID ID for their name upon submission of a revised manuscript.

6) It is mandatory to include a 'Data Availability' section after the Materials and Methods. Before submitting your revision, primary

datasets produced in this study need to be deposited in an appropriate public database, and the accession numbers and database listed under 'Data Availability'. Please remember to provide a reviewer password if the datasets are not yet public (see https://www.embopress.org/page/journal/17574684/authorguide#dataavailability).

In case you have no data that requires deposition in a public database, please state so in this section. Note that the Data Availability Section is restricted to new primary data that are part of this study.

7) For data quantification: please specify the name of the statistical test used to generate error bars and P values, the number (n) of independent experiments (specify technical or biological replicates) underlying each data point and the test used to calculate p-values in each figure legend. The figure legends should contain a basic description of n, P and the test applied. Graphs must include a description of the bars and the error bars (s.d., s.e.m.). See also 'Figure Legend' guidelines: https://www.embopress.org/page/journal/17574684/authorguide#figureformat

8) At EMBO Press we ask authors to provide source data for the main manuscript figures. Our source data coordinator will contact you to discuss which figure panels we would need source data for and will also provide you with helpful tips on how to upload and organize the files.

9) Our journal encourages inclusion of *data citations in the reference list* to directly cite datasets that were re-used and obtained from public databases. Data citations in the article text are distinct from normal bibliographical citations and should directly link to the database records from which the data can be accessed. In the main text, data citations are formatted as follows: "Data ref: Smith et al, 2001" or "Data ref: NCBI Sequence Read Archive PRJNA342805, 2017". In the Reference list, data citations must be labeled with "[DATASET]". A data reference must provide the database name, accession number/identifiers and a resolvable link to the landing page from which the data can be accessed at the end of the reference. Further instructions are available at .

10) We replaced Supplementary Information with Expanded View (EV) Figures and Tables that are collapsible/expandable online. A maximum of 5 EV Figures can be typeset. EV Figures should be cited as 'Figure EV1, Figure EV2" etc... in the text and their respective legends should be included in the main text after the legends of regular figures.

- For the figures that you do NOT wish to display as Expanded View figures, they should be bundled together with their legends in a single PDF file called *Appendix*, which should start with a short Table of Content. Appendix figures should be referred to in the main text as: "Appendix Figure S1, Appendix Figure S2" etc.

- Additional Tables/Datasets should be labeled and referred to as Table EV1, Dataset EV1, etc. Legends have to be provided in a separate tab in case of .xls files. Alternatively, the legend can be supplied as a separate text file (README) and zipped together with the Table/Dataset file.

See detailed instructions here:

.

11) The paper explained: EMBO Molecular Medicine articles are accompanied by a summary of the articles to emphasize the major findings in the paper and their medical implications for the non-specialist reader. Please provide a draft summary of your article highlighting

- the medical issue you are addressing,

- the results obtained and

- their clinical impact.

This may be edited to ensure that readers understand the significance and context of the research. Please refer to any of our published articles for an example.

12) For more information: There is space at the end of each article to list relevant web links for further consultation by our readers. Could you identify some relevant ones and provide such information as well? Some examples are patient associations, relevant databases, OMIM/proteins/genes links, author's websites, etc...

13) Author contributions: You will be asked to provide CRediT (Contributor Role Taxonomy) terms in the submission system. These replace a narrative author contribution section in the manuscript.

14) A Conflict of Interest statement should be provided in the main text.

15) Every published paper now includes a 'Synopsis' to further enhance discoverability. Synopses are displayed on the journal webpage and are freely accessible to all readers. They include a short stand first (maximum of 300 characters, including space) as well as 2-5 one-sentences bullet points that summarizes the paper. Please write the bullet points to summarize the key NEW findings. They should be designed to be complementary to the abstract - i.e. not repeat the same text. We encourage inclusion of key acronyms and quantitative information (maximum of 30 words / bullet point). Please use the passive voice. Please attach these in a separate file or send them by email, we will incorporate them accordingly.

Please also suggest a striking image or visual abstract to illustrate your article as a PNG file 550 px wide x 300-600 px high.

EMBO Molecular Medicine has a "scooping protection" policy, whereby similar findings that are published by others during review or revision are not a criterion for rejection. Should you decide to submit a revised version, I do ask that you get in touch after three months if you have not completed it, to update us on the status.

Please note: When submitting your revision you will be prompted to enter your funding and payment information. This will allow Wiley to send you a quote for the article processing charge (APC) in case of acceptance. This quote takes into account any reduction or fee waivers that you may be eligible for. Authors do not need to pay any fees before their manuscript is accepted and transferred to the publisher.

EMBO Press participates in many Publish and Read agreements that allow authors to publish Open Access with reduced/no publication charges. Check your eligibility: https://authorservices.wiley.com/author-resources/Journal-Authors/open-access/affiliation-policies-payments/index.html

***** Reviewer's comments *****

Referee #1 (Comments on Novelty/Model System for Author):

The choice of Plasmodium chabaudi chabaudi is adequate. Still, the use of P.berghei for re-infection experiments has to be completed with an autologous infection that will better recapitulate the reality in human populations.

Referee #1 (Remarks for Author):

The manuscript "IL-27 produced during acute malaria infection regulates Plasmodium-specific memory CD4+ T cells" by Macalinao et al., studies the role and repercussion of IL-27 during acute malaria infection in a non-lethal model of experimental malaria. By using antibodies neutralizing IL-27 and Pb specific transgenic cells (PbT-II cells) the authors attempt to define events were parasites responding CD4+T cells are targeted by IL-27. The manuscript is well written, clear and experiments well designed and executed. Nevertheless several and important flaws diminished author conclusions.
Major points:
-Authors used the markers CD49d and CD11a to identify antigen-responding/experienced cells as measure of anti-parasite response. These markers are well known and accepted by the community. Nevertheless is not clear if authors gate on CD49dhiCD11ahi cells to then analyze other markers like CD127 and KLGR1. By analyzing the gating strategy the reader can conclude that CD49d CD11a are only used to determine the proportion of PbT-II responding to infection but without using these markers to really quantified T cell-specific responses. Could authors clarified this point?
-Fig 2. When mice receiving PbT-II cells and infected with Pcc are treated with Ctrl IgG or anti-IL-27 it appears that long live (CD127+KLRG1-) and short lived cells(CD127-KLRG1h+) are both increased on the IL-27 treated mice. Authors acknowledge that there was a slight difference only in the effect of IL-27 but this is maybe due to the gating strategy that is not made on CD49dhiCD11ahi. Could authors show the same results by gating on this population that will represent the real antigen response?
-Figure 4. Authors use scRNAseq analysis the different cellular memory responses modulated by IL-27. For this they sort cells PbT-II cells transferred onto mice that were then infected and treated with Ctrl IgG or anti-IL-27 IgG. I think here is a problem because in Fig EV3A the sorting strategy shows that cells sorted were CD45.1 that correspond with PbT-II cells. Nevertheless no antigen responding markers were used, making impossible to know if the differences on the response was due to the treatment or to the difference on the number of antigen responding cells.
In addition in Figure 5 authors only analyzed responses on cells coming from infected and anti- IL-27 IgG-treated mice without taking in account Ctrl-IgG groups. Do they perform analysis of these group? If yes they must to add them or perform those experiments. Moreover they compared their data with published data and used them as a control group, nevertheless the published data used are from experiments performed with a different strain of P. chabaudi making this comparison useless. Could authors used their own model to make this interesting comparison?
-Figure 7. Authors analyzed the memory responses elicited by anti-IL-27 therapy by infecting, eliminating the parasite with drugs and reinfecting again with a different parasite strain. They show that treatment with IL-27 but no with drugs increases survival. Several things are unclear here.
Did authors verified that in the absence of drug treatment which gave the best results parasites from first infection are completely cleared? If not the difference could be only a resistance due to adaptive immune response to first parasite.
I understand by authors used a lethal model but they should also use the same parasite to really asses the effect of anti-IL-27

treatment because lethal infection could trigger a more acute response that could be deleterious. Please correct
-Authors consider that anti-IL-27 treatment is identical to absence of IL-27. This may not be the case, at least authors should check the presence of IL-27 by ELISA or other method to be able to say "absence of IL-27"
Minor points:
- Sentence "Sterile immunity is hardly achieved in malaria" has to be changed to "...is never achieved"
-Figure 1, Ebi3-/- and Il-27-/- infected mice have different CD4 T cells profiles although both strains lack of IL-27. Could authors explain the differences?
-Authors used difference treatment of anti-IL27 and at the end is hard to know which one they used for the further analysis. Could they clarify this point?
-The number of mice used in each replicate is not clear in many of the experiments, could the authors amend this? In addition several results presented as significant (example Fig 7E among others) don't present exact p-values. Could authors present the exact p-values and clarified normal distribution and statistical tests?
-Could authors explain why in the presence of anti-IL-27 frequency of Tfh cells decreased with the time (Fig 5).

Referee #2 (Remarks for Author):

Macalino et al have investigated the role of the cytokine IL-27 in the development of antimalarial immunity in the P. chabaudi mouse model. This is a timely and important study. All experiments are well-designed, performed, and analyzed. The conclusions are supported by the results provided.

Specific comments
1. Introduction: the authors cite review articles to mention that protective immunity develops after repeated infection. It would be better to cite the original article describing this: i.e.
1-Koch R. Zweiter Bericht uber die Thatigkeit der MalariaExpedition. Dtsch Med Wochenschr 1900; 26(5): 88-90. English Translation in Brit Med J Feb 10, 1900, 325-327.
2-Koch R. Dritter Bericht uber die Thatigkeit der Malaria-Expedition. Dtsch Med Wochenschr 1900; 26(17): 281-284. English Translation in Brit. Med. J. May 12, 1900, 1183-1186.
3-Koch R. Vieter Bericht uber die Thatigkeit der Malaria-Expedition. Dtsch Med Wochenschr 1900; 26(25): 397-398. English translation in Brit. Med. J. June 30, 1900, 1597-1598.
2. Page 14: suggesting its dependence instead of suggesting tits dependence
3. What is the driver of IL-27 production during the infection? This should be discussed.
4. The authors should give more info on the parasites used: it is not mentioned if the lines used were cloned or uncloned, and if clones were used, the name of the clones should be provided.

EMM-2023-17713

Point-by-point response

Dear Referees,

We thank the reviewers for appreciating the novelty and significance of our work and for providing concise comments and suggestions to our manuscript. The comments enabled us to enhance and improve the clarity of our manuscript. We address the comments from reviewers below.

Referee #1 (Comments on Novelty/Model System for Author):
The choice of Plasmodium chabaudi chabaudi is adequate. Still, the use of P. berghei for re-infection experiments has to be completed with an autologous infection that will better recapitulate the reality in human populations.

While we agree with the reviewer that performing the re-challenge infection experiments using homologous parasites (Pcc) is warranted, it is well reported that Pcc-infection induces potent protective response in C57BL/6 mice that can control the parasitemia levels to less than 0.3% after re-challenge with the same parasite even at 120 days post-infection (Freitas do Rosario et al, J Immunol, 181: 8344-8355, 2008). Given that the anti-IL-27 mAb treatment enhanced the immune response and further reduced the parasitemia after re-challenge to a level less than those of control mice, it will not be informative to evaluate the enhancement of the protective effect of anti-IL-27 mAb treatment in rechallenge experiment with homologous parasites. We have used a challenge with the heterologous parasite, *P. berghei* ANKA. The decision to use a heterologous challenge approach is based on previous work by Prof. M. Good's group and from our laboratory (MF Good *et al*, J Clin Invest, 123: 3353-3362, 2013; Nakamae *et al*, Parasitol Int, 70: 5-15, 2019), and relies on well published cross-reactive cell-mediated immune responses between the two rodent malaria species (Elliott *et al*, Infect Immun, 73: 2478-2485, 2005). PbT-II cells express TCR specific for *Plasmodium* hsp90 that is shared among Pcc, *P. berghei* ANKA and *P. falciparum* (Fernandez-Ruiz *et al*, I. Immunol, 199: 4165-4179, 2017; Enders *et al*, Curr Res Immunol, 2: 79-92, 2021). We have added an explanation of this reasoning in the results section (lines 346-350).

Referee #1 (Remarks for Author):

The manuscript is well written, clear and experiments well designed and executed. Nevertheless several and important flaws diminished author conclusions.

Major points:

-Authors used the markers CD49d and CD11a to identify antigen-responding/experienced cells as measure of anti-parasite response. These markers are well known and accepted by the community. Nevertheless is not clear if authors gate on CD49dhiCD11ahi cells to then analyze other markers like CD127 and KLGR1. By analyzing the gating strategy the reader can conclude that CD49d CD11a are only used to determine the proportion of PbT-II responding to infection but without using these markers to really quantified T cell-specific responses. Could authors clarified this point?

We thank the reviewer for seeking clarity on the CD11a/CD49d phenotype of *Plasmodium*-specific CD4^{+} T cells. As the reviewer mentioned, CD11a^{hi}CD49d^{hi} is used as a proxy marker for antigen-specific activated CD4^{+} T cells in viral and malaria infection models (McDermott *et al*, J. Immunol, 187: 188, 2011; Butler *et al*, Nat Immunol, 13: 188, 2011). However, we adoptively transferred PbT-II cells from TCR-transgenic mice to visualize *Plasmodium* antigen-specific CD4^{+} T cell responses. We and others previously showed that majority of PbT-II cells transferred extensively proliferate and are recruited to the immune response after Pcc infection, as evidenced by the dilution of the CFSE dye that labeled the PbT-II cells (Fernandez-Ruiz *et al*, I. Immunol, 199: 4165-4179, 2017; Jian *et al*, Int Immunol, 33: 409-422, 2021). Further, we have reported that *Plasmodium* antigen-specific CD4^{+} T cells develop not only to CD11a^{hi}CD49d^{hi} but also to CD11a^{hi}CD49d^{lo} cells during Pcc infection of mice that received PbT-II cells. CD11a^{hi}CD49d^{hi} cells are Th1 type cells that localize mainly in the white pulp, while CD11a^{hi}CD49d^{lo} cells include Tfh cells and localize mainly in the red pulp. Notably, transferred PbT-II cells in uninfected mice maintain a CD11a^{lo}CD49d^{lo}phenotype (Jian et al, Int Immunol, 33: 409-422, 2021). Therefore, based on these results, we have gated for the total PbT-II cells as indicated by gating strategy in Fig. EV1 A.

To help understand that all PbT-II cells are activated and develop to functional CD4^{+} T cells after Pcc infection without previous knowledge of the cited paper (Jian et al, Int Immunol, 33: 409-422, 2021), we have added Fig. EV1C, showing the phenotype of PbT-II cells from mice 7 days after infection and the results are further explained in

lines 173-177 of the manuscript. Again, majority of PbT-II cells (>95%) expressed the activation marker CD11a, indicating that they were activated. In both IgG- and anti-IL-27 mAb-treated mice, CD49d^{hi} PbT-II cells were mainly Th1-type that express CXCR6 and transcription factor Tbet, while CD49d^{lo} PbT-II cells were composed mainly of Tfh-like cells expressing CXCR5 and TCF1.

-Fig 2. When mice receiving PbT-II cells and infected with Pcc are treated with Ctrl IgG or anti-IL-27 it appears that long live (CD127+KLRG1-) and short lived cells (CD127-KLRG1h+) are both increased on the IL-27 treated mice. Authors acknowledge that there was a slight difference only in the effect of IL-27 but this is maybe due to the gating strategy that is not made on CD49dhiCD11ahi. Could authors show the same results by gating on this population that will represent the real antigen response?

We thank the reviewer for this comment, which follows from the general comment above. Based on our previous work, majority of PbT-II cells clonally expand, upregulate activation marker, CD11a, and functionally differentiate during Pcc infection. In uninfected PbT-II-transferred mice, majority of PbT-II cells remain CD11a^{lo}CD49d^{lo} (Jian et al, Int Immunol, 33: 409-422, 2021). Therefore, the phenotypic analysis of PbT-II cells was performed on total PbT-II cells, with the gating strategy shown in Fig. EV1A. As mentioned above, activated *Plasmodium*-specific CD4^{+} T cells develop into CD11a^{hi}CD49d^{hi} Th1-type cells and CD11a^{hi}CD49d^{lo} cells that include Tfh-type cells in both anti-IL-27 Ab treated and control mice (Fig. EV1C). We have now clarified this in the revision through Fig. EV1, and explained in lines 173-177 of the manuscript.

-Figure 4. Authors use scRNAseq analysis the different cellular memory responses modulated by IL-27. For this they sort cells PbT-II cells transferred onto mice that were then infected and treated with Ctrl IgG or anti-IL-27 IgG. I think here is a problem because in Fig EV3A the sorting strategy shows that cells sorted were CD45.1 that correspond with PbT-II cells. Nevertheless no antigen responding markers were used, making impossible to know if the differences on the response was due to the treatment or to the difference on the number of antigen responding cells.

We appreciate this comment by the reviewer. However, as we explained above, all PbT-II cells recognize the same antigen, are activated, clonally expand, and functionally differentiate during *Plasmodium* infection. Antigen responding markers of these sorted cells are shown in the newly added Fig. EV1C, indicating that they express the

activation marker, CD11a. Furthermore, the single cell RNA-seq analysis of these PbT-II cells, as shown in Figure 4, clearly demonstrated that they undergo differentiation to Th1 (cluster 1), Tfh (cluster 2) and Tcmp-like cells 7 days after infection. Single cell RNA-seq analysis of PbT-II cells previously published also made use of sort-purified total PbT-II cells from Pcc-infected mice (Lonnberg *et al*, Sci Immunol, 2: eaal2192, 2017; Soon *et al,* Nat Immunol, 21: 1597-1610, 2020).

In addition in Figure 5 authors only analyzed responses on cells coming from infected and anti- IL-27 IgG-treated mice without taking in account Ctrl-IgG groups. Do they perform analysis of these group? If yes they must to add them or perform those experiments. Moreover they compared their data with published data and used them as a control group, nevertheless the published data used are from experiments performed with a different strain of P. chabaudi making this comparison useless. Could authors used their own model to make this interesting comparison?

We thank the reviewer for this comment. In the flow cytometry analysis (Fig. 2), we found 3 major populations in PbT-II cells in anti-IL-27 Ab-treated mice on 28 days after infection: $CD127^{+}KLRG1^{-}$, $CD127^{-}KLRG1^{+}$, and $CD127^{-}KLRG1^{-}$ cells. In particular, the former 2 populations are phenotypically comparable to memory precursor and terminal effector $CD8^{+}$ T cells. Therefore, we performed scRNA-seq in combination with CITE-seq, which allows identification of CD127 and KLRG1 surface expression, to determine what cells these subpopulations are (Fig. 5. A-E). We have explained the reason for the scRNAseq analysis in lines 229-232 of the manuscript, and added the explanation of day 28 analysis in lines 250. We did not intend to compare the effect of anti-IL-27 mAb treatment with IgG control by scRNA-seq experiment and therefore, did not need scRNA-seq data of IgG control on day 28 after infection.

We showed the comparison of scRNA-seq data of PbT-II cells from anti-IL-27 mAb treated-mice on day 28 post-infection with the published data of PbT-II cells from mice infected with *P. chabaudi* for 28 days (Fig EV4A, B), given that the data were available publicly (Soon *et al*, Nat Immunol, 21: 1597-1610, 2020). Soon's study and ours used the same TCR-transgenic $CD4^{+}$ T cells, PbT-II, and the identical parasite, *Plasmodium chabaudi chabaudi* AS, although the abbreviation of the parasite name was Pcc in our study and was referred to as PcAS in theirs. We showed the comparison of overall quality of 2 scRNA-seq data in heat map of top 10 conserved genes in each cluster (Fig EV4B) and demonstrated that the gene expression of PbT-II cells in the same cluster is

comparable between our and their data. We would like to emphasize that this figure is not a part of our main figures, but shown as Fig. EV4 (comparable to a supplementary figure).

-Figure 7. Authors analyzed the memory responses elicited by anti-IL-27 therapy by infecting, eliminating the parasite with drugs and reinfecting again with a different parasite strain. They show that treatment with IL-27 but no with drugs increases survival. Several things are unclear here.
◊Did authors verified that in the absence of drug treatment which gave the best results parasites from first infection are completely cleared? If not the difference could be only a resistance due to adaptive immune response to first parasite.

We thank the reviewers for this query. The parasitemia was nearly undetectable levels in smear samples 2 months after infection with Pcc in both anti-IL-27 mAb-treated and untreated mice. However, we assume that low levels of parasites were maintained in mice not treated with antimalarial drugs, because it has been shown that Pcc infection in C57BL/6 mice results in chronicity and parasites can persist for more than 2 months (Achtman et al, Parasite Immunol, 29: 435-444, 2007). The chronic infection of malaria is known to enhance immune response to the subsequent infection, which is termed premunition or concomitant immunity. Our study compared the effect of anti-IL-27 antibody treatment under the condition and demonstrated that early anti-IL-27 mAb treatment enhanced the concomitant immunity. In addition, our antimalarial treatment experiments indicated that active *Plasmodium* infection is critical for the maintenance of protective immunity (Fig. 6, 7). However, once established, *Plasmodium*-specific memory-like $CD4^+$ T cells can be maintained at least 3 weeks without active infection (Fig. EV5).

◊I understand by authors used a lethal model but they should also use the same parasite to really asses the effect of anti-IL-27 treatment because lethal infection could trigger a more acute response that could be deleterious. Please correct

We acknowledge that an assessment with the re-challenge infection using Pcc is important, and we also considered it. However, it has previously been reported that the initial Pcc-infection induces potent protective immunity in C57BL/6 mice, wherein a re-challenge with the same parasite even at 120 days post-infection can control the

parasitemia to levels as low as less than 0.3% (Freitas do Rosario et al, J Immunol, 181: 8344-8355, 2008). Therefore, a homologous rechallenge would not be informative to evaluate the effect of anti-IL-27 mAb treatment, as we have established that it enhances the immune response, and is then expected to further reduce the parasitemia after re-challenge with Pcc. We added an explanation of this reasoning in line 346-350 of the manuscript.

-Authors consider that anti-IL-27 treatment is identical to absence of IL-27. This may not be the case, at least authors should check the presence of IL-27 by ELISA or other method to be able to say "absence of IL-27"

We carefully checked the text for the expression,"anti-IL-27 treatment is identical to absence of IL-27," and confirmed that we did not use nor allude to this phrase. What we have shown in this manuscript is that the transient treatment with anti-IL-27 mAb in Pcc-infected mice induced *Plasmodium*-specific $CD4^+$ T cell immune responses comparable to those generated in mice that genetically lack the IL-27 gene. In anti-IL-27 mAb-treated mice, IL-27 is present in mice but cannot bind to IL-27 receptor because it is neutralized with the antibody during the treatment period.

Minor points:

- Sentence "Sterile immunity is hardly achieved in malaria" has to be changed to "...is never achieved"

We agree that field studies in endemic areas do not find any evidence of sterile immunity, and the consensus of the scientific community is that sterile immunity cannot be achieved by natural infection. However, we believe that no one has carefully provided solid evidence that sterile immunity cannot be achieved by natural infection. Therefore, we used a toned-down phrase "sterile immunity is hardly achieved in malaria".

-Figure 1, Ebi3-/- and Il-27-/- infected mice have different CD4 T cells profiles although both strains lack of IL-27. Could authors explain the differences?

We appreciate the reviewer for the comment. We added possible interpretation for this difference in lines 140-141 of the manuscript.

-Authors used difference treatment of anti-IL27 and at the end is hard to know which one they used for the further analysis. Could they clarify this point?

In the experiment shown in Fig. 1, we tested 3 different treatment schemes, regular (day -1-day 19), early (day -1-day 7), and late (day 11-19) period and found that early treatment gave the results comparable to the regular treatment. We further tested the number of doses required to elicit the observed effect on PbT-II cells within the early (day-1 to day 7) period in Fig. EV1, and determined the experimental condition of IL-27 neutralization that we used in this study (i.e., the 4-dose treatment provided the most consistent results). Therefore, all IL-27 neutralization experiments described Fig. 2-7 and EV2-EV5 were performed under the early 4-dose treatment condition, in which antibody was injected on days -1, 2, 5, and 7 days after Pcc-infection. We described this decision of the experimental condition in lines 162-163 of the manuscript.

-The number of mice used in each replicate is not clear in many of the experiments, could the authors amend this?

We appreciate the reviewer for the comment. We amended legends of Figure 1-3, 6, 7, Figure EV1, EV2, EV5 and appendix and clarified the number of animals used in each experiment as well as numbers of experiments performed. In addition, we replaced data in Fig. 1 D and G and showed pooled data of all experiments, which better represent experimental results.

In addition several results presented as significant (example Fig 7E among others) don't present exact p-values. Could authors present the exact p-values and clarified normal distribution and statistical tests?

We appreciate the reviewer for the comment. We amended Figures 1, 2, 3, 6, 7, EV1, EV2, EV5 and appendix and have added exact p-values in all of the significant differences ($p>0.05$). The method of statistical analysis is clearly described in lines 648-652 of the manuscript.

-Could authors explain why in the presence of anti-IL-27 frequency of Tfh cells decreased with the time (Fig 5).

We thank the reviewer for seeking clarity on this. In Fig. 5F, we showed the proportion

of each cluster in mice treated with anti-IL-27 mAb on day 7, 14 and 28 after infection. As the reviewer mentioned, proportion of Tfh cells (cluster 2) in total PbT-II cells were decreased over time. The reduction of Tfh cells after Pcc infection has been reported previously in studies using PbT-II cells and Pcc infection model (Soon et. al, Nature Immunol, 21: 1597-1610, 2020), and is not unique to anti-IL-27 mAb-treated mice. In fact, total numbers of Tfh PbT-II cells ($CXCR5^+$ cells) were not significantly different between anti-IL-27 mAb-treated and control mice at all timepoints tested during the course of the Pcc infection (Fig. 2C). However, the proportional reduction in Tfh cells was more pronounced in anti-IL-27 mAb-treated mice, because Th1-type PbT-II cells steadily increased starting day 14, and were maintained at high numbers in anti-IL-27 mAb-treated mice, while they were reduced in control mice (Fig. 2). This observation was noted in lines 193-199 of the manuscript.

Referee #2 (Remarks for Author):

This is a timely and important study. All experiments are well-designed, performed, and analyzed. The conclusions are supported by the results provided.

Specific comments

1. Introduction: the authors cite review articles to mention that protective immunity develops after repeated infection. It would be better to cite the original article describing this: i.e.

We thank the reviewer's comment that reminded us that Dr. Robert Koch already recognized the development of protective immunity after repeated infection in malaria during his time. We cited his literature describing acquired immunity in malaria, as recommended by the reviewer, in line 59 of the manuscript.

2. Page 14: suggesting its dependence instead of suggesting tits dependence

Thank you very much for informing this misspelling. We have corrected it (line 306).

3. What is the driver of IL-27 production during the infection? This should be discussed.

We expounded on the cellular source and stimuli leading to the production of IL-27 in lines 392-394 of the manuscript.

4. The authors should give more info on the parasites used: it is not mentioned if the lines used were cloned or uncloned, and if clones were used, the name of the clones should be provided.

We thank the reviewer for seeking clarity on this. The Pcc (AS clone) that we used in this study is a cloned parasite. We added a more detailed description of the used *Plasmodium* parasites in lines 520-522 of the manuscript.

6th Jun 2023

Dear Prof. Yui,

Thank you for the submission of your revised manuscript to EMBO Molecular Medicine. We have now heard back from the two referees who we asked to re-evaluate your manuscript. As you will see from the reports below, while the referee #2 supports publication of your manuscript, referee #1 acknowledges again the interest of the study but also raises concerns that have not been satisfactorily addressed in the revised manuscript and that should be addressed in an additional and final round of major revision.

Further consideration of a revision that addresses reviewer's concerns in full will entail an additional round of review. Acceptance or rejection of the manuscript will depend on the completeness of your responses included in the next, final version of the manuscript. For this reason, and to save you from any frustrations in the end, I would strongly advise against returning an incomplete revision.

We would welcome the submission of a revised version within three months for further consideration. Please let us know if you require longer to complete the revision.

I look forward to receiving your revised manuscript.

Yours sincerely,

Zeljko Durdevic

Zeljko Durdevic
Editor
EMBO Molecular Medicine

*****

When submitting your revised manuscript, please carefully review the instructions that follow below. We perform an initial quality control of all revised manuscripts before re-review; failure to include requested items will delay the evaluation of your revision.

We require:

1) A .docx formatted version of the manuscript text (including legends for main figures, EV figures and tables). Please make sure that the changes are highlighted to be clearly visible.

2) Individual production quality figure files as .eps, .tif, .jpg (one file per figure). For guidance, download the 'Figure Guide PDF': (https://www.embopress.org/page/journal/17574684/authorguide#figureformat).

3) A .docx formatted letter INCLUDING the reviewers' reports and your detailed point-by-point responses to their comments. As part of the EMBO Press transparent editorial process, the point-by-point response is part of the Review Process File (RPF), which will be published alongside your paper.

4) A complete author checklist, which you can download from our author guidelines (https://www.embopress.org/page/journal/17574684/authorguide#submissionofrevisions). Please insert information in the checklist that is also reflected in the manuscript. The completed author checklist will also be part of the RPF.

5) Please note that all corresponding authors are required to supply an ORCID ID for their name upon submission of a revised manuscript.

6) It is mandatory to include a 'Data Availability' section after the Materials and Methods. Before submitting your revision, primary

datasets produced in this study need to be deposited in an appropriate public database, and the accession numbers and database listed under 'Data Availability'. Please remember to provide a reviewer password if the datasets are not yet public (see https://www.embopress.org/page/journal/17574684/authorguide#dataavailability).

In case you have no data that requires deposition in a public database, please state so in this section. Note that the Data Availability Section is restricted to new primary data that are part of this study.

7) For data quantification: please specify the name of the statistical test used to generate error bars and P values, the number (n) of independent experiments (specify technical or biological replicates) underlying each data point and the test used to calculate p-values in each figure legend. The figure legends should contain a basic description of n, P and the test applied. Graphs must include a description of the bars and the error bars (s.d., s.e.m.). See also 'Figure Legend' guidelines: https://www.embopress.org/page/journal/17574684/authorguide#figureformat

8) At EMBO Press we ask authors to provide source data for the main manuscript figures. Our source data coordinator will contact you to discuss which figure panels we would need source data for and will also provide you with helpful tips on how to upload and organize the files.

9) Our journal encourages inclusion of *data citations in the reference list* to directly cite datasets that were re-used and obtained from public databases. Data citations in the article text are distinct from normal bibliographical citations and should directly link to the database records from which the data can be accessed. In the main text, data citations are formatted as follows: "Data ref: Smith et al, 2001" or "Data ref: NCBI Sequence Read Archive PRJNA342805, 2017". In the Reference list, data citations must be labeled with "[DATASET]". A data reference must provide the database name, accession number/identifiers and a resolvable link to the landing page from which the data can be accessed at the end of the reference. Further instructions are available at .

10) We replaced Supplementary Information with Expanded View (EV) Figures and Tables that are collapsible/expandable online. A maximum of 5 EV Figures can be typeset. EV Figures should be cited as 'Figure EV1, Figure EV2" etc... in the text and their respective legends should be included in the main text after the legends of regular figures.

- For the figures that you do NOT wish to display as Expanded View figures, they should be bundled together with their legends in a single PDF file called *Appendix*, which should start with a short Table of Content. Appendix figures should be referred to in the main text as: "Appendix Figure S1, Appendix Figure S2" etc.

- Additional Tables/Datasets should be labeled and referred to as Table EV1, Dataset EV1, etc. Legends have to be provided in a separate tab in case of .xls files. Alternatively, the legend can be supplied as a separate text file (README) and zipped together with the Table/Dataset file.

See detailed instructions here:

.

11) The paper explained: EMBO Molecular Medicine articles are accompanied by a summary of the articles to emphasize the major findings in the paper and their medical implications for the non-specialist reader. Please provide a draft summary of your article highlighting

- the medical issue you are addressing,

- the results obtained and

- their clinical impact.

This may be edited to ensure that readers understand the significance and context of the research. Please refer to any of our published articles for an example.

12) For more information: There is space at the end of each article to list relevant web links for further consultation by our readers. Could you identify some relevant ones and provide such information as well? Some examples are patient associations, relevant databases, OMIM/proteins/genes links, author's websites, etc...

13) Author contributions: You will be asked to provide CRediT (Contributor Role Taxonomy) terms in the submission system. These replace a narrative author contribution section in the manuscript.

14) A Conflict of Interest statement should be provided in the main text.

15) Every published paper now includes a 'Synopsis' to further enhance discoverability. Synopses are displayed on the journal webpage and are freely accessible to all readers. They include a short stand first (maximum of 300 characters, including space) as well as 2-5 one-sentences bullet points that summarizes the paper. Please write the bullet points to summarize the key NEW findings. They should be designed to be complementary to the abstract - i.e. not repeat the same text. We encourage inclusion of key acronyms and quantitative information (maximum of 30 words / bullet point). Please use the passive voice. Please attach these in a separate file or send them by email, we will incorporate them accordingly.

Please also suggest a striking image or visual abstract to illustrate your article as a PNG file 550 px wide x 300-600 px high.

EMBO Molecular Medicine has a "scooping protection" policy, whereby similar findings that are published by others during review or revision are not a criterion for rejection. Should you decide to submit a revised version, I do ask that you get in touch after three months if you have not completed it, to update us on the status.

Please note: When submitting your revision you will be prompted to enter your funding and payment information. This will allow Wiley to send you a quote for the article processing charge (APC) in case of acceptance. This quote takes into account any reduction or fee waivers that you may be eligible for. Authors do not need to pay any fees before their manuscript is accepted and transferred to the publisher.

EMBO Press participates in many Publish and Read agreements that allow authors to publish Open Access with reduced/no publication charges. Check your eligibility: https://authorservices.wiley.com/author-resources/Journal-Authors/open-access/affiliation-policies-payments/index.html

***** Reviewer's comments *****

Referee #1 (Remarks for Author):

Authors from the revised manuscript by Macalaino et al., have decided not to perform additional experiments or add amended figures. In my opinion, they did not require too much work. Even if authors defended their points, the message must be clarified and some data must be obtained. I still found the study interesting but to be fully convinced some modifications should be made

Major points:
-I understand the author's point about using heterologous challenges. Authors claim that "Given that the anti-IL-27 mAb treatment enhanced the immune response and further reduced the parasitemia after re-challenge to a level less than those of control mice," but they do not know what would have been happened with a homologous challenge, their raisoning is speculative only nevertheless if the 0.3 % parasitemia after re-challenge becomes 0 % giving complete protection that will make the difference.
- That "majority of PbT-II cells" is not a sufficient reason to do not to make the gating on antigen-responding cells and then on KLRG1/cD127 or other markers. Authors should do that, which should be easy because they have the data. If they already have done that, they should indicate it on the main figures as gating strategy is insufficient.
- Authors claim, "We did not intend to compare the effect of anti-IL-27 mAb treatment with IgG control by scRNA-seq experiment and, therefore, did not need scRNA-seq data of IgG control on day 28 after infection." The rationale is that Il-27 mAb treated have "CD127+KLRG1-, CD127-KLRG1+, and CD127-KLRG1- cells" but Ctrl mAb treated mice also have a small population of these cells at day 28, it should be done as Ctrl are always necessary.
-We would like to emphasize that this figure is not a part of our main figures but shown as Fig. EV4 (comparable to a supplementary figure). This statement is invalid; a supplementary figure does not mean it is unimportant. This reviewer is well aware that EV figures are comparable to supplementary data. Do the authors mean that these figures should not be reviewed?
-Figure 7. Authors claimed, "The parasitemia was nearly undetectable levels in smear samples 2 months after infection with Pcc in both anti-IL-27 mAb-treated and untreated mice". Do they mean that this could be the reason for the superior immunity? What is then the benefit of treating with IL-27mAb? That is why I still think that they should have performed a challenge with heterologous parasites.
-Statistical analysis: Student tests or Anova analysis were performed, but there is no mention concerning the normality test. Are all the data following a normal distribution? For example, Figure 6B is analyzed with the Student test, but the data do not have a normal distribution, and the students between several groups are inappropriate. Please clarify
Minor points:
-About the difference between Ebi3-/- and IL27-/- infected mice. Would it be possible to verify that the difference is due to the lack of IL-35?

Referee #2 (Remarks for Author):

All my queries have been answered.

EMM-2023-17713-V3

Point-by-point response

Dear Referees,

We thank the reviewers for the comments to our manuscript and for giving an opportunity to revise our manuscript. We performed all the experiments the reviewer suggested and edited the figures as presented below.

The point-to-point responses to Reviewers are as follows:

Referee #1:
Major points:
-I understand the author's point about using heterologous challenges. Authors claim that "Given that the anti-IL-27 mAb treatment enhanced the immune response and further reduced the parasitemia after re-challenge to a level less than those of control mice," but they do not know what would have been happened with a homologous challenge, their raisoning is speculative only nevertheless if the 0.3 % parasitemia after re-challenge becomes 0 % giving complete protection that will make the difference.

We thank the reviewer for the comment. Following Reviewer 1's suggestion, we set up a new experiment. B6 mice were infected with Pcc and treated with anti-IL-27 Ab or IgG (n=5 mice/group). Sixty-three days after infection (the same period as the experiment in Figure 7), mice were re-challenged with homologous Pcc parasites, and the levels of parasitemia and weight of each mouse were monitored every 2 days. We used 1 x 10^6 iRBC, a higher dose (20x) than our standard Pcc infection experiments (5 x 10^4 iRBC) to increase the likelihood of detecting patent parasitemia. The result of this new experiment is shown in Figure 1a (below). The % parasitemias are also shown in Table 1a; note that 0% cannot be plotted the graph in log scale. We also monitored weight change of the mice.

Both IgG and anti-IL-27 mAb-treated mice exhibited less than 0.1% parasitemia and no weight loss, while control naive mice showed high parasitemia and severe loss of weight. As the reviewer alluded to, the level of parasitemia appears to be lower in

anti-IL-27 mAb treated mice when compared with IgG-treated mice. However, the levels of parasitemia in both groups were too low to evaluate the significant difference. Therefore, we added this point in the manuscript lines 362-366.

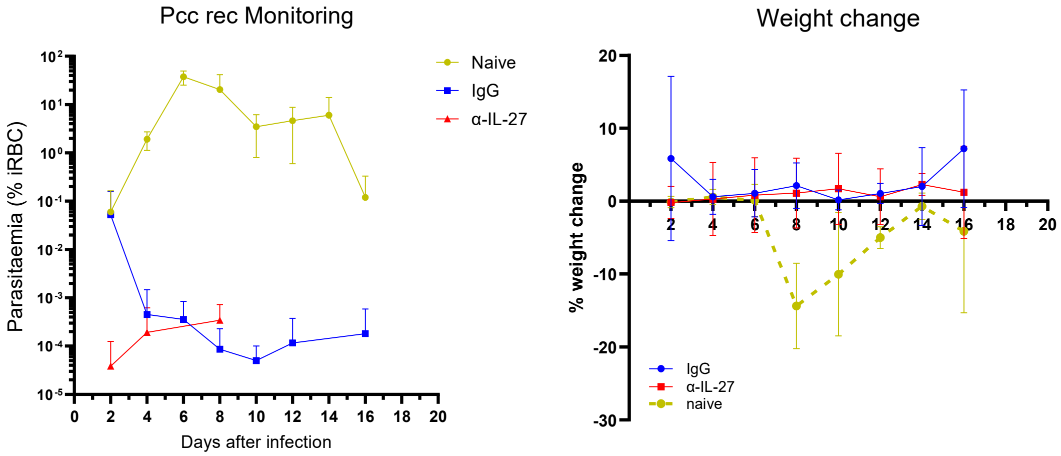

Figure 1a. B6 mice Pcc-infected and IgG (blue) and anti-IL-27 mAb (red)-treated mice as well as control naive mice were challenged with homologous Pcc (1 x 10^6 iRBC), and parasitemia and weight change were monitored every 2 days.

Table 1a: Parasitemia of Pcc-primed and IgG- and anti-IL-27 mAb treated mice as well as naive B6 mice after re-challenge with Pcc

Days post-infection	Naïve	IgG	α-IL-27
2	0.06000 ± 0.10393%	0.05200 ± 0.10546%	0.00004 ± 0.00009%
4	1.9200 ± 0.8058%	0.00046 ± 0.00102%	0.00020 ± 0.00043%
6	37.427 ± 12.202%	0.00036 ± 0.00049%	0
8	20.427 ± 21.253%	0.00009 ± 0.00015%	0.00035 ± 0.00039%
10	3.4933 ± 2.6964%	0.00005 ± 0.00005%	0
12	4.6433 ± 4.0442%	0.00012 ± 0.00027%	0
14	6.0033 ± 7.9960%	0	0
16	0.12000 ± 0.20785%	0.00019 ± 0.00041%	0

- That "majority of PbT-II cells" is not a sufficient reason to do not to make the gating on antigen-responding cells and then on KLRG1/cD127 or other markers. Authors should do that, which should be easy because they have the data. If they already have done that, they should indicate it on the main figures as gating

strategy is insufficient.

We thank Reviewer 1 for the comment. We did not have a complete data of flow cytometry with the simultaneous staining of CD11a/CD49d for all the analyzed markers, since we considered that majority of PbT-II cells are activated in Pcc-infected mice. Therefore, we set up new PbT-II-transfer experiments with new antibody panels including CD11a/CD49d combined with the other markers, and analyzed spleen cells on day 7 and 28 after infection.

The new data are presented in Fig. EV2. PbT-II cells were gated for $CD11a^{hi}CD49d^{hi}$ and $CD11a^{hi}CD49d^{lo}$ subpopulations, and their CD127/KLRG1, CXCR5/CXCR6, PD1/CXCR5, and T-bet/Tcf1 stainings are shown. On day 7 of infection $CD11a^{hi}CD49d^{hi}$ cells were mainly Th1 cells (70.4 - 87.4 % $T\text{-}bet^{+}$), while $CD11a^{hi}CD49d^{lo}$ population included Tfh cells (10.6 - 19.0 % $CXCR5^{+}$). In contrast, 28 days after infection, both $CD11a^{hi}CD49d^{hi}$ (91.0 % $T\text{-}bet^{+}$) and $CD11a^{hi}CD49d^{lo}$ (53.8 % $T\text{-}bet^{+}$) PbT-II cells in anti-IL-27 mAb-treated mice showed strong Th1 dominance, while those in IgG-treated mice showed reduced Th1 phenotypes even in $CD11a^{hi}CD49d^{hi}$ cells (41.0 % $T\text{-}bet^{+}$). These results are further explained in lines 201-210.

We maintained Figure 2 showing the phenotype of total PbT-II cells, since we believe that it is important to show the phenotypes of malaria-antigen specific $CD4^{+}$ T cells without bias.

- Authors claim, "We did not intend to compare the effect of anti-IL-27 mAb treatment with IgG control by scRNA-seq experiment and, therefore, did not need scRNA-seq data of IgG control on day 28 after infection." The rationale is that Il-27 mAb treated have "CD127+KLRG1-, CD127-KLRG1+, and CD127-KLRG1- cells" but Ctrl mAb treated mice also have a small population of these cells at day 28, it should be done as Ctrl are always necessary.

-We would like to emphasize that this figure is not a part of our main figures but shown as Fig. EV4 (comparable to a supplementary figure). This statement is invalid; a supplementary figure does not mean it is unimportant. This reviewer is well aware that EV figures are comparable to supplementary data. Do the authors mean that these figures should not be reviewed?

We appreciate Reviewer 1's suggestion to perform the IgG control experiment on day 28 of infection. Thus, we performed the IgG control experiment on day 28 of infection and included the data in Figure 5, Figure EV3, and Figure EV4. In Figure 5, we analyzed combined and dimensionally reduced data of PbT-II cells from IgG- and anti-IL-27-treated mice on day 28 of infection, and updated the results section in lines 259-294 accordingly. We showed the gating strategy and phenotypes of PbT-II cells used for single cell analysis in Figure EV3A-C. $CD4^+$ T cells from IgG- and anti-IL-27-treated mice were also mapped to $CD4^+$ T cell reference atlas (Andreatta *et al.* 2022) in Figure EV3 D, E.

-Figure 7. Authors claimed, "The parasitemia was nearly undetectable levels in smear samples 2 months after infection with Pcc in both anti-IL-27 mAb-treated and untreated mice". Do they mean that this could be the reason for the superior immunity? What is then the benefit of treating with IL-27mAb? That is why I still think that they should have performed a challenge with heterologous parasites.

As explained earlier, we performed homologous Pcc challenge infection, and observed barely detectable levels of parasitemia in both IgG- and anti-IL-27 mAb-treated mice. We explained the homologous challenge experiment in lines 362-366. We observed clear difference after heterologous challenge experiment, as we have shown in Fig. 7.

-Statistical analysis: Student tests or Anova analysis were performed, but there is no mention concerning the normality test. Are all the data following a normal distribution? For example, Figure 6B is analyzed with the Student test, but the data do not have a normal distribution, and the students between several groups are inappropriate. Please clarify

We thank Reviewer 1 for the comment. We reviewed our initial statistical usage, and modified them as explained in lines 655-662. We first assessed all the data for normality using the Shapiro–Wilk test and used two-tailed unpaired Student's t-tests for normally distributed data (parametric) and Mann-Whitney *U* test (non-parametric) for non-normally distributed data. For comparing more than 2 groups, one-way analysis of variance (ANOVA) with Tukey HSD post hoc tests (parametric) or Kruskal-Wallis test with Dunn's *post hoc* tests (non-parametric) were performed where applicable. The changes did not alter the original conclusions of the study.

Minor points:

-About the difference between Ebi3-/- and IL27-/- infected mice. Would it be possible to verify that the difference is due to the lack of IL-35?

We thank Reviewer 1 for the comment. Ebi3-/- mice lack IL-27, IL-35 and IL-39 dimer cytokines that are formed by pairing of EBI3 with 28, p35 and p19 subunits, respectively (Kourko et al, Front Oncol, 9: 969, 2019). IL-27-/- mice may lack not only IL-27 but also IL-30, which is potentially consisted with p28, p28/CLF or p28/sIL-6Rα (Kourko et al, Front Oncol, 9: 969, 2019). Therefore, the composition and functioning of these dimer cytokines are complex and is currently under investigation. The difference in $CD4^+$ T cell response during Pcc infection between Ebi3-/- and IL27-/- mice is interesting, but is beyond the scope of current study.

Referee #2:

All my queries have been answered.

We greatly appreciate the careful review. Thank you very much.

18th Sep 2023

Dear Prof. Yui,

Thank you for the submission of your revised manuscript to EMBO Molecular Medicine. I am pleased to inform you that we will be able to accept your manuscript pending the following final amendments:

1) In the main manuscript file, please do the following:
- Correct/answer the track changes suggested by our data editors by working from the attached document.
- Author contributions: Please remove it from the manuscript and specify author contributions in our submission system. CRediT has replaced the traditional author contributions section because it offers a systematic machine-readable author contributions format that allows for more effective research assessment. You are encouraged to use the free text boxes beneath each contributing author's name to add specific details on the author's contribution. More information is available in our guide to authors:
https://www.embopress.org/page/journal/17574684/authorguide#authorshipguidelines
- Data availability: Please use the following format to report the accession number of your data:

The datasets produced in this study are available in the following databases:
[data type]: [full name of the resource] [accession number/identifier] ([doi or URL or identifiers.org/DATABASE:ACCESSION])

Please check "Author Guidelines" for more information.
https://www.embopress.org/page/journal/17574684/authorguide#availabilityofpublishedmaterial
- Move main figure legends after references.
2) Funding: Please make sure that information about all sources of funding are complete in both our submission system and in the manuscript. Currently JSPS Short-Term Invitational Fellowship for Research in Japan is missing in our submission system.
3) Synopsis:
- Synopsis image: Please provide a striking image or visual abstract as a high-resolution jpeg file 550 px-wide x (250-400)-px high to illustrate your article.
- Synopsis text: Please provide a short standfirst (maximum of 300 characters, including space) as well as 2-5 one sentence bullet points that summarise the paper as a .doc file. Please write the bullet points to summarise the key NEW findings. They should be designed to be complementary to the abstract - i.e. not repeat the same text. We encourage inclusion of key acronyms and quantitative information (maximum of 30 words / bullet point). Please use the passive voice.
- Please check your synopsis text and image before submission with your revised manuscript. Please be aware that in the proof stage minor corrections only are allowed (e.g., typos).
4) For more information: This space should be used to list relevant web links for further consultation by our readers. Could you identify some relevant ones and provide such information as well? Some examples are patient associations, relevant databases, OMIM/proteins/genes links, author's websites, etc...
5) Source data: Please upload source data as one (zipped) file per figure, i.e. all the source data files related to a single figure should be zipped up into one zip file.
6) As part of the EMBO Publications transparent editorial process initiative (see our Editorial at http://embomolmed.embopress.org/content/2/9/329), EMBO Molecular Medicine will publish online a Review Process File (RPF) to accompany accepted manuscripts. This file will be published in conjunction with your paper and will include the anonymous referee reports, your point-by-point response and all pertinent correspondence relating to the manuscript. Let us know whether you agree with the publication of the RPF and as here, if you want to remove or not any figures from it prior to publication. Please note that the Authors checklist will be published at the end of the RPF.
7) Please provide a point-by-point letter INCLUDING my comments as well as the reviewer's reports and your detailed responses (as Word file).

I look forward to reading a new revised version of your manuscript as soon as possible.

Yours sincerely,

Zeljko Durdevic

Zeljko Durdevic
Editor
EMBO Molecular Medicine

*** Instructions to submit your revised manuscript ***

*** PLEASE NOTE *** As part of the EMBO Publications transparent editorial process initiative (see our Editorial at https://www.embopress.org/doi/pdf/10.1002/emmm.201000094), EMBO Molecular Medicine will publish online a Review Process File to accompany accepted manuscripts.

In the event of acceptance, this file will be published in conjunction with your paper and will include the anonymous referee reports, your point-by-point response and all pertinent correspondence relating to the manuscript. If you do NOT want this file to be published, please inform the editorial office at contact@embomolmed.org.

When submitting your revised manuscript, please include:

1) a .docx formatted version of the manuscript text (including Figure legends and tables)

2) Separate figure files*

3) supplemental information as Expanded View and/or Appendix. Please carefully check the authors guidelines for formatting Expanded view and Appendix figures and tables at https://www.embopress.org/page/journal/17574684/authorguide#expandedview

4) a letter INCLUDING the reviewer's reports and your detailed responses to their comments (as Word file).

5) The paper explained: EMBO Molecular Medicine articles are accompanied by a summary of the articles to emphasize the major findings in the paper and their medical implications for the non-specialist reader. Please provide a draft summary of your article highlighting
- the medical issue you are addressing,
- the results obtained and
- their clinical impact.
This may be edited to ensure that readers understand the significance and context of the research.
Please refer to any of our published articles for an example.

6) For more information: There is space at the end of each article to list relevant web links for further consultation by our readers. Could you identify some relevant ones and provide such information as well? Some examples are patient associations, relevant databases, OMIM/proteins/genes links, author's websites, etc...

7) Author contributions: the contribution of every author must be detailed in a separate section.

8) EMBO Molecular Medicine now requires a complete author checklist (https://www.embopress.org/page/journal/17574684/authorguide) to be submitted with all revised manuscripts. Please use the checklist as guideline for the sort of information we need WITHIN the manuscript. The checklist should only be filled with page numbers were the information can be found. This is particularly important for animal reporting, antibody dilutions (missing) and exact values and n that should be indicted instead of a range.

9) Every published paper now includes a 'Synopsis' to further enhance discoverability. Synopses are displayed on the journal webpage and are freely accessible to all readers. They include a short stand first (maximum of 300 characters, including space) as well as 2-5 one sentence bullet points that summarise the paper. Please write the bullet points to summarise the key NEW findings. They should be designed to be complementary to the abstract - i.e. not repeat the same text. We encourage inclusion of key acronyms and quantitative information (maximum of 30 words / bullet point). Please use the passive voice. Please attach these in a separate file or send them by email, we will incorporate them accordingly.

You are also welcome to suggest a striking image or visual abstract to illustrate your article. If you do please provide a jpeg file 550 px-wide x 400-px high.

10) A Conflict of Interest statement should be provided in the main text

11) Please note that we now mandate that all corresponding authors list an ORCID digital identifier. This takes <90 seconds to complete. We encourage all authors to supply an ORCID identifier, which will be linked to their name for unambiguous name

identification.

Currently, our records indicate that the ORCID for your account is 0000-0003-1130-2496.

Please click the link below to modify this ORCID:
Link Not Available

12) The system will prompt you to fill in your funding and payment information. This will allow Wiley to send you a quote for the article processing charge (APC) in case of acceptance. This quote takes into account any reduction or fee waivers that you may be eligible for. Authors do not need to pay any fees before their manuscript is accepted and transferred to our publisher.

*Additional important information regarding Figures

Each figure should be given in a separate file and should have the following resolution:
Graphs 800-1,200 DPI
Photos 400-800 DPI
Colour (only CMYK) 300-400 DPI"

Figures are not edited by the production team. All lettering should be the same size and style; figure panels should be indicated by capital letters (A, B, C etc). Gridlines are not allowed except for log plots. Figures should be numbered in the order of their appearance in the text with Arabic numerals. Each Figure must have a separate legend and a caption is needed for each panel.

*Additional important information regarding figures and illustrations can be found at https://bit.ly/EMBOPressFigurePreparationGuideline. See also figure legend preparation guidelines: https://www.embopress.org/page/journal/17574684/authorguide#figureformat

The system will prompt you to fill in your funding and payment information. This will allow Wiley to send you a quote for the article processing charge (APC) in case of acceptance. This quote takes into account any reduction or fee waivers that you may be eligible for. Authors do not need to pay any fees before their manuscript is accepted and transferred to our publisher.

***** Reviewer's comments *****

Referee #1 (Comments on Novelty/Model System for Author):

I have no further questions. The authors answered my questions.

Referee #1 (Remarks for Author):

The authors answered all my questions, did the experiments, and performed the statistical analysis correctly. I don't have further questions.

The authors addressed the remaining editorial issues.

28th Sep 2023

Dear Prof. Yui,

We are pleased to inform you that your manuscript is accepted for publication and is now being sent to our publisher to be included in the next available issue of EMBO Molecular Medicine.

Please read below for additional IMPORTANT information regarding your article, its publication and the production process.

Congratulations on your interesting work,

Zeljko Durdevic

Zeljko Durdevic
Editor
EMBO Molecular Medicine

Follow us on Twitter @EmboMolMed
Sign up for eTOCs at embopress.org/alertsfeeds

*** *** *** IMPORTANT INFORMATION *** *** ***

SPEED OF PUBLICATION
The journal aims for rapid publication of papers, using using the advance online publication "Early View" to expedite the process: A properly copy-edited and formatted version will be published as "Early View" after the proofs have been corrected. Please help the Editors and publisher avoid delays by providing e-mail address(es), telephone and fax numbers at which author(s) can be contacted.

Should you be planning a Press Release on your article, please get in contact with embomolmed@wiley.com as early as possible, in order to coordinate publication and release dates.

LICENSE AND PAYMENT:

All articles published in EMBO Molecular Medicine are fully open access: immediately and freely available to read, download and share.

EMBO Molecular Medicine charges an article processing charge (APC) to cover the publication costs. You, as the corresponding author for this manuscript, should have already received a quote with the article processing fee separately. Please let us know in case this quote has not been received.

Once your article is at Wiley for editorial production you will receive an email from Wiley's Author Services system, which will ask you to log in and will present you with the publication license form for completion. Within the same system the publication fee can be paid by credit card, an invoice, pro forma invoice or purchase order can be requested.

Payment of the publication charge and the signed Open Access Agreement form must be received before the article can be published online.

PROOFS

You will receive the proofs by e-mail approximately 2 weeks after all relevant files have been sent o our Production Office. Please return them within 48 hours and if there should be any problems, please contact the production office at embopressproduction@wiley.com.

Please inform us if there is likely to be any difficulty in reaching you at the above address at that time. Failure to meet our

deadlines may result in a delay of publication.

All further communications concerning your paper proofs should quote reference number EMM-2023-17713-V4 and be directed to the production office at embopressproduction@wiley.com.

Thank you,

Zeljko Durdevic
Editor
EMBO Molecular Medicine

EMBO Press Author Checklist

Corresponding Author Name: Katsuyuki Yui	
Journal Submitted to: EMBO Molecular Medicine	
Manuscript Number: EMM-2023-17713-V3	

USEFUL LINKS FOR COMPLETING THIS FORM
The EMBO Journal - Author Guidelines
EMBO Reports - Author Guidelines
Molecular Systems Biology - Author Guidelines
EMBO Molecular Medicine - Author Guidelines

Reporting Checklist for Life Science Articles (updated January
This checklist is adapted from Materials Design Analysis Reporting (MDAR) Checklist for Authors. MDAR establishes a minimum set of requirements in transparent reporting in the life sciences (see Statement of Task: 10.31222/osf.io/9sm4x). Please follow the journal's guidelines in preparing your
Please note that a copy of this checklist will be published alongside your article.

Abridged guidelines for figures
1. Data
The data shown in figures should satisfy the following conditions:
- the data were obtained and processed according to the field's best practice and are presented to reflect the results of the experiments in an accurate and unbiased manner.
- ideally, figure panels should include only measurements that are directly comparable to each other and obtained with the same assay.
- plots include clearly labeled error bars for independent experiments and sample sizes. Unless justified, error bars should not be shown for technical
- if n<5, the individual data points from each experiment should be plotted. Any statistical test employed should be justified.
- Source Data should be included to report the data underlying figures according to the guidelines set out in the authorship guidelines on Data

2. Captions
Each figure caption should contain the following information, for each panel where they are relevant:
- a specification of the experimental system investigated (eg cell line, species name).
- the assay(s) and method(s) used to carry out the reported observations and measurements.
- an explicit mention of the biological and chemical entity(ies) that are being measured.
- an explicit mention of the biological and chemical entity(ies) that are altered/varied/perturbed in a controlled manner.
- the exact sample size (n) for each experimental group/condition, given as a number, not a range;
- a description of the sample collection allowing the reader to understand whether the samples represent technical or biological replicates (including how many animals, litters, cultures, etc.).
- a statement of how many times the experiment shown was independently replicated in the laboratory.
- definitions of statistical methods and measures:
  - common tests, such as t-test (please specify whether paired vs. unpaired), simple χ2 tests, Wilcoxon and Mann-Whitney tests, can be unambiguously identified by name only, but more complex techniques should be described in the methods section;
  - are tests one-sided or two-sided?
  - are there adjustments for multiple comparisons?
  - exact statistical test results, e.g., P values = x but not P values < x;
  - definition of 'center values' as median or average;
  - definition of error bars as s.d. or s.e.m.

Please complete ALL of the questions below.
Select "Not Applicable" only when the requested information is not relevant for your study.

Materials

Newly Created Materials	Information included in the manuscript?	In which section is the information available? (Reagents and Tools Table, Materials and Methods, Figures, Data Availability Section)
New materials and reagents need to be available; do any restrictions apply?	Not Applicable	

Antibodies	Information included in the manuscript?	In which section is the information available? (Reagents and Tools Table, Materials and Methods, Figures, Data Availability Section)
For **antibodies** provide the following information:<br>- Commercial antibodies: RRID (if possible) or supplier name, catalogue number and or/clone number<br>- Non-commercial: RRID or citation	Yes	Reagents and Tools, Materials and Methods

DNA and RNA sequences	Information included in the manuscript?	In which section is the information available? (Reagents and Tools Table, Materials and Methods, Figures, Data Availability Section)
**Short novel DNA or RNA including primers, probes**: provide the sequences.	Not Applicable	

Cell materials	Information included in the manuscript?	In which section is the information available? (Reagents and Tools Table, Materials and Methods, Figures, Data Availability Section)
**Cell lines:** Provide species information, strain. Provide accession number in repository **OR** supplier name, catalog number, clone number, and/**OR** RRID.	Not Applicable	
**Primary cultures:** Provide species, strain, sex of origin, genetic modification status.	Not Applicable	
Report if the cell lines were recently **authenticated** (e.g., by STR profiling) and tested for mycoplasma contamination.	Not Applicable	

Experimental animals	Information included in the manuscript?	In which section is the information available? (Reagents and Tools Table, Materials and Methods, Figures, Data Availability Section)
**Laboratory animals or Model organisms:** Provide species, strain, sex, age, genetic modification status. Provide accession number in repository **OR** supplier name, catalog number, clone number, **OR** RRID.	Yes	Reagents and Tools table, Materials and Methods
**Animal observed in or captured from the field:** Provide species, sex, and age where possible.	Not Applicable	
Please detail **housing and husbandry conditions**.	Yes	Materials and Methods

Plants and microbes	Information included in the manuscript?	In which section is the information available? (Reagents and Tools Table, Materials and Methods, Figures, Data Availability Section)
**Plants:** provide species and strain, ecotype and cultivar where relevant, unique accession number if available, and source (including location for collected wild specimens).	Not Applicable	
**Microbes:** provide species and strain, unique accession number if available, and source.	Yes	Materials and Methods

Human research participants	Information included in the manuscript?	In which section is the information available? (Reagents and Tools Table, Materials and Methods, Figures, Data Availability Section)
If collected and within the bounds of privacy constraints report on age, sex and gender or ethnicity for all study participants.	Not Applicable	

Core facilities	Information included in the manuscript?	In which section is the information available? (Reagents and Tools Table, Materials and Methods, Figures, Data Availability Section)
If your work benefited from core facilities, was their service mentioned in the acknowledgments section?	Not Applicable	

Design

Study protocol	Information included in the manuscript?	In which section is the information available? (Reagents and Tools Table, Materials and Methods, Figures, Data Availability Section)
If study protocol has been **pre-registered, provide DOI in the manuscript**. For clinical trials, provide the trial registration number **OR** cite DOI.	Not Applicable	
Report the **clinical trial registration number** (at ClinicalTrials.gov or equivalent), where applicable.	Not Applicable	

Laboratory protocol	Information included in the manuscript?	In which section is the information available? (Reagents and Tools Table, Materials and Methods, Figures, Data Availability Section)
Provide DOI OR other citation details if **external detailed step-by-step protocols** are available.	Not Applicable	

Experimental study design and statistics	Information included in the manuscript?	In which section is the information available? (Reagents and Tools Table, Materials and Methods, Figures, Data Availability Section)
Include a statement about **sample size** estimate even if no statistical methods were used.	Not Applicable	
Were any steps taken to minimize the effects of subjective bias when allocating animals/samples to treatment (e.g. **randomization procedure**)? If yes, have they been described?	Not Applicable	
Include a statement about **blinding** even if no blinding was done.	Not Applicable	
Describe **inclusion/exclusion criteria** if samples or animals were excluded from the analysis. Were the criteria pre-established?<br><br>If sample or data points were omitted from analysis, report if this was due to attrition or intentional exclusion and provide justification.	Not Applicable	
For every figure, are **statistical tests** justified as appropriate? Do the data meet the assumptions of the tests (e.g., normal distribution)? Describe any methods used to assess it. Is there an estimate of variation within each group of data? Is the variance similar between the groups that are being statistically compared?	Yes	Materials and Methods

Sample definition and in-laboratory replication	Information included in the manuscript?	In which section is the information available? (Reagents and Tools Table, Materials and Methods, Figures, Data Availability Section)
In the figure legends: state number of times the experiment was **replicated** in laboratory.	Yes	Figures
In the figure legends: define whether data describe **technical or biological replicates**.	Yes	Figures

Ethics

Ethics	Information included in the manuscript?	In which section is the information available? (Reagents and Tools Table, Materials and Methods, Figures, Data Availability Section)
Studies involving **human participants**: State details of **authority granting ethics approval** (IRB or equivalent committee(s), provide reference number for approval.	Not Applicable	
Studies involving **human participants**: Include a statement confirming that **informed consent** was obtained from all subjects and that the experiments conformed to the principles set out in the WMA Declaration of Helsinki and the Department of Health and Human Services Belmont Report.	Not Applicable	
Studies involving **human participants: For** publication of **patient photos**, include a statement confirming that consent to publish was obtained.	Not Applicable	
Studies involving experimental **animals**: State details of **authority granting ethics approval** (IRB or equivalent committee(s), provide reference number for approval. Include a statement of compliance with ethical regulations.	Yes	Materials and Methods
Studies involving **specimen and field samples:** State if relevant **permits** obtained, provide details of authority approving study; if none were required, explain why.	Not Applicable	

Dual Use Research of Concern (DURC)	Information included in the manuscript?	In which section is the information available? (Reagents and Tools Table, Materials and Methods, Figures, Data Availability Section)
Could your study fall under dual use research restrictions? Please check biosecurity documents and list of **select agents and toxins** (CDC): https://www.selectagents.gov/sat/list.htm	Not Applicable	
If you used a select agent, is the security level of the lab appropriate and reported in the manuscript?	Not Applicable	
If a study is subject to dual use research of concern regulations, is the name of the **authority granting approval and reference number** for the regulatory approval provided in the manuscript?	Not Applicable	

Reporting

The MDAR framework recommends adoption of discipline-specific guidelines, established and endorsed through community initiatives. Journals have their own policy about requiring specific guidelines and recommendations to complement MDAR.

Adherence to community standards	Information included in the manuscript?	In which section is the information available? (Reagents and Tools Table, Materials and Methods, Figures, Data Availability Section)
State if relevant guidelines or checklists (e.g., **ICMJE, MIBBI, ARRIVE, PRISMA**) have been followed or provided.	Not Applicable	
For **tumor marker prognostic studies,** we recommend that you follow the **REMARK** reporting guidelines (see link list at top right). See author guidelines, under 'Reporting Guidelines'. Please confirm you have followed these guidelines.	Not Applicable	
For **phase II and III randomized controlled trials**, please refer to the **CONSORT** flow diagram (see link list at top right) and submit the CONSORT checklist (see link list at top right) with your submission. See author guidelines, under 'Reporting Guidelines'. Please confirm you have submitted this list.	Not Applicable	

Data Availability

Data availability	Information included in the manuscript?	In which section is the information available? (Reagents and Tools Table, Materials and Methods, Figures, Data Availability Section)
Have **primary datasets** been deposited according to the journal's guidelines (see 'Data Deposition' section) and the respective accession numbers provided in the Data Availability Section?	Yes	Materials and Methods
Were **human clinical and genomic datasets** deposited in a public access-controlled repository in accordance to ethical obligations to the patients and to the applicable consent agreement?	Not Applicable	
Are **computational models** that are central and integral to a study available without restrictions in a machine-readable form? Were the relevant accession numbers or links provided?	Not Applicable	
If publicly available data were reused, provide the respective **data citations in the reference list**.	Not Applicable	
